# Supplementary material for: Tolerance of Pseudomonas strain to the 2,4-D herbicide through a peroxidase system
Source: PLoS One. 2021 Dec 2;16(12):e0257263. doi: 10.1371/journal.pone.0257263 (PMC8638965; doi:10.1371/journal.pone.0257263)
Supplement: S1 Appendix — (PDF) [file pone.0257263.s001.pdf]

Growth curve
(2way ANOVA) Multiple Comparisons.

	2way ANOVA<br>Multiple comparisons	A<br>Data Set-A<br>Y	B<br>Data Set-B<br>Y	C<br>Data Set-C<br>Y
1	Compare cell means regardless of rows and columns			
2				
3	Number of families	1		
4	Number of comparisons per family	1953		
5	Alpha	0.05		
6				
7	Tukey's multiple comparisons test	Mean Diff.	95% CI of diff.	Significant?
8				
9	Row 1:**0 X** vs. Row 1:**10 X**	0.0	-0.4269 to 0.4269	No
10	Row 1:**0 X** vs. Row 1:**25 X**	0.0	-0.4269 to 0.4269	No
11	Row 1:**0 X** vs. Row 2:**0 X**	0.0370	-0.3899 to 0.4639	No
12	Row 1:**0 X** vs. Row 2:**10 X**	0.03333	-0.3936 to 0.4602	No
13	Row 1:**0 X** vs. Row 2:**25 X**	0.03533	-0.3916 to 0.4622	No
14	Row 1:**0 X** vs. Row 3:**0 X**	0.02133	-0.4056 to 0.4482	No
15	Row 1:**0 X** vs. Row 3:**10 X**	0.02133	-0.4056 to 0.4482	No
16	Row 1:**0 X** vs. Row 3:**25 X**	0.02367	-0.4032 to 0.4506	No
17	Row 1:**0 X** vs. Row 4:**0 X**	-0.0140	-0.4409 to 0.4129	No
18	Row 1:**0 X** vs. Row 4:**10 X**	-0.007667	-0.4346 to 0.4192	No
19	Row 1:**0 X** vs. Row 4:**25 X**	-0.02033	-0.4472 to 0.4066	No
20	Row 1:**0 X** vs. Row 5:**0 X**	-0.2787	-0.7056 to 0.1482	No
21	Row 1:**0 X** vs. Row 5:**10 X**	-0.2460	-0.6729 to 0.1809	No
22	Row 1:**0 X** vs. Row 5:**25 X**	-0.2240	-0.6509 to 0.2029	No
23	Row 1:**0 X** vs. Row 6:**0 X**	-0.8227	-1.250 to -0.3958	Yes
24	Row 1:**0 X** vs. Row 6:**10 X**	-0.7180	-1.145 to -0.2911	Yes
25	Row 1:**0 X** vs. Row 6:**25 X**	-0.6897	-1.117 to -0.2628	Yes
26	Row 1:**0 X** vs. Row 7:**0 X**	-1.202	-1.629 to -0.7751	Yes
27	Row 1:**0 X** vs. Row 7:**10 X**	-1.125	-1.552 to -0.6978	Yes
28	Row 1:**0 X** vs. Row 7:**25 X**	-1.002	-1.429 to -0.5751	Yes
29	Row 1:**0 X** vs. Row 8:**0 X**	-1.433	-1.860 to -1.006	Yes
30	Row 1:**0 X** vs. Row 8:**10 X**	-1.537	-1.964 to -1.110	Yes
31	Row 1:**0 X** vs. Row 8:**25 X**	-1.480	-1.907 to -1.053	Yes
32	Row 1:**0 X** vs. Row 9:**0 X**	-1.788	-2.215 to -1.361	Yes
33	Row 1:**0 X** vs. Row 9:**10 X**	-1.772	-2.199 to -1.345	Yes
34	Row 1:**0 X** vs. Row 9:**25 X**	-1.588	-2.015 to -1.161	Yes
35	Row 1:**0 X** vs. Row 10:**0 X**	-2.036	-2.463 to -1.609	Yes
36	Row 1:**0 X** vs. Row 10:**10 X**	-2.028	-2.455 to -1.601	Yes
37	Row 1:**0 X** vs. Row 10:**25 X**	-1.861	-2.288 to -1.434	Yes
38	Row 1:**0 X** vs. Row 11:**0 X**	-2.794	-3.221 to -2.367	Yes
39	Row 1:**0 X** vs. Row 11:**10 X**	-2.146	-2.623 to -1.669	Yes
40	Row 1:**0 X** vs. Row 11:**25 X**	-2.089	-2.516 to -1.662	Yes
41	Row 1:**0 X** vs. 11:**0 X**	-2.888	-3.315 to -2.461	Yes
42	Row 1:**0 X** vs. 11:**10 X**	-2.462	-2.939 to -1.985	Yes
43	Row 1:**0 X** vs. 11:**25 X**	-2.393	-2.820 to -1.966	Yes
44	Row 1:**0 X** vs. Row 13:**0 X**	-2.857	-3.284 to -2.430	Yes
45	Row 1:**0 X** vs. Row 13:**10 X**	-2.490	-2.967 to -2.013	Yes

	2way ANOVA<br>Multiple comparisons	A<br>Data Set-A<br>Y	B<br>Data Set-B<br>Y	C<br>Data Set-C<br>Y
**46**	Row 1:**0 X** vs. Row 13:**25 X**	-2.499	-2.926 to -2.072	Yes
**47**	Row 1:**0 X** vs. Row 14:**0 X**	-2.907	-3.334 to -2.480	Yes
**48**	Row 1:**0 X** vs. Row 14:**10 X**	-2.683	-3.110 to -2.256	Yes
**49**	Row 1:**0 X** vs. Row 14:**25 X**	-2.643	-3.070 to -2.216	Yes
**50**	Row 1:**0 X** vs. 14:**0 X**	-3.501	-3.928 to -3.074	Yes
**51**	Row 1:**0 X** vs. 14:**10 X**	-2.898	-3.375 to -2.421	Yes
**52**	Row 1:**0 X** vs. 14:**25 X**	-2.534	-3.011 to -2.057	Yes
**53**	Row 1:**0 X** vs. Row 16:**0 X**	-3.614	-4.091 to -3.137	Yes
**54**	Row 1:**0 X** vs. Row 16:**10 X**	-2.982	-3.459 to -2.505	Yes
**55**	Row 1:**0 X** vs. Row 16:**25 X**	-2.127	-2.604 to -1.650	Yes
**56**	Row 1:**0 X** vs. Row 17:**0 X**	-3.240	-3.717 to -2.762	Yes
**57**	Row 1:**0 X** vs. Row 17:**10 X**	-3.000	-3.477 to -2.522	Yes
**58**	Row 1:**0 X** vs. Row 17:**25 X**	-2.293	-2.770 to -1.816	Yes
**59**	Row 1:**0 X** vs. 17:**0 X**	-3.501	-3.978 to -3.023	Yes
**60**	Row 1:**0 X** vs. 17:**10 X**	-3.294	-3.771 to -2.817	Yes
**61**	Row 1:**0 X** vs. 17:**25 X**	-2.203	-2.630 to -1.776	Yes
**62**	Row 1:**0 X** vs. Row 19:**0 X**	-3.597	-4.074 to -3.119	Yes
**63**	Row 1:**0 X** vs. Row 19:**10 X**	-3.297	-3.774 to -2.819	Yes
**64**	Row 1:**0 X** vs. Row 19:**25 X**	-2.133	-2.560 to -1.706	Yes
**65**	Row 1:**0 X** vs. Row 20:**0 X**	-3.649	-4.126 to -3.172	Yes
**66**	Row 1:**0 X** vs. Row 20:**10 X**	-3.190	-3.667 to -2.713	Yes
**67**	Row 1:**0 X** vs. Row 20:**25 X**	-2.077	-2.504 to -1.650	Yes
**68**	Row 1:**0 X** vs. Row 21:**0 X**	-3.006	-3.483 to -2.529	Yes
**69**	Row 1:**0 X** vs. Row 21:**10 X**	-2.576	-3.053 to -2.098	Yes
**70**	Row 1:**0 X** vs. Row 21:**25 X**	-2.104	-2.531 to -1.677	Yes
**71**	Row 1:**10 X** vs. Row 1:**25 X**	0.0	-0.4269 to 0.4269	No
**72**	Row 1:**10 X** vs. Row 2:**0 X**	0.0370	-0.3899 to 0.4639	No
**73**	Row 1:**10 X** vs. Row 2:**10 X**	0.03333	-0.3936 to 0.4602	No
**74**	Row 1:**10 X** vs. Row 2:**25 X**	0.03533	-0.3916 to 0.4622	No
**75**	Row 1:**10 X** vs. Row 3:**0 X**	0.02133	-0.4056 to 0.4482	No
**76**	Row 1:**10 X** vs. Row 3:**10 X**	0.02133	-0.4056 to 0.4482	No
**77**	Row 1:**10 X** vs. Row 3:**25 X**	0.02367	-0.4032 to 0.4506	No
**78**	Row 1:**10 X** vs. Row 4:**0 X**	-0.0140	-0.4409 to 0.4129	No
**79**	Row 1:**10 X** vs. Row 4:**10 X**	-0.007667	-0.4346 to 0.4192	No
**80**	Row 1:**10 X** vs. Row 4:**25 X**	-0.02033	-0.4472 to 0.4066	No
**81**	Row 1:**10 X** vs. Row 5:**0 X**	-0.2787	-0.7056 to 0.1482	No
**82**	Row 1:**10 X** vs. Row 5:**10 X**	-0.2460	-0.6729 to 0.1809	No
**83**	Row 1:**10 X** vs. Row 5:**25 X**	-0.2240	-0.6509 to 0.2029	No
**84**	Row 1:**10 X** vs. Row 6:**0 X**	-0.8227	-1.250 to -0.3958	Yes
**85**	Row 1:**10 X** vs. Row 6:**10 X**	-0.7180	-1.145 to -0.2911	Yes
**86**	Row 1:**10 X** vs. Row 6:**25 X**	-0.6897	-1.117 to -0.2628	Yes
**87**	Row 1:**10 X** vs. Row 7:**0 X**	-1.202	-1.629 to -0.7751	Yes
**88**	Row 1:**10 X** vs. Row 7:**10 X**	-1.125	-1.552 to -0.6978	Yes
**89**	Row 1:**10 X** vs. Row 7:**25 X**	-1.002	-1.429 to -0.5751	Yes
**90**	Row 1:**10 X** vs. Row 8:**0 X**	-1.433	-1.860 to -1.006	Yes

	2way ANOVA<br>Multiple comparisons	A<br>Data Set-A<br>Y	B<br>Data Set-B<br>Y	C<br>Data Set-C<br>Y
**91**	Row 1:**10 X** vs. Row 8:**10 X**	-1.537	-1.964 to -1.110	Yes
**92**	Row 1:**10 X** vs. Row 8:**25 X**	-1.480	-1.907 to -1.053	Yes
**93**	Row 1:**10 X** vs. Row 9:**0 X**	-1.788	-2.215 to -1.361	Yes
**94**	Row 1:**10 X** vs. Row 9:**10 X**	-1.772	-2.199 to -1.345	Yes
**95**	Row 1:**10 X** vs. Row 9:**25 X**	-1.588	-2.015 to -1.161	Yes
**96**	Row 1:**10 X** vs. Row 10:**0 X**	-2.036	-2.463 to -1.609	Yes
**97**	Row 1:**10 X** vs. Row 10:**10 X**	-2.028	-2.455 to -1.601	Yes
**98**	Row 1:**10 X** vs. Row 10:**25 X**	-1.861	-2.288 to -1.434	Yes
**99**	Row 1:**10 X** vs. Row 11:**0 X**	-2.794	-3.221 to -2.367	Yes
**100**	Row 1:**10 X** vs. Row 11:**10 X**	-2.146	-2.623 to -1.669	Yes
**101**	Row 1:**10 X** vs. Row 11:**25 X**	-2.089	-2.516 to -1.662	Yes
**102**	Row 1:**10 X** vs. 11:**0 X**	-2.888	-3.315 to -2.461	Yes
**103**	Row 1:**10 X** vs. 11:**10 X**	-2.462	-2.939 to -1.985	Yes
**104**	Row 1:**10 X** vs. 11:**25 X**	-2.393	-2.820 to -1.966	Yes
**105**	Row 1:**10 X** vs. Row 13:**0 X**	-2.857	-3.284 to -2.430	Yes
**106**	Row 1:**10 X** vs. Row 13:**10 X**	-2.490	-2.967 to -2.013	Yes
**107**	Row 1:**10 X** vs. Row 13:**25 X**	-2.499	-2.926 to -2.072	Yes
**108**	Row 1:**10 X** vs. Row 14:**0 X**	-2.907	-3.334 to -2.480	Yes
**109**	Row 1:**10 X** vs. Row 14:**10 X**	-2.683	-3.110 to -2.256	Yes
**110**	Row 1:**10 X** vs. Row 14:**25 X**	-2.643	-3.070 to -2.216	Yes
**111**	Row 1:**10 X** vs. 14:**0 X**	-3.501	-3.928 to -3.074	Yes
**112**	Row 1:**10 X** vs. 14:**10 X**	-2.898	-3.375 to -2.421	Yes
**113**	Row 1:**10 X** vs. 14:**25 X**	-2.534	-3.011 to -2.057	Yes
**114**	Row 1:**10 X** vs. Row 16:**0 X**	-3.614	-4.091 to -3.137	Yes
**115**	Row 1:**10 X** vs. Row 16:**10 X**	-2.982	-3.459 to -2.505	Yes
**116**	Row 1:**10 X** vs. Row 16:**25 X**	-2.127	-2.604 to -1.650	Yes
**117**	Row 1:**10 X** vs. Row 17:**0 X**	-3.240	-3.717 to -2.762	Yes
**118**	Row 1:**10 X** vs. Row 17:**10 X**	-3.000	-3.477 to -2.522	Yes
**119**	Row 1:**10 X** vs. Row 17:**25 X**	-2.293	-2.770 to -1.816	Yes
**120**	Row 1:**10 X** vs. 17:**0 X**	-3.501	-3.978 to -3.023	Yes
**121**	Row 1:**10 X** vs. 17:**10 X**	-3.294	-3.771 to -2.817	Yes
**122**	Row 1:**10 X** vs. 17:**25 X**	-2.203	-2.630 to -1.776	Yes
**123**	Row 1:**10 X** vs. Row 19:**0 X**	-3.597	-4.074 to -3.119	Yes
**124**	Row 1:**10 X** vs. Row 19:**10 X**	-3.297	-3.774 to -2.819	Yes
**125**	Row 1:**10 X** vs. Row 19:**25 X**	-2.133	-2.560 to -1.706	Yes
**126**	Row 1:**10 X** vs. Row 20:**0 X**	-3.649	-4.126 to -3.172	Yes
**127**	Row 1:**10 X** vs. Row 20:**10 X**	-3.190	-3.667 to -2.713	Yes
**128**	Row 1:**10 X** vs. Row 20:**25 X**	-2.077	-2.504 to -1.650	Yes
**129**	Row 1:**10 X** vs. Row 21:**0 X**	-3.006	-3.483 to -2.529	Yes
**130**	Row 1:**10 X** vs. Row 21:**10 X**	-2.576	-3.053 to -2.098	Yes
**131**	Row 1:**10 X** vs. Row 21:**25 X**	-2.104	-2.531 to -1.677	Yes
**132**	Row 1:**25 X** vs. Row 2:**0 X**	0.0370	-0.3899 to 0.4639	No
**133**	Row 1:**25 X** vs. Row 2:**10 X**	0.03333	-0.3936 to 0.4602	No
**134**	Row 1:**25 X** vs. Row 2:**25 X**	0.03533	-0.3916 to 0.4622	No
**135**	Row 1:**25 X** vs. Row 3:**0 X**	0.02133	-0.4056 to 0.4482	No

	**2way ANOVA**<br>Multiple comparisons	**A**<br>Data Set-A<br>**Y**	**B**<br>Data Set-B<br>**Y**	**C**<br>Data Set-C<br>**Y**
**136**	Row 1:**25 X** vs. Row 3:**10 X**	0.02133	-0.4056 to 0.4482	No
**137**	Row 1:**25 X** vs. Row 3:**25 X**	0.02367	-0.4032 to 0.4506	No
**138**	Row 1:**25 X** vs. Row 4:**0 X**	-0.0140	-0.4409 to 0.4129	No
**139**	Row 1:**25 X** vs. Row 4:**10 X**	-0.007667	-0.4346 to 0.4192	No
**140**	Row 1:**25 X** vs. Row 4:**25 X**	-0.02033	-0.4472 to 0.4066	No
**141**	Row 1:**25 X** vs. Row 5:**0 X**	-0.2787	-0.7056 to 0.1482	No
**142**	Row 1:**25 X** vs. Row 5:**10 X**	-0.2460	-0.6729 to 0.1809	No
**143**	Row 1:**25 X** vs. Row 5:**25 X**	-0.2240	-0.6509 to 0.2029	No
**144**	Row 1:**25 X** vs. Row 6:**0 X**	-0.8227	-1.250 to -0.3958	Yes
**145**	Row 1:**25 X** vs. Row 6:**10 X**	-0.7180	-1.145 to -0.2911	Yes
**146**	Row 1:**25 X** vs. Row 6:**25 X**	-0.6897	-1.117 to -0.2628	Yes
**147**	Row 1:**25 X** vs. Row 7:**0 X**	-1.202	-1.629 to -0.7751	Yes
**148**	Row 1:**25 X** vs. Row 7:**10 X**	-1.125	-1.552 to -0.6978	Yes
**149**	Row 1:**25 X** vs. Row 7:**25 X**	-1.002	-1.429 to -0.5751	Yes
**150**	Row 1:**25 X** vs. Row 8:**0 X**	-1.433	-1.860 to -1.006	Yes
**151**	Row 1:**25 X** vs. Row 8:**10 X**	-1.537	-1.964 to -1.110	Yes
**152**	Row 1:**25 X** vs. Row 8:**25 X**	-1.480	-1.907 to -1.053	Yes
**153**	Row 1:**25 X** vs. Row 9:**0 X**	-1.788	-2.215 to -1.361	Yes
**154**	Row 1:**25 X** vs. Row 9:**10 X**	-1.772	-2.199 to -1.345	Yes
**155**	Row 1:**25 X** vs. Row 9:**25 X**	-1.588	-2.015 to -1.161	Yes
**156**	Row 1:**25 X** vs. Row 10:**0 X**	-2.036	-2.463 to -1.609	Yes
**157**	Row 1:**25 X** vs. Row 10:**10 X**	-2.028	-2.455 to -1.601	Yes
**158**	Row 1:**25 X** vs. Row 10:**25 X**	-1.861	-2.288 to -1.434	Yes
**159**	Row 1:**25 X** vs. Row 11:**0 X**	-2.794	-3.221 to -2.367	Yes
**160**	Row 1:**25 X** vs. Row 11:**10 X**	-2.146	-2.623 to -1.669	Yes
**161**	Row 1:**25 X** vs. Row 11:**25 X**	-2.089	-2.516 to -1.662	Yes
**162**	Row 1:**25 X** vs. 11:**0 X**	-2.888	-3.315 to -2.461	Yes
**163**	Row 1:**25 X** vs. 11:**10 X**	-2.462	-2.939 to -1.985	Yes
**164**	Row 1:**25 X** vs. 11:**25 X**	-2.393	-2.820 to -1.966	Yes
**165**	Row 1:**25 X** vs. Row 13:**0 X**	-2.857	-3.284 to -2.430	Yes
**166**	Row 1:**25 X** vs. Row 13:**10 X**	-2.490	-2.967 to -2.013	Yes
**167**	Row 1:**25 X** vs. Row 13:**25 X**	-2.499	-2.926 to -2.072	Yes
**168**	Row 1:**25 X** vs. Row 14:**0 X**	-2.907	-3.334 to -2.480	Yes
**169**	Row 1:**25 X** vs. Row 14:**10 X**	-2.683	-3.110 to -2.256	Yes
**170**	Row 1:**25 X** vs. Row 14:**25 X**	-2.643	-3.070 to -2.216	Yes
**171**	Row 1:**25 X** vs. 14:**0 X**	-3.501	-3.928 to -3.074	Yes
**172**	Row 1:**25 X** vs. 14:**10 X**	-2.898	-3.375 to -2.421	Yes
**173**	Row 1:**25 X** vs. 14:**25 X**	-2.534	-3.011 to -2.057	Yes
**174**	Row 1:**25 X** vs. Row 16:**0 X**	-3.614	-4.091 to -3.137	Yes
**175**	Row 1:**25 X** vs. Row 16:**10 X**	-2.982	-3.459 to -2.505	Yes
**176**	Row 1:**25 X** vs. Row 16:**25 X**	-2.127	-2.604 to -1.650	Yes
**177**	Row 1:**25 X** vs. Row 17:**0 X**	-3.240	-3.717 to -2.762	Yes
**178**	Row 1:**25 X** vs. Row 17:**10 X**	-3.000	-3.477 to -2.522	Yes
**179**	Row 1:**25 X** vs. Row 17:**25 X**	-2.293	-2.770 to -1.816	Yes
**180**	Row 1:**25 X** vs. 17:**0 X**	-3.501	-3.978 to -3.023	Yes

	2way ANOVA<br>Multiple comparisons	A<br>Data Set-A<br>Y	B<br>Data Set-B<br>Y	C<br>Data Set-C<br>Y
**181**	Row 1:**25 X** vs. 17:**10 X**	-3.294	-3.771 to -2.817	Yes
**182**	Row 1:**25 X** vs. 17:**25 X**	-2.203	-2.630 to -1.776	Yes
**183**	Row 1:**25 X** vs. Row 19:**0 X**	-3.597	-4.074 to -3.119	Yes
**184**	Row 1:**25 X** vs. Row 19:**10 X**	-3.297	-3.774 to -2.819	Yes
**185**	Row 1:**25 X** vs. Row 19:**25 X**	-2.133	-2.560 to -1.706	Yes
**186**	Row 1:**25 X** vs. Row 20:**0 X**	-3.649	-4.126 to -3.172	Yes
**187**	Row 1:**25 X** vs. Row 20:**10 X**	-3.190	-3.667 to -2.713	Yes
**188**	Row 1:**25 X** vs. Row 20:**25 X**	-2.077	-2.504 to -1.650	Yes
**189**	Row 1:**25 X** vs. Row 21:**0 X**	-3.006	-3.483 to -2.529	Yes
**190**	Row 1:**25 X** vs. Row 21:**10 X**	-2.576	-3.053 to -2.098	Yes
**191**	Row 1:**25 X** vs. Row 21:**25 X**	-2.104	-2.531 to -1.677	Yes
**192**	Row 2:**0 X** vs. Row 2:**10 X**	-0.003667	-0.4306 to 0.4232	No
**193**	Row 2:**0 X** vs. Row 2:**25 X**	-0.001667	-0.4286 to 0.4252	No
**194**	Row 2:**0 X** vs. Row 3:**0 X**	-0.01567	-0.4426 to 0.4112	No
**195**	Row 2:**0 X** vs. Row 3:**10 X**	-0.01567	-0.4426 to 0.4112	No
**196**	Row 2:**0 X** vs. Row 3:**25 X**	-0.01333	-0.4402 to 0.4136	No
**197**	Row 2:**0 X** vs. Row 4:**0 X**	-0.0510	-0.4779 to 0.3759	No
**198**	Row 2:**0 X** vs. Row 4:**10 X**	-0.04467	-0.4716 to 0.3822	No
**199**	Row 2:**0 X** vs. Row 4:**25 X**	-0.05733	-0.4842 to 0.3696	No
**200**	Row 2:**0 X** vs. Row 5:**0 X**	-0.3157	-0.7426 to 0.1112	No
**201**	Row 2:**0 X** vs. Row 5:**10 X**	-0.2830	-0.7099 to 0.1439	No
**202**	Row 2:**0 X** vs. Row 5:**25 X**	-0.2610	-0.6879 to 0.1659	No
**203**	Row 2:**0 X** vs. Row 6:**0 X**	-0.8597	-1.287 to -0.4328	Yes
**204**	Row 2:**0 X** vs. Row 6:**10 X**	-0.7550	-1.182 to -0.3281	Yes
**205**	Row 2:**0 X** vs. Row 6:**25 X**	-0.7267	-1.154 to -0.2998	Yes
**206**	Row 2:**0 X** vs. Row 7:**0 X**	-1.239	-1.666 to -0.8121	Yes
**207**	Row 2:**0 X** vs. Row 7:**10 X**	-1.162	-1.589 to -0.7348	Yes
**208**	Row 2:**0 X** vs. Row 7:**25 X**	-1.039	-1.466 to -0.6121	Yes
**209**	Row 2:**0 X** vs. Row 8:**0 X**	-1.470	-1.897 to -1.043	Yes
**210**	Row 2:**0 X** vs. Row 8:**10 X**	-1.574	-2.001 to -1.147	Yes
**211**	Row 2:**0 X** vs. Row 8:**25 X**	-1.517	-1.944 to -1.090	Yes
**212**	Row 2:**0 X** vs. Row 9:**0 X**	-1.825	-2.252 to -1.398	Yes
**213**	Row 2:**0 X** vs. Row 9:**10 X**	-1.809	-2.236 to -1.382	Yes
**214**	Row 2:**0 X** vs. Row 9:**25 X**	-1.625	-2.052 to -1.198	Yes
**215**	Row 2:**0 X** vs. Row 10:**0 X**	-2.073	-2.500 to -1.646	Yes
**216**	Row 2:**0 X** vs. Row 10:**10 X**	-2.065	-2.492 to -1.638	Yes
**217**	Row 2:**0 X** vs. Row 10:**25 X**	-1.898	-2.325 to -1.471	Yes
**218**	Row 2:**0 X** vs. Row 11:**0 X**	-2.831	-3.258 to -2.404	Yes
**219**	Row 2:**0 X** vs. Row 11:**10 X**	-2.183	-2.660 to -1.706	Yes
**220**	Row 2:**0 X** vs. Row 11:**25 X**	-2.126	-2.553 to -1.699	Yes
**221**	Row 2:**0 X** vs. 11:**0 X**	-2.925	-3.352 to -2.498	Yes
**222**	Row 2:**0 X** vs. 11:**10 X**	-2.499	-2.976 to -2.022	Yes
**223**	Row 2:**0 X** vs. 11:**25 X**	-2.430	-2.857 to -2.003	Yes
**224**	Row 2:**0 X** vs. Row 13:**0 X**	-2.894	-3.321 to -2.467	Yes
**225**	Row 2:**0 X** vs. Row 13:**10 X**	-2.527	-3.004 to -2.050	Yes

	2way ANOVA<br>Multiple comparisons	A<br>Data Set-A<br>Y	B<br>Data Set-B<br>Y	C<br>Data Set-C<br>Y
226	Row 2:**0 X** vs. Row 13:**25 X**	-2.536	-2.963 to -2.109	Yes
227	Row 2:**0 X** vs. Row 14:**0 X**	-2.944	-3.371 to -2.517	Yes
228	Row 2:**0 X** vs. Row 14:**10 X**	-2.720	-3.147 to -2.293	Yes
229	Row 2:**0 X** vs. Row 14:**25 X**	-2.680	-3.107 to -2.253	Yes
230	Row 2:**0 X** vs. 14:**0 X**	-3.538	-3.965 to -3.111	Yes
231	Row 2:**0 X** vs. 14:**10 X**	-2.935	-3.412 to -2.458	Yes
232	Row 2:**0 X** vs. 14:**25 X**	-2.571	-3.048 to -2.094	Yes
233	Row 2:**0 X** vs. Row 16:**0 X**	-3.651	-4.128 to -3.174	Yes
234	Row 2:**0 X** vs. Row 16:**10 X**	-3.019	-3.496 to -2.542	Yes
235	Row 2:**0 X** vs. Row 16:**25 X**	-2.164	-2.641 to -1.687	Yes
236	Row 2:**0 X** vs. Row 17:**0 X**	-3.277	-3.754 to -2.799	Yes
237	Row 2:**0 X** vs. Row 17:**10 X**	-3.037	-3.514 to -2.559	Yes
238	Row 2:**0 X** vs. Row 17:**25 X**	-2.330	-2.807 to -1.853	Yes
239	Row 2:**0 X** vs. 17:**0 X**	-3.538	-4.015 to -3.060	Yes
240	Row 2:**0 X** vs. 17:**10 X**	-3.331	-3.808 to -2.854	Yes
241	Row 2:**0 X** vs. 17:**25 X**	-2.240	-2.667 to -1.813	Yes
242	Row 2:**0 X** vs. Row 19:**0 X**	-3.634	-4.111 to -3.156	Yes
243	Row 2:**0 X** vs. Row 19:**10 X**	-3.334	-3.811 to -2.856	Yes
244	Row 2:**0 X** vs. Row 19:**25 X**	-2.170	-2.597 to -1.743	Yes
245	Row 2:**0 X** vs. Row 20:**0 X**	-3.686	-4.163 to -3.209	Yes
246	Row 2:**0 X** vs. Row 20:**10 X**	-3.227	-3.704 to -2.750	Yes
247	Row 2:**0 X** vs. Row 20:**25 X**	-2.114	-2.541 to -1.687	Yes
248	Row 2:**0 X** vs. Row 21:**0 X**	-3.043	-3.520 to -2.566	Yes
249	Row 2:**0 X** vs. Row 21:**10 X**	-2.613	-3.090 to -2.135	Yes
250	Row 2:**0 X** vs. Row 21:**25 X**	-2.141	-2.568 to -1.714	Yes
251	Row 2:**10 X** vs. Row 2:**25 X**	0.0020	-0.4249 to 0.4289	No
252	Row 2:**10 X** vs. Row 3:**0 X**	-0.0120	-0.4389 to 0.4149	No
253	Row 2:**10 X** vs. Row 3:**10 X**	-0.0120	-0.4389 to 0.4149	No
254	Row 2:**10 X** vs. Row 3:**25 X**	-0.009667	-0.4366 to 0.4172	No
255	Row 2:**10 X** vs. Row 4:**0 X**	-0.04733	-0.4742 to 0.3796	No
256	Row 2:**10 X** vs. Row 4:**10 X**	-0.0410	-0.4679 to 0.3859	No
257	Row 2:**10 X** vs. Row 4:**25 X**	-0.05367	-0.4806 to 0.3732	No
258	Row 2:**10 X** vs. Row 5:**0 X**	-0.3120	-0.7389 to 0.1149	No
259	Row 2:**10 X** vs. Row 5:**10 X**	-0.2793	-0.7062 to 0.1476	No
260	Row 2:**10 X** vs. Row 5:**25 X**	-0.2573	-0.6842 to 0.1696	No
261	Row 2:**10 X** vs. Row 6:**0 X**	-0.8560	-1.283 to -0.4291	Yes
262	Row 2:**10 X** vs. Row 6:**10 X**	-0.7513	-1.178 to -0.3244	Yes
263	Row 2:**10 X** vs. Row 6:**25 X**	-0.7230	-1.150 to -0.2961	Yes
264	Row 2:**10 X** vs. Row 7:**0 X**	-1.235	-1.662 to -0.8084	Yes
265	Row 2:**10 X** vs. Row 7:**10 X**	-1.158	-1.585 to -0.7311	Yes
266	Row 2:**10 X** vs. Row 7:**25 X**	-1.035	-1.462 to -0.6084	Yes
267	Row 2:**10 X** vs. Row 8:**0 X**	-1.467	-1.894 to -1.040	Yes
268	Row 2:**10 X** vs. Row 8:**10 X**	-1.570	-1.997 to -1.143	Yes
269	Row 2:**10 X** vs. Row 8:**25 X**	-1.513	-1.940 to -1.086	Yes
270	Row 2:**10 X** vs. Row 9:**0 X**	-1.821	-2.248 to -1.394	Yes

2way ANOVA<br>Multiple comparisons		A<br>Data Set-A<br>Y	B<br>Data Set-B<br>Y	C<br>Data Set-C<br>Y
**271**	Row 2:**10 X** vs. Row 9:**10 X**	-1.805	-2.232 to -1.378	Yes
**272**	Row 2:**10 X** vs. Row 9:**25 X**	-1.622	-2.049 to -1.195	Yes
**273**	Row 2:**10 X** vs. Row 10:**0 X**	-2.069	-2.496 to -1.642	Yes
**274**	Row 2:**10 X** vs. Row 10:**10 X**	-2.061	-2.488 to -1.634	Yes
**275**	Row 2:**10 X** vs. Row 10:**25 X**	-1.894	-2.321 to -1.467	Yes
**276**	Row 2:**10 X** vs. Row 11:**0 X**	-2.827	-3.254 to -2.400	Yes
**277**	Row 2:**10 X** vs. Row 11:**10 X**	-2.179	-2.657 to -1.702	Yes
**278**	Row 2:**10 X** vs. Row 11:**25 X**	-2.122	-2.549 to -1.695	Yes
**279**	Row 2:**10 X** vs. 11:**0 X**	-2.921	-3.348 to -2.494	Yes
**280**	Row 2:**10 X** vs. 11:**10 X**	-2.495	-2.973 to -2.018	Yes
**281**	Row 2:**10 X** vs. 11:**25 X**	-2.426	-2.853 to -1.999	Yes
**282**	Row 2:**10 X** vs. Row 13:**0 X**	-2.890	-3.317 to -2.463	Yes
**283**	Row 2:**10 X** vs. Row 13:**10 X**	-2.523	-3.001 to -2.046	Yes
**284**	Row 2:**10 X** vs. Row 13:**25 X**	-2.532	-2.959 to -2.105	Yes
**285**	Row 2:**10 X** vs. Row 14:**0 X**	-2.941	-3.368 to -2.514	Yes
**286**	Row 2:**10 X** vs. Row 14:**10 X**	-2.717	-3.144 to -2.290	Yes
**287**	Row 2:**10 X** vs. Row 14:**25 X**	-2.677	-3.104 to -2.250	Yes
**288**	Row 2:**10 X** vs. 14:**0 X**	-3.535	-3.962 to -3.108	Yes
**289**	Row 2:**10 X** vs. 14:**10 X**	-2.931	-3.409 to -2.454	Yes
**290**	Row 2:**10 X** vs. 14:**25 X**	-2.567	-3.045 to -2.090	Yes
**291**	Row 2:**10 X** vs. Row 16:**0 X**	-3.647	-4.125 to -3.170	Yes
**292**	Row 2:**10 X** vs. Row 16:**10 X**	-3.015	-3.493 to -2.538	Yes
**293**	Row 2:**10 X** vs. Row 16:**25 X**	-2.160	-2.638 to -1.683	Yes
**294**	Row 2:**10 X** vs. Row 17:**0 X**	-3.273	-3.750 to -2.796	Yes
**295**	Row 2:**10 X** vs. Row 17:**10 X**	-3.033	-3.510 to -2.556	Yes
**296**	Row 2:**10 X** vs. Row 17:**25 X**	-2.326	-2.804 to -1.849	Yes
**297**	Row 2:**10 X** vs. 17:**0 X**	-3.534	-4.011 to -3.057	Yes
**298**	Row 2:**10 X** vs. 17:**10 X**	-3.327	-3.805 to -2.850	Yes
**299**	Row 2:**10 X** vs. 17:**25 X**	-2.237	-2.664 to -1.810	Yes
**300**	Row 2:**10 X** vs. Row 19:**0 X**	-3.630	-4.107 to -3.153	Yes
**301**	Row 2:**10 X** vs. Row 19:**10 X**	-3.330	-3.807 to -2.853	Yes
**302**	Row 2:**10 X** vs. Row 19:**25 X**	-2.166	-2.593 to -1.739	Yes
**303**	Row 2:**10 X** vs. Row 20:**0 X**	-3.682	-4.160 to -3.205	Yes
**304**	Row 2:**10 X** vs. Row 20:**10 X**	-3.223	-3.701 to -2.746	Yes
**305**	Row 2:**10 X** vs. Row 20:**25 X**	-2.110	-2.537 to -1.683	Yes
**306**	Row 2:**10 X** vs. Row 21:**0 X**	-3.039	-3.517 to -2.562	Yes
**307**	Row 2:**10 X** vs. Row 21:**10 X**	-2.609	-3.086 to -2.132	Yes
**308**	Row 2:**10 X** vs. Row 21:**25 X**	-2.137	-2.564 to -1.710	Yes
**309**	Row 2:**25 X** vs. Row 3:**0 X**	-0.0140	-0.4409 to 0.4129	No
**310**	Row 2:**25 X** vs. Row 3:**10 X**	-0.0140	-0.4409 to 0.4129	No
**311**	Row 2:**25 X** vs. Row 3:**25 X**	-0.01167	-0.4386 to 0.4152	No
**312**	Row 2:**25 X** vs. Row 4:**0 X**	-0.04933	-0.4762 to 0.3776	No
**313**	Row 2:**25 X** vs. Row 4:**10 X**	-0.0430	-0.4699 to 0.3839	No
**314**	Row 2:**25 X** vs. Row 4:**25 X**	-0.05567	-0.4826 to 0.3712	No
**315**	Row 2:**25 X** vs. Row 5:**0 X**	-0.3140	-0.7409 to 0.1129	No

	**2way ANOVA** Multiple comparisons	**A**	**B**	**C**
		Data Set-A	Data Set-B	Data Set-C
		**Y**	**Y**	**Y**
**316**	Row 2:**25 X** vs. Row 5:**10 X**	-0.2813	-0.7082 to 0.1456	No
**317**	Row 2:**25 X** vs. Row 5:**25 X**	-0.2593	-0.6862 to 0.1676	No
**318**	Row 2:**25 X** vs. Row 6:**0 X**	-0.8580	-1.285 to -0.4311	Yes
**319**	Row 2:**25 X** vs. Row 6:**10 X**	-0.7533	-1.180 to -0.3264	Yes
**320**	Row 2:**25 X** vs. Row 6:**25 X**	-0.7250	-1.152 to -0.2981	Yes
**321**	Row 2:**25 X** vs. Row 7:**0 X**	-1.237	-1.664 to -0.8104	Yes
**322**	Row 2:**25 X** vs. Row 7:**10 X**	-1.160	-1.587 to -0.7331	Yes
**323**	Row 2:**25 X** vs. Row 7:**25 X**	-1.037	-1.464 to -0.6104	Yes
**324**	Row 2:**25 X** vs. Row 8:**0 X**	-1.469	-1.896 to -1.042	Yes
**325**	Row 2:**25 X** vs. Row 8:**10 X**	-1.572	-1.999 to -1.145	Yes
**326**	Row 2:**25 X** vs. Row 8:**25 X**	-1.515	-1.942 to -1.088	Yes
**327**	Row 2:**25 X** vs. Row 9:**0 X**	-1.823	-2.250 to -1.396	Yes
**328**	Row 2:**25 X** vs. Row 9:**10 X**	-1.807	-2.234 to -1.380	Yes
**329**	Row 2:**25 X** vs. Row 9:**25 X**	-1.624	-2.051 to -1.197	Yes
**330**	Row 2:**25 X** vs. Row 10:**0 X**	-2.071	-2.498 to -1.644	Yes
**331**	Row 2:**25 X** vs. Row 10:**10 X**	-2.063	-2.490 to -1.636	Yes
**332**	Row 2:**25 X** vs. Row 10:**25 X**	-1.896	-2.323 to -1.469	Yes
**333**	Row 2:**25 X** vs. Row 11:**0 X**	-2.829	-3.256 to -2.402	Yes
**334**	Row 2:**25 X** vs. Row 11:**10 X**	-2.181	-2.659 to -1.704	Yes
**335**	Row 2:**25 X** vs. Row 11:**25 X**	-2.124	-2.551 to -1.697	Yes
**336**	Row 2:**25 X** vs. 11:**0 X**	-2.923	-3.350 to -2.496	Yes
**337**	Row 2:**25 X** vs. 11:**10 X**	-2.497	-2.975 to -2.020	Yes
**338**	Row 2:**25 X** vs. 11:**25 X**	-2.428	-2.855 to -2.001	Yes
**339**	Row 2:**25 X** vs. Row 13:**0 X**	-2.892	-3.319 to -2.465	Yes
**340**	Row 2:**25 X** vs. Row 13:**10 X**	-2.525	-3.003 to -2.048	Yes
**341**	Row 2:**25 X** vs. Row 13:**25 X**	-2.534	-2.961 to -2.107	Yes
**342**	Row 2:**25 X** vs. Row 14:**0 X**	-2.943	-3.370 to -2.516	Yes
**343**	Row 2:**25 X** vs. Row 14:**10 X**	-2.719	-3.146 to -2.292	Yes
**344**	Row 2:**25 X** vs. Row 14:**25 X**	-2.679	-3.106 to -2.252	Yes
**345**	Row 2:**25 X** vs. 14:**0 X**	-3.537	-3.964 to -3.110	Yes
**346**	Row 2:**25 X** vs. 14:**10 X**	-2.933	-3.411 to -2.456	Yes
**347**	Row 2:**25 X** vs. 14:**25 X**	-2.569	-3.047 to -2.092	Yes
**348**	Row 2:**25 X** vs. Row 16:**0 X**	-3.649	-4.127 to -3.172	Yes
**349**	Row 2:**25 X** vs. Row 16:**10 X**	-3.017	-3.495 to -2.540	Yes
**350**	Row 2:**25 X** vs. Row 16:**25 X**	-2.162	-2.640 to -1.685	Yes
**351**	Row 2:**25 X** vs. Row 17:**0 X**	-3.275	-3.752 to -2.798	Yes
**352**	Row 2:**25 X** vs. Row 17:**10 X**	-3.035	-3.512 to -2.558	Yes
**353**	Row 2:**25 X** vs. Row 17:**25 X**	-2.328	-2.806 to -1.851	Yes
**354**	Row 2:**25 X** vs. 17:**0 X**	-3.536	-4.013 to -3.059	Yes
**355**	Row 2:**25 X** vs. 17:**10 X**	-3.329	-3.807 to -2.852	Yes
**356**	Row 2:**25 X** vs. 17:**25 X**	-2.239	-2.666 to -1.812	Yes
**357**	Row 2:**25 X** vs. Row 19:**0 X**	-3.632	-4.109 to -3.155	Yes
**358**	Row 2:**25 X** vs. Row 19:**10 X**	-3.332	-3.809 to -2.855	Yes
**359**	Row 2:**25 X** vs. Row 19:**25 X**	-2.168	-2.595 to -1.741	Yes
**360**	Row 2:**25 X** vs. Row 20:**0 X**	-3.684	-4.162 to -3.207	Yes

	2way ANOVA<br>Multiple comparisons	A<br>Data Set-A<br>Y	B<br>Data Set-B<br>Y	C<br>Data Set-C<br>Y
361	Row 2:**25 X** vs. Row 20:**10 X**	-3.225	-3.703 to -2.748	Yes
362	Row 2:**25 X** vs. Row 20:**25 X**	-2.112	-2.539 to -1.685	Yes
363	Row 2:**25 X** vs. Row 21:**0 X**	-3.041	-3.519 to -2.564	Yes
364	Row 2:**25 X** vs. Row 21:**10 X**	-2.611	-3.088 to -2.134	Yes
365	Row 2:**25 X** vs. Row 21:**25 X**	-2.139	-2.566 to -1.712	Yes
366	Row 3:**0 X** vs. Row 3:**10 X**	0.0	-0.4269 to 0.4269	No
367	Row 3:**0 X** vs. Row 3:**25 X**	0.002333	-0.4246 to 0.4292	No
368	Row 3:**0 X** vs. Row 4:**0 X**	-0.03533	-0.4622 to 0.3916	No
369	Row 3:**0 X** vs. Row 4:**10 X**	-0.0290	-0.4559 to 0.3979	No
370	Row 3:**0 X** vs. Row 4:**25 X**	-0.04167	-0.4686 to 0.3852	No
371	Row 3:**0 X** vs. Row 5:**0 X**	-0.3000	-0.7269 to 0.1269	No
372	Row 3:**0 X** vs. Row 5:**10 X**	-0.2673	-0.6942 to 0.1596	No
373	Row 3:**0 X** vs. Row 5:**25 X**	-0.2453	-0.6722 to 0.1816	No
374	Row 3:**0 X** vs. Row 6:**0 X**	-0.8440	-1.271 to -0.4171	Yes
375	Row 3:**0 X** vs. Row 6:**10 X**	-0.7393	-1.166 to -0.3124	Yes
376	Row 3:**0 X** vs. Row 6:**25 X**	-0.7110	-1.138 to -0.2841	Yes
377	Row 3:**0 X** vs. Row 7:**0 X**	-1.223	-1.650 to -0.7964	Yes
378	Row 3:**0 X** vs. Row 7:**10 X**	-1.146	-1.573 to -0.7191	Yes
379	Row 3:**0 X** vs. Row 7:**25 X**	-1.023	-1.450 to -0.5964	Yes
380	Row 3:**0 X** vs. Row 8:**0 X**	-1.455	-1.882 to -1.028	Yes
381	Row 3:**0 X** vs. Row 8:**10 X**	-1.558	-1.985 to -1.131	Yes
382	Row 3:**0 X** vs. Row 8:**25 X**	-1.501	-1.928 to -1.074	Yes
383	Row 3:**0 X** vs. Row 9:**0 X**	-1.809	-2.236 to -1.382	Yes
384	Row 3:**0 X** vs. Row 9:**10 X**	-1.793	-2.220 to -1.366	Yes
385	Row 3:**0 X** vs. Row 9:**25 X**	-1.610	-2.037 to -1.183	Yes
386	Row 3:**0 X** vs. Row 10:**0 X**	-2.057	-2.484 to -1.630	Yes
387	Row 3:**0 X** vs. Row 10:**10 X**	-2.049	-2.476 to -1.622	Yes
388	Row 3:**0 X** vs. Row 10:**25 X**	-1.882	-2.309 to -1.455	Yes
389	Row 3:**0 X** vs. Row 11:**0 X**	-2.815	-3.242 to -2.388	Yes
390	Row 3:**0 X** vs. Row 11:**10 X**	-2.167	-2.645 to -1.690	Yes
391	Row 3:**0 X** vs. Row 11:**25 X**	-2.110	-2.537 to -1.683	Yes
392	Row 3:**0 X** vs. 11:**0 X**	-2.909	-3.336 to -2.482	Yes
393	Row 3:**0 X** vs. 11:**10 X**	-2.483	-2.961 to -2.006	Yes
394	Row 3:**0 X** vs. 11:**25 X**	-2.414	-2.841 to -1.987	Yes
395	Row 3:**0 X** vs. Row 13:**0 X**	-2.878	-3.305 to -2.451	Yes
396	Row 3:**0 X** vs. Row 13:**10 X**	-2.511	-2.989 to -2.034	Yes
397	Row 3:**0 X** vs. Row 13:**25 X**	-2.520	-2.947 to -2.093	Yes
398	Row 3:**0 X** vs. Row 14:**0 X**	-2.929	-3.356 to -2.502	Yes
399	Row 3:**0 X** vs. Row 14:**10 X**	-2.705	-3.132 to -2.278	Yes
400	Row 3:**0 X** vs. Row 14:**25 X**	-2.665	-3.092 to -2.238	Yes
401	Row 3:**0 X** vs. 14:**0 X**	-3.523	-3.950 to -3.096	Yes
402	Row 3:**0 X** vs. 14:**10 X**	-2.919	-3.397 to -2.442	Yes
403	Row 3:**0 X** vs. 14:**25 X**	-2.555	-3.033 to -2.078	Yes
404	Row 3:**0 X** vs. Row 16:**0 X**	-3.635	-4.113 to -3.158	Yes
405	Row 3:**0 X** vs. Row 16:**10 X**	-3.003	-3.481 to -2.526	Yes

	**2way ANOVA** Multiple comparisons	**A** Data Set-A **Y**	**B** Data Set-B **Y**	**C** Data Set-C **Y**
**406**	Row 3:**0 X** vs. Row 16:**25 X**	-2.148	-2.626 to -1.671	Yes
**407**	Row 3:**0 X** vs. Row 17:**0 X**	-3.261	-3.738 to -2.784	Yes
**408**	Row 3:**0 X** vs. Row 17:**10 X**	-3.021	-3.498 to -2.544	Yes
**409**	Row 3:**0 X** vs. Row 17:**25 X**	-2.314	-2.792 to -1.837	Yes
**410**	Row 3:**0 X** vs. 17:**0 X**	-3.522	-3.999 to -3.045	Yes
**411**	Row 3:**0 X** vs. 17:**10 X**	-3.315	-3.793 to -2.838	Yes
**412**	Row 3:**0 X** vs. 17:**25 X**	-2.225	-2.652 to -1.798	Yes
**413**	Row 3:**0 X** vs. Row 19:**0 X**	-3.618	-4.095 to -3.141	Yes
**414**	Row 3:**0 X** vs. Row 19:**10 X**	-3.318	-3.795 to -2.841	Yes
**415**	Row 3:**0 X** vs. Row 19:**25 X**	-2.154	-2.581 to -1.727	Yes
**416**	Row 3:**0 X** vs. Row 20:**0 X**	-3.670	-4.148 to -3.193	Yes
**417**	Row 3:**0 X** vs. Row 20:**10 X**	-3.211	-3.689 to -2.734	Yes
**418**	Row 3:**0 X** vs. Row 20:**25 X**	-2.098	-2.525 to -1.671	Yes
**419**	Row 3:**0 X** vs. Row 21:**0 X**	-3.027	-3.505 to -2.550	Yes
**420**	Row 3:**0 X** vs. Row 21:**10 X**	-2.597	-3.074 to -2.120	Yes
**421**	Row 3:**0 X** vs. Row 21:**25 X**	-2.125	-2.552 to -1.698	Yes
**422**	Row 3:**10 X** vs. Row 3:**25 X**	0.002333	-0.4246 to 0.4292	No
**423**	Row 3:**10 X** vs. Row 4:**0 X**	-0.03533	-0.4622 to 0.3916	No
**424**	Row 3:**10 X** vs. Row 4:**10 X**	-0.0290	-0.4559 to 0.3979	No
**425**	Row 3:**10 X** vs. Row 4:**25 X**	-0.04167	-0.4686 to 0.3852	No
**426**	Row 3:**10 X** vs. Row 5:**0 X**	-0.3000	-0.7269 to 0.1269	No
**427**	Row 3:**10 X** vs. Row 5:**10 X**	-0.2673	-0.6942 to 0.1596	No
**428**	Row 3:**10 X** vs. Row 5:**25 X**	-0.2453	-0.6722 to 0.1816	No
**429**	Row 3:**10 X** vs. Row 6:**0 X**	-0.8440	-1.271 to -0.4171	Yes
**430**	Row 3:**10 X** vs. Row 6:**10 X**	-0.7393	-1.166 to -0.3124	Yes
**431**	Row 3:**10 X** vs. Row 6:**25 X**	-0.7110	-1.138 to -0.2841	Yes
**432**	Row 3:**10 X** vs. Row 7:**0 X**	-1.223	-1.650 to -0.7964	Yes
**433**	Row 3:**10 X** vs. Row 7:**10 X**	-1.146	-1.573 to -0.7191	Yes
**434**	Row 3:**10 X** vs. Row 7:**25 X**	-1.023	-1.450 to -0.5964	Yes
**435**	Row 3:**10 X** vs. Row 8:**0 X**	-1.455	-1.882 to -1.028	Yes
**436**	Row 3:**10 X** vs. Row 8:**10 X**	-1.558	-1.985 to -1.131	Yes
**437**	Row 3:**10 X** vs. Row 8:**25 X**	-1.501	-1.928 to -1.074	Yes
**438**	Row 3:**10 X** vs. Row 9:**0 X**	-1.809	-2.236 to -1.382	Yes
**439**	Row 3:**10 X** vs. Row 9:**10 X**	-1.793	-2.220 to -1.366	Yes
**440**	Row 3:**10 X** vs. Row 9:**25 X**	-1.610	-2.037 to -1.183	Yes
**441**	Row 3:**10 X** vs. Row 10:**0 X**	-2.057	-2.484 to -1.630	Yes
**442**	Row 3:**10 X** vs. Row 10:**10 X**	-2.049	-2.476 to -1.622	Yes
**443**	Row 3:**10 X** vs. Row 10:**25 X**	-1.882	-2.309 to -1.455	Yes
**444**	Row 3:**10 X** vs. Row 11:**0 X**	-2.815	-3.242 to -2.388	Yes
**445**	Row 3:**10 X** vs. Row 11:**10 X**	-2.167	-2.645 to -1.690	Yes
**446**	Row 3:**10 X** vs. Row 11:**25 X**	-2.110	-2.537 to -1.683	Yes
**447**	Row 3:**10 X** vs. 11:**0 X**	-2.909	-3.336 to -2.482	Yes
**448**	Row 3:**10 X** vs. 11:**10 X**	-2.483	-2.961 to -2.006	Yes
**449**	Row 3:**10 X** vs. 11:**25 X**	-2.414	-2.841 to -1.987	Yes
**450**	Row 3:**10 X** vs. Row 13:**0 X**	-2.878	-3.305 to -2.451	Yes

	2way ANOVA<br>Multiple comparisons	A<br>Data Set-A<br>Y	B<br>Data Set-B<br>Y	C<br>Data Set-C<br>Y
**451**	Row 3:**10 X** vs. Row 13:**10 X**	-2.511	-2.989 to -2.034	Yes
**452**	Row 3:**10 X** vs. Row 13:**25 X**	-2.520	-2.947 to -2.093	Yes
**453**	Row 3:**10 X** vs. Row 14:**0 X**	-2.929	-3.356 to -2.502	Yes
**454**	Row 3:**10 X** vs. Row 14:**10 X**	-2.705	-3.132 to -2.278	Yes
**455**	Row 3:**10 X** vs. Row 14:**25 X**	-2.665	-3.092 to -2.238	Yes
**456**	Row 3:**10 X** vs. 14:**0 X**	-3.523	-3.950 to -3.096	Yes
**457**	Row 3:**10 X** vs. 14:**10 X**	-2.919	-3.397 to -2.442	Yes
**458**	Row 3:**10 X** vs. 14:**25 X**	-2.555	-3.033 to -2.078	Yes
**459**	Row 3:**10 X** vs. Row 16:**0 X**	-3.635	-4.113 to -3.158	Yes
**460**	Row 3:**10 X** vs. Row 16:**10 X**	-3.003	-3.481 to -2.526	Yes
**461**	Row 3:**10 X** vs. Row 16:**25 X**	-2.148	-2.626 to -1.671	Yes
**462**	Row 3:**10 X** vs. Row 17:**0 X**	-3.261	-3.738 to -2.784	Yes
**463**	Row 3:**10 X** vs. Row 17:**10 X**	-3.021	-3.498 to -2.544	Yes
**464**	Row 3:**10 X** vs. Row 17:**25 X**	-2.314	-2.792 to -1.837	Yes
**465**	Row 3:**10 X** vs. 17:**0 X**	-3.522	-3.999 to -3.045	Yes
**466**	Row 3:**10 X** vs. 17:**10 X**	-3.315	-3.793 to -2.838	Yes
**467**	Row 3:**10 X** vs. 17:**25 X**	-2.225	-2.652 to -1.798	Yes
**468**	Row 3:**10 X** vs. Row 19:**0 X**	-3.618	-4.095 to -3.141	Yes
**469**	Row 3:**10 X** vs. Row 19:**10 X**	-3.318	-3.795 to -2.841	Yes
**470**	Row 3:**10 X** vs. Row 19:**25 X**	-2.154	-2.581 to -1.727	Yes
**471**	Row 3:**10 X** vs. Row 20:**0 X**	-3.670	-4.148 to -3.193	Yes
**472**	Row 3:**10 X** vs. Row 20:**10 X**	-3.211	-3.689 to -2.734	Yes
**473**	Row 3:**10 X** vs. Row 20:**25 X**	-2.098	-2.525 to -1.671	Yes
**474**	Row 3:**10 X** vs. Row 21:**0 X**	-3.027	-3.505 to -2.550	Yes
**475**	Row 3:**10 X** vs. Row 21:**10 X**	-2.597	-3.074 to -2.120	Yes
**476**	Row 3:**10 X** vs. Row 21:**25 X**	-2.125	-2.552 to -1.698	Yes
**477**	Row 3:**25 X** vs. Row 4:**0 X**	-0.03767	-0.4646 to 0.3892	No
**478**	Row 3:**25 X** vs. Row 4:**10 X**	-0.03133	-0.4582 to 0.3956	No
**479**	Row 3:**25 X** vs. Row 4:**25 X**	-0.0440	-0.4709 to 0.3829	No
**480**	Row 3:**25 X** vs. Row 5:**0 X**	-0.3023	-0.7292 to 0.1246	No
**481**	Row 3:**25 X** vs. Row 5:**10 X**	-0.2697	-0.6966 to 0.1572	No
**482**	Row 3:**25 X** vs. Row 5:**25 X**	-0.2477	-0.6746 to 0.1792	No
**483**	Row 3:**25 X** vs. Row 6:**0 X**	-0.8463	-1.273 to -0.4194	Yes
**484**	Row 3:**25 X** vs. Row 6:**10 X**	-0.7417	-1.169 to -0.3148	Yes
**485**	Row 3:**25 X** vs. Row 6:**25 X**	-0.7133	-1.140 to -0.2864	Yes
**486**	Row 3:**25 X** vs. Row 7:**0 X**	-1.226	-1.653 to -0.7988	Yes
**487**	Row 3:**25 X** vs. Row 7:**10 X**	-1.148	-1.575 to -0.7214	Yes
**488**	Row 3:**25 X** vs. Row 7:**25 X**	-1.026	-1.453 to -0.5988	Yes
**489**	Row 3:**25 X** vs. Row 8:**0 X**	-1.457	-1.884 to -1.030	Yes
**490**	Row 3:**25 X** vs. Row 8:**10 X**	-1.560	-1.987 to -1.133	Yes
**491**	Row 3:**25 X** vs. Row 8:**25 X**	-1.504	-1.931 to -1.077	Yes
**492**	Row 3:**25 X** vs. Row 9:**0 X**	-1.812	-2.239 to -1.385	Yes
**493**	Row 3:**25 X** vs. Row 9:**10 X**	-1.796	-2.223 to -1.369	Yes
**494**	Row 3:**25 X** vs. Row 9:**25 X**	-1.612	-2.039 to -1.185	Yes
**495**	Row 3:**25 X** vs. Row 10:**0 X**	-2.060	-2.487 to -1.633	Yes

	2way ANOVA<br>Multiple comparisons	A<br>Data Set-A<br>Y	B<br>Data Set-B<br>Y	C<br>Data Set-C<br>Y
496	Row 3:**25 X** vs. Row 10:**10 X**	-2.051	-2.478 to -1.624	Yes
497	Row 3:**25 X** vs. Row 10:**25 X**	-1.885	-2.312 to -1.458	Yes
498	Row 3:**25 X** vs. Row 11:**0 X**	-2.818	-3.245 to -2.391	Yes
499	Row 3:**25 X** vs. Row 11:**10 X**	-2.170	-2.647 to -1.692	Yes
500	Row 3:**25 X** vs. Row 11:**25 X**	-2.112	-2.539 to -1.685	Yes
501	Row 3:**25 X** vs. 11:**0 X**	-2.911	-3.338 to -2.484	Yes
502	Row 3:**25 X** vs. 11:**10 X**	-2.486	-2.963 to -2.008	Yes
503	Row 3:**25 X** vs. 11:**25 X**	-2.416	-2.843 to -1.989	Yes
504	Row 3:**25 X** vs. Row 13:**0 X**	-2.880	-3.307 to -2.453	Yes
505	Row 3:**25 X** vs. Row 13:**10 X**	-2.514	-2.991 to -2.036	Yes
506	Row 3:**25 X** vs. Row 13:**25 X**	-2.522	-2.949 to -2.095	Yes
507	Row 3:**25 X** vs. Row 14:**0 X**	-2.931	-3.358 to -2.504	Yes
508	Row 3:**25 X** vs. Row 14:**10 X**	-2.707	-3.134 to -2.280	Yes
509	Row 3:**25 X** vs. Row 14:**25 X**	-2.667	-3.094 to -2.240	Yes
510	Row 3:**25 X** vs. 14:**0 X**	-3.525	-3.952 to -3.098	Yes
511	Row 3:**25 X** vs. 14:**10 X**	-2.922	-3.399 to -2.444	Yes
512	Row 3:**25 X** vs. 14:**25 X**	-2.558	-3.035 to -2.080	Yes
513	Row 3:**25 X** vs. Row 16:**0 X**	-3.638	-4.115 to -3.160	Yes
514	Row 3:**25 X** vs. Row 16:**10 X**	-3.006	-3.483 to -2.528	Yes
515	Row 3:**25 X** vs. Row 16:**25 X**	-2.151	-2.628 to -1.673	Yes
516	Row 3:**25 X** vs. Row 17:**0 X**	-3.263	-3.740 to -2.786	Yes
517	Row 3:**25 X** vs. Row 17:**10 X**	-3.023	-3.500 to -2.546	Yes
518	Row 3:**25 X** vs. Row 17:**25 X**	-2.317	-2.794 to -1.839	Yes
519	Row 3:**25 X** vs. 17:**0 X**	-3.524	-4.001 to -3.047	Yes
520	Row 3:**25 X** vs. 17:**10 X**	-3.318	-3.795 to -2.840	Yes
521	Row 3:**25 X** vs. 17:**25 X**	-2.227	-2.654 to -1.800	Yes
522	Row 3:**25 X** vs. Row 19:**0 X**	-3.620	-4.097 to -3.143	Yes
523	Row 3:**25 X** vs. Row 19:**10 X**	-3.320	-3.797 to -2.843	Yes
524	Row 3:**25 X** vs. Row 19:**25 X**	-2.157	-2.584 to -1.730	Yes
525	Row 3:**25 X** vs. Row 20:**0 X**	-3.673	-4.150 to -3.195	Yes
526	Row 3:**25 X** vs. Row 20:**10 X**	-3.214	-3.691 to -2.736	Yes
527	Row 3:**25 X** vs. Row 20:**25 X**	-2.100	-2.527 to -1.673	Yes
528	Row 3:**25 X** vs. Row 21:**0 X**	-3.030	-3.507 to -2.552	Yes
529	Row 3:**25 X** vs. Row 21:**10 X**	-2.599	-3.076 to -2.122	Yes
530	Row 3:**25 X** vs. Row 21:**25 X**	-2.128	-2.555 to -1.701	Yes
531	Row 4:**0 X** vs. Row 4:**10 X**	0.006333	-0.4206 to 0.4332	No
532	Row 4:**0 X** vs. Row 4:**25 X**	-0.006333	-0.4332 to 0.4206	No
533	Row 4:**0 X** vs. Row 5:**0 X**	-0.2647	-0.6916 to 0.1622	No
534	Row 4:**0 X** vs. Row 5:**10 X**	-0.2320	-0.6589 to 0.1949	No
535	Row 4:**0 X** vs. Row 5:**25 X**	-0.2100	-0.6369 to 0.2169	No
536	Row 4:**0 X** vs. Row 6:**0 X**	-0.8087	-1.236 to -0.3818	Yes
537	Row 4:**0 X** vs. Row 6:**10 X**	-0.7040	-1.131 to -0.2771	Yes
538	Row 4:**0 X** vs. Row 6:**25 X**	-0.6757	-1.103 to -0.2488	Yes
539	Row 4:**0 X** vs. Row 7:**0 X**	-1.188	-1.615 to -0.7611	Yes
540	Row 4:**0 X** vs. Row 7:**10 X**	-1.111	-1.538 to -0.6838	Yes

	2way ANOVA<br>Multiple comparisons	A<br>Data Set-A<br>Y	B<br>Data Set-B<br>Y	C<br>Data Set-C<br>Y
541	Row 4:**0 X** vs. Row 7:**25 X**	-0.9880	-1.415 to -0.5611	Yes
542	Row 4:**0 X** vs. Row 8:**0 X**	-1.419	-1.846 to -0.9924	Yes
543	Row 4:**0 X** vs. Row 8:**10 X**	-1.523	-1.950 to -1.096	Yes
544	Row 4:**0 X** vs. Row 8:**25 X**	-1.466	-1.893 to -1.039	Yes
545	Row 4:**0 X** vs. Row 9:**0 X**	-1.774	-2.201 to -1.347	Yes
546	Row 4:**0 X** vs. Row 9:**10 X**	-1.758	-2.185 to -1.331	Yes
547	Row 4:**0 X** vs. Row 9:**25 X**	-1.574	-2.001 to -1.147	Yes
548	Row 4:**0 X** vs. Row 10:**0 X**	-2.022	-2.449 to -1.595	Yes
549	Row 4:**0 X** vs. Row 10:**10 X**	-2.014	-2.441 to -1.587	Yes
550	Row 4:**0 X** vs. Row 10:**25 X**	-1.847	-2.274 to -1.420	Yes
551	Row 4:**0 X** vs. Row 11:**0 X**	-2.780	-3.207 to -2.353	Yes
552	Row 4:**0 X** vs. Row 11:**10 X**	-2.132	-2.609 to -1.655	Yes
553	Row 4:**0 X** vs. Row 11:**25 X**	-2.075	-2.502 to -1.648	Yes
554	Row 4:**0 X** vs. 11:**0 X**	-2.874	-3.301 to -2.447	Yes
555	Row 4:**0 X** vs. 11:**10 X**	-2.448	-2.925 to -1.971	Yes
556	Row 4:**0 X** vs. 11:**25 X**	-2.379	-2.806 to -1.952	Yes
557	Row 4:**0 X** vs. Row 13:**0 X**	-2.843	-3.270 to -2.416	Yes
558	Row 4:**0 X** vs. Row 13:**10 X**	-2.476	-2.953 to -1.999	Yes
559	Row 4:**0 X** vs. Row 13:**25 X**	-2.485	-2.912 to -2.058	Yes
560	Row 4:**0 X** vs. Row 14:**0 X**	-2.893	-3.320 to -2.466	Yes
561	Row 4:**0 X** vs. Row 14:**10 X**	-2.669	-3.096 to -2.242	Yes
562	Row 4:**0 X** vs. Row 14:**25 X**	-2.629	-3.056 to -2.202	Yes
563	Row 4:**0 X** vs. 14:**0 X**	-3.487	-3.914 to -3.060	Yes
564	Row 4:**0 X** vs. 14:**10 X**	-2.884	-3.361 to -2.407	Yes
565	Row 4:**0 X** vs. 14:**25 X**	-2.520	-2.997 to -2.043	Yes
566	Row 4:**0 X** vs. Row 16:**0 X**	-3.600	-4.077 to -3.123	Yes
567	Row 4:**0 X** vs. Row 16:**10 X**	-2.968	-3.445 to -2.491	Yes
568	Row 4:**0 X** vs. Row 16:**25 X**	-2.113	-2.590 to -1.636	Yes
569	Row 4:**0 X** vs. Row 17:**0 X**	-3.226	-3.703 to -2.748	Yes
570	Row 4:**0 X** vs. Row 17:**10 X**	-2.986	-3.463 to -2.508	Yes
571	Row 4:**0 X** vs. Row 17:**25 X**	-2.279	-2.756 to -1.802	Yes
572	Row 4:**0 X** vs. 17:**0 X**	-3.487	-3.964 to -3.009	Yes
573	Row 4:**0 X** vs. 17:**10 X**	-3.280	-3.757 to -2.803	Yes
574	Row 4:**0 X** vs. 17:**25 X**	-2.189	-2.616 to -1.762	Yes
575	Row 4:**0 X** vs. Row 19:**0 X**	-3.583	-4.060 to -3.105	Yes
576	Row 4:**0 X** vs. Row 19:**10 X**	-3.283	-3.760 to -2.805	Yes
577	Row 4:**0 X** vs. Row 19:**25 X**	-2.119	-2.546 to -1.692	Yes
578	Row 4:**0 X** vs. Row 20:**0 X**	-3.635	-4.112 to -3.158	Yes
579	Row 4:**0 X** vs. Row 20:**10 X**	-3.176	-3.653 to -2.699	Yes
580	Row 4:**0 X** vs. Row 20:**25 X**	-2.063	-2.490 to -1.636	Yes
581	Row 4:**0 X** vs. Row 21:**0 X**	-2.992	-3.469 to -2.515	Yes
582	Row 4:**0 X** vs. Row 21:**10 X**	-2.562	-3.039 to -2.084	Yes
583	Row 4:**0 X** vs. Row 21:**25 X**	-2.090	-2.517 to -1.663	Yes
584	Row 4:**10 X** vs. Row 4:**25 X**	-0.01267	-0.4396 to 0.4142	No
585	Row 4:**10 X** vs. Row 5:**0 X**	-0.2710	-0.6979 to 0.1559	No

	2way ANOVA Multiple comparisons	A Data Set-A Y	B Data Set-B Y	C Data Set-C Y
586	Row 4:**10 X** vs. Row 5:**10 X**	-0.2383	-0.6652 to 0.1886	No
587	Row 4:**10 X** vs. Row 5:**25 X**	-0.2163	-0.6432 to 0.2106	No
588	Row 4:**10 X** vs. Row 6:**0 X**	-0.8150	-1.242 to -0.3881	Yes
589	Row 4:**10 X** vs. Row 6:**10 X**	-0.7103	-1.137 to -0.2834	Yes
590	Row 4:**10 X** vs. Row 6:**25 X**	-0.6820	-1.109 to -0.2551	Yes
591	Row 4:**10 X** vs. Row 7:**0 X**	-1.194	-1.621 to -0.7674	Yes
592	Row 4:**10 X** vs. Row 7:**10 X**	-1.117	-1.544 to -0.6901	Yes
593	Row 4:**10 X** vs. Row 7:**25 X**	-0.9943	-1.421 to -0.5674	Yes
594	Row 4:**10 X** vs. Row 8:**0 X**	-1.426	-1.853 to -0.9988	Yes
595	Row 4:**10 X** vs. Row 8:**10 X**	-1.529	-1.956 to -1.102	Yes
596	Row 4:**10 X** vs. Row 8:**25 X**	-1.472	-1.899 to -1.045	Yes
597	Row 4:**10 X** vs. Row 9:**0 X**	-1.780	-2.207 to -1.353	Yes
598	Row 4:**10 X** vs. Row 9:**10 X**	-1.764	-2.191 to -1.337	Yes
599	Row 4:**10 X** vs. Row 9:**25 X**	-1.581	-2.008 to -1.154	Yes
600	Row 4:**10 X** vs. Row 10:**0 X**	-2.028	-2.455 to -1.601	Yes
601	Row 4:**10 X** vs. Row 10:**10 X**	-2.020	-2.447 to -1.593	Yes
602	Row 4:**10 X** vs. Row 10:**25 X**	-1.853	-2.280 to -1.426	Yes
603	Row 4:**10 X** vs. Row 11:**0 X**	-2.786	-3.213 to -2.359	Yes
604	Row 4:**10 X** vs. Row 11:**10 X**	-2.138	-2.616 to -1.661	Yes
605	Row 4:**10 X** vs. Row 11:**25 X**	-2.081	-2.508 to -1.654	Yes
606	Row 4:**10 X** vs. 11:**0 X**	-2.880	-3.307 to -2.453	Yes
607	Row 4:**10 X** vs. 11:**10 X**	-2.454	-2.932 to -1.977	Yes
608	Row 4:**10 X** vs. 11:**25 X**	-2.385	-2.812 to -1.958	Yes
609	Row 4:**10 X** vs. Row 13:**0 X**	-2.849	-3.276 to -2.422	Yes
610	Row 4:**10 X** vs. Row 13:**10 X**	-2.482	-2.960 to -2.005	Yes
611	Row 4:**10 X** vs. Row 13:**25 X**	-2.491	-2.918 to -2.064	Yes
612	Row 4:**10 X** vs. Row 14:**0 X**	-2.900	-3.327 to -2.473	Yes
613	Row 4:**10 X** vs. Row 14:**10 X**	-2.676	-3.103 to -2.249	Yes
614	Row 4:**10 X** vs. Row 14:**25 X**	-2.636	-3.063 to -2.209	Yes
615	Row 4:**10 X** vs. 14:**0 X**	-3.494	-3.921 to -3.067	Yes
616	Row 4:**10 X** vs. 14:**10 X**	-2.890	-3.368 to -2.413	Yes
617	Row 4:**10 X** vs. 14:**25 X**	-2.526	-3.004 to -2.049	Yes
618	Row 4:**10 X** vs. Row 16:**0 X**	-3.606	-4.084 to -3.129	Yes
619	Row 4:**10 X** vs. Row 16:**10 X**	-2.974	-3.452 to -2.497	Yes
620	Row 4:**10 X** vs. Row 16:**25 X**	-2.119	-2.597 to -1.642	Yes
621	Row 4:**10 X** vs. Row 17:**0 X**	-3.232	-3.709 to -2.755	Yes
622	Row 4:**10 X** vs. Row 17:**10 X**	-2.992	-3.469 to -2.515	Yes
623	Row 4:**10 X** vs. Row 17:**25 X**	-2.285	-2.763 to -1.808	Yes
624	Row 4:**10 X** vs. 17:**0 X**	-3.493	-3.970 to -3.016	Yes
625	Row 4:**10 X** vs. 17:**10 X**	-3.286	-3.764 to -2.809	Yes
626	Row 4:**10 X** vs. 17:**25 X**	-2.196	-2.623 to -1.769	Yes
627	Row 4:**10 X** vs. Row 19:**0 X**	-3.589	-4.066 to -3.112	Yes
628	Row 4:**10 X** vs. Row 19:**10 X**	-3.289	-3.766 to -2.812	Yes
629	Row 4:**10 X** vs. Row 19:**25 X**	-2.125	-2.552 to -1.698	Yes
630	Row 4:**10 X** vs. Row 20:**0 X**	-3.641	-4.119 to -3.164	Yes

	2way ANOVA<br>Multiple comparisons	A<br>Data Set-A<br>Y	B<br>Data Set-B<br>Y	C<br>Data Set-C<br>Y
**631**	Row 4:**10 X** vs. Row 20:**10 X**	-3.182	-3.660 to -2.705	Yes
**632**	Row 4:**10 X** vs. Row 20:**25 X**	-2.069	-2.496 to -1.642	Yes
**633**	Row 4:**10 X** vs. Row 21:**0 X**	-2.998	-3.476 to -2.521	Yes
**634**	Row 4:**10 X** vs. Row 21:**10 X**	-2.568	-3.045 to -2.091	Yes
**635**	Row 4:**10 X** vs. Row 21:**25 X**	-2.096	-2.523 to -1.669	Yes
**636**	Row 4:**25 X** vs. Row 5:**0 X**	-0.2583	-0.6852 to 0.1686	No
**637**	Row 4:**25 X** vs. Row 5:**10 X**	-0.2257	-0.6526 to 0.2012	No
**638**	Row 4:**25 X** vs. Row 5:**25 X**	-0.2037	-0.6306 to 0.2232	No
**639**	Row 4:**25 X** vs. Row 6:**0 X**	-0.8023	-1.229 to -0.3754	Yes
**640**	Row 4:**25 X** vs. Row 6:**10 X**	-0.6977	-1.125 to -0.2708	Yes
**641**	Row 4:**25 X** vs. Row 6:**25 X**	-0.6693	-1.096 to -0.2424	Yes
**642**	Row 4:**25 X** vs. Row 7:**0 X**	-1.182	-1.609 to -0.7548	Yes
**643**	Row 4:**25 X** vs. Row 7:**10 X**	-1.104	-1.531 to -0.6774	Yes
**644**	Row 4:**25 X** vs. Row 7:**25 X**	-0.9817	-1.409 to -0.5548	Yes
**645**	Row 4:**25 X** vs. Row 8:**0 X**	-1.413	-1.840 to -0.9861	Yes
**646**	Row 4:**25 X** vs. Row 8:**10 X**	-1.516	-1.943 to -1.089	Yes
**647**	Row 4:**25 X** vs. Row 8:**25 X**	-1.460	-1.887 to -1.033	Yes
**648**	Row 4:**25 X** vs. Row 9:**0 X**	-1.768	-2.195 to -1.341	Yes
**649**	Row 4:**25 X** vs. Row 9:**10 X**	-1.752	-2.179 to -1.325	Yes
**650**	Row 4:**25 X** vs. Row 9:**25 X**	-1.568	-1.995 to -1.141	Yes
**651**	Row 4:**25 X** vs. Row 10:**0 X**	-2.016	-2.443 to -1.589	Yes
**652**	Row 4:**25 X** vs. Row 10:**10 X**	-2.007	-2.434 to -1.580	Yes
**653**	Row 4:**25 X** vs. Row 10:**25 X**	-1.841	-2.268 to -1.414	Yes
**654**	Row 4:**25 X** vs. Row 11:**0 X**	-2.774	-3.201 to -2.347	Yes
**655**	Row 4:**25 X** vs. Row 11:**10 X**	-2.126	-2.603 to -1.648	Yes
**656**	Row 4:**25 X** vs. Row 11:**25 X**	-2.068	-2.495 to -1.641	Yes
**657**	Row 4:**25 X** vs. 11:**0 X**	-2.867	-3.294 to -2.440	Yes
**658**	Row 4:**25 X** vs. 11:**10 X**	-2.442	-2.919 to -1.964	Yes
**659**	Row 4:**25 X** vs. 11:**25 X**	-2.372	-2.799 to -1.945	Yes
**660**	Row 4:**25 X** vs. Row 13:**0 X**	-2.836	-3.263 to -2.409	Yes
**661**	Row 4:**25 X** vs. Row 13:**10 X**	-2.470	-2.947 to -1.992	Yes
**662**	Row 4:**25 X** vs. Row 13:**25 X**	-2.478	-2.905 to -2.051	Yes
**663**	Row 4:**25 X** vs. Row 14:**0 X**	-2.887	-3.314 to -2.460	Yes
**664**	Row 4:**25 X** vs. Row 14:**10 X**	-2.663	-3.090 to -2.236	Yes
**665**	Row 4:**25 X** vs. Row 14:**25 X**	-2.623	-3.050 to -2.196	Yes
**666**	Row 4:**25 X** vs. 14:**0 X**	-3.481	-3.908 to -3.054	Yes
**667**	Row 4:**25 X** vs. 14:**10 X**	-2.878	-3.355 to -2.400	Yes
**668**	Row 4:**25 X** vs. 14:**25 X**	-2.514	-2.991 to -2.036	Yes
**669**	Row 4:**25 X** vs. Row 16:**0 X**	-3.594	-4.071 to -3.116	Yes
**670**	Row 4:**25 X** vs. Row 16:**10 X**	-2.962	-3.439 to -2.484	Yes
**671**	Row 4:**25 X** vs. Row 16:**25 X**	-2.107	-2.584 to -1.629	Yes
**672**	Row 4:**25 X** vs. Row 17:**0 X**	-3.219	-3.696 to -2.742	Yes
**673**	Row 4:**25 X** vs. Row 17:**10 X**	-2.979	-3.456 to -2.502	Yes
**674**	Row 4:**25 X** vs. Row 17:**25 X**	-2.273	-2.750 to -1.795	Yes
**675**	Row 4:**25 X** vs. 17:**0 X**	-3.480	-3.957 to -3.003	Yes

	**2way ANOVA** Multiple comparisons	A Data Set-A Y	B Data Set-B Y	C Data Set-C Y
**676**	Row 4:**25 X** vs. 17:**10 X**	-3.274	-3.751 to -2.796	Yes
**677**	Row 4:**25 X** vs. 17:**25 X**	-2.183	-2.610 to -1.756	Yes
**678**	Row 4:**25 X** vs. Row 19:**0 X**	-3.576	-4.053 to -3.099	Yes
**679**	Row 4:**25 X** vs. Row 19:**10 X**	-3.276	-3.753 to -2.799	Yes
**680**	Row 4:**25 X** vs. Row 19:**25 X**	-2.113	-2.540 to -1.686	Yes
**681**	Row 4:**25 X** vs. Row 20:**0 X**	-3.629	-4.106 to -3.151	Yes
**682**	Row 4:**25 X** vs. Row 20:**10 X**	-3.170	-3.647 to -2.692	Yes
**683**	Row 4:**25 X** vs. Row 20:**25 X**	-2.056	-2.483 to -1.629	Yes
**684**	Row 4:**25 X** vs. Row 21:**0 X**	-2.986	-3.463 to -2.508	Yes
**685**	Row 4:**25 X** vs. Row 21:**10 X**	-2.555	-3.032 to -2.078	Yes
**686**	Row 4:**25 X** vs. Row 21:**25 X**	-2.084	-2.511 to -1.657	Yes
**687**	Row 5:**0 X** vs. Row 5:**10 X**	0.03267	-0.3942 to 0.4596	No
**688**	Row 5:**0 X** vs. Row 5:**25 X**	0.05467	-0.3722 to 0.4816	No
**689**	Row 5:**0 X** vs. Row 6:**0 X**	-0.5440	-0.9709 to -0.1171	Yes
**690**	Row 5:**0 X** vs. Row 6:**10 X**	-0.4393	-0.8662 to -0.01242	Yes
**691**	Row 5:**0 X** vs. Row 6:**25 X**	-0.4110	-0.8379 to 0.01591	No
**692**	Row 5:**0 X** vs. Row 7:**0 X**	-0.9233	-1.350 to -0.4964	Yes
**693**	Row 5:**0 X** vs. Row 7:**10 X**	-0.8460	-1.273 to -0.4191	Yes
**694**	Row 5:**0 X** vs. Row 7:**25 X**	-0.7233	-1.150 to -0.2964	Yes
**695**	Row 5:**0 X** vs. Row 8:**0 X**	-1.155	-1.582 to -0.7278	Yes
**696**	Row 5:**0 X** vs. Row 8:**10 X**	-1.258	-1.685 to -0.8311	Yes
**697**	Row 5:**0 X** vs. Row 8:**25 X**	-1.201	-1.628 to -0.7744	Yes
**698**	Row 5:**0 X** vs. Row 9:**0 X**	-1.509	-1.936 to -1.082	Yes
**699**	Row 5:**0 X** vs. Row 9:**10 X**	-1.493	-1.920 to -1.066	Yes
**700**	Row 5:**0 X** vs. Row 9:**25 X**	-1.310	-1.737 to -0.8828	Yes
**701**	Row 5:**0 X** vs. Row 10:**0 X**	-1.757	-2.184 to -1.330	Yes
**702**	Row 5:**0 X** vs. Row 10:**10 X**	-1.749	-2.176 to -1.322	Yes
**703**	Row 5:**0 X** vs. Row 10:**25 X**	-1.582	-2.009 to -1.155	Yes
**704**	Row 5:**0 X** vs. Row 11:**0 X**	-2.515	-2.942 to -2.088	Yes
**705**	Row 5:**0 X** vs. Row 11:**10 X**	-1.867	-2.345 to -1.390	Yes
**706**	Row 5:**0 X** vs. Row 11:**25 X**	-1.810	-2.237 to -1.383	Yes
**707**	Row 5:**0 X** vs. 11:**0 X**	-2.609	-3.036 to -2.182	Yes
**708**	Row 5:**0 X** vs. 11:**10 X**	-2.183	-2.661 to -1.706	Yes
**709**	Row 5:**0 X** vs. 11:**25 X**	-2.114	-2.541 to -1.687	Yes
**710**	Row 5:**0 X** vs. Row 13:**0 X**	-2.578	-3.005 to -2.151	Yes
**711**	Row 5:**0 X** vs. Row 13:**10 X**	-2.211	-2.689 to -1.734	Yes
**712**	Row 5:**0 X** vs. Row 13:**25 X**	-2.220	-2.647 to -1.793	Yes
**713**	Row 5:**0 X** vs. Row 14:**0 X**	-2.629	-3.056 to -2.202	Yes
**714**	Row 5:**0 X** vs. Row 14:**10 X**	-2.405	-2.832 to -1.978	Yes
**715**	Row 5:**0 X** vs. Row 14:**25 X**	-2.365	-2.792 to -1.938	Yes
**716**	Row 5:**0 X** vs. 14:**0 X**	-3.223	-3.650 to -2.796	Yes
**717**	Row 5:**0 X** vs. 14:**10 X**	-2.619	-3.097 to -2.142	Yes
**718**	Row 5:**0 X** vs. 14:**25 X**	-2.255	-2.733 to -1.778	Yes
**719**	Row 5:**0 X** vs. Row 16:**0 X**	-3.335	-3.813 to -2.858	Yes
**720**	Row 5:**0 X** vs. Row 16:**10 X**	-2.703	-3.181 to -2.226	Yes

	2way ANOVA<br>Multiple comparisons	A<br>Data Set-A<br>Y	B<br>Data Set-B<br>Y	C<br>Data Set-C<br>Y
721	Row 5:**0 X** vs. Row 16:**25 X**	-1.848	-2.326 to -1.371	Yes
722	Row 5:**0 X** vs. Row 17:**0 X**	-2.961	-3.438 to -2.484	Yes
723	Row 5:**0 X** vs. Row 17:**10 X**	-2.721	-3.198 to -2.244	Yes
724	Row 5:**0 X** vs. Row 17:**25 X**	-2.014	-2.492 to -1.537	Yes
725	Row 5:**0 X** vs. 17:**0 X**	-3.222	-3.699 to -2.745	Yes
726	Row 5:**0 X** vs. 17:**10 X**	-3.015	-3.493 to -2.538	Yes
727	Row 5:**0 X** vs. 17:**25 X**	-1.925	-2.352 to -1.498	Yes
728	Row 5:**0 X** vs. Row 19:**0 X**	-3.318	-3.795 to -2.841	Yes
729	Row 5:**0 X** vs. Row 19:**10 X**	-3.018	-3.495 to -2.541	Yes
730	Row 5:**0 X** vs. Row 19:**25 X**	-1.854	-2.281 to -1.427	Yes
731	Row 5:**0 X** vs. Row 20:**0 X**	-3.370	-3.848 to -2.893	Yes
732	Row 5:**0 X** vs. Row 20:**10 X**	-2.911	-3.389 to -2.434	Yes
733	Row 5:**0 X** vs. Row 20:**25 X**	-1.798	-2.225 to -1.371	Yes
734	Row 5:**0 X** vs. Row 21:**0 X**	-2.727	-3.205 to -2.250	Yes
735	Row 5:**0 X** vs. Row 21:**10 X**	-2.297	-2.774 to -1.820	Yes
736	Row 5:**0 X** vs. Row 21:**25 X**	-1.825	-2.252 to -1.398	Yes
737	Row 5:**10 X** vs. Row 5:**25 X**	0.02200	-0.4049 to 0.4489	No
738	Row 5:**10 X** vs. Row 6:**0 X**	-0.5767	-1.004 to -0.1498	Yes
739	Row 5:**10 X** vs. Row 6:**10 X**	-0.4720	-0.8989 to -0.04509	Yes
740	Row 5:**10 X** vs. Row 6:**25 X**	-0.4437	-0.8706 to -0.01675	Yes
741	Row 5:**10 X** vs. Row 7:**0 X**	-0.9560	-1.383 to -0.5291	Yes
742	Row 5:**10 X** vs. Row 7:**10 X**	-0.8787	-1.306 to -0.4518	Yes
743	Row 5:**10 X** vs. Row 7:**25 X**	-0.7560	-1.183 to -0.3291	Yes
744	Row 5:**10 X** vs. Row 8:**0 X**	-1.187	-1.614 to -0.7604	Yes
745	Row 5:**10 X** vs. Row 8:**10 X**	-1.291	-1.718 to -0.8638	Yes
746	Row 5:**10 X** vs. Row 8:**25 X**	-1.234	-1.661 to -0.8071	Yes
747	Row 5:**10 X** vs. Row 9:**0 X**	-1.542	-1.969 to -1.115	Yes
748	Row 5:**10 X** vs. Row 9:**10 X**	-1.526	-1.953 to -1.099	Yes
749	Row 5:**10 X** vs. Row 9:**25 X**	-1.342	-1.769 to -0.9154	Yes
750	Row 5:**10 X** vs. Row 10:**0 X**	-1.790	-2.217 to -1.363	Yes
751	Row 5:**10 X** vs. Row 10:**10 X**	-1.782	-2.209 to -1.355	Yes
752	Row 5:**10 X** vs. Row 10:**25 X**	-1.615	-2.042 to -1.188	Yes
753	Row 5:**10 X** vs. Row 11:**0 X**	-2.548	-2.975 to -2.121	Yes
754	Row 5:**10 X** vs. Row 11:**10 X**	-1.900	-2.377 to -1.423	Yes
755	Row 5:**10 X** vs. Row 11:**25 X**	-1.843	-2.270 to -1.416	Yes
756	Row 5:**10 X** vs. 11:**0 X**	-2.642	-3.069 to -2.215	Yes
757	Row 5:**10 X** vs. 11:**10 X**	-2.216	-2.693 to -1.739	Yes
758	Row 5:**10 X** vs. 11:**25 X**	-2.147	-2.574 to -1.720	Yes
759	Row 5:**10 X** vs. Row 13:**0 X**	-2.611	-3.038 to -2.184	Yes
760	Row 5:**10 X** vs. Row 13:**10 X**	-2.244	-2.721 to -1.767	Yes
761	Row 5:**10 X** vs. Row 13:**25 X**	-2.253	-2.680 to -1.826	Yes
762	Row 5:**10 X** vs. Row 14:**0 X**	-2.661	-3.088 to -2.234	Yes
763	Row 5:**10 X** vs. Row 14:**10 X**	-2.437	-2.864 to -2.010	Yes
764	Row 5:**10 X** vs. Row 14:**25 X**	-2.397	-2.824 to -1.970	Yes
765	Row 5:**10 X** vs. 14:**0 X**	-3.255	-3.682 to -2.828	Yes

	**2way ANOVA** Multiple comparisons	**A** Data Set-A **Y**	**B** Data Set-B **Y**	**C** Data Set-C **Y**
**766**	Row 5:**10 X** vs. 14:**10 X**	-2.652	-3.129 to -2.175	Yes
**767**	Row 5:**10 X** vs. 14:**25 X**	-2.288	-2.765 to -1.811	Yes
**768**	Row 5:**10 X** vs. Row 16:**0 X**	-3.368	-3.845 to -2.891	Yes
**769**	Row 5:**10 X** vs. Row 16:**10 X**	-2.736	-3.213 to -2.259	Yes
**770**	Row 5:**10 X** vs. Row 16:**25 X**	-1.881	-2.358 to -1.404	Yes
**771**	Row 5:**10 X** vs. Row 17:**0 X**	-2.994	-3.471 to -2.516	Yes
**772**	Row 5:**10 X** vs. Row 17:**10 X**	-2.754	-3.231 to -2.276	Yes
**773**	Row 5:**10 X** vs. Row 17:**25 X**	-2.047	-2.524 to -1.570	Yes
**774**	Row 5:**10 X** vs. 17:**0 X**	-3.255	-3.732 to -2.777	Yes
**775**	Row 5:**10 X** vs. 17:**10 X**	-3.048	-3.525 to -2.571	Yes
**776**	Row 5:**10 X** vs. 17:**25 X**	-1.957	-2.384 to -1.530	Yes
**777**	Row 5:**10 X** vs. Row 19:**0 X**	-3.351	-3.828 to -2.873	Yes
**778**	Row 5:**10 X** vs. Row 19:**10 X**	-3.051	-3.528 to -2.573	Yes
**779**	Row 5:**10 X** vs. Row 19:**25 X**	-1.887	-2.314 to -1.460	Yes
**780**	Row 5:**10 X** vs. Row 20:**0 X**	-3.403	-3.880 to -2.926	Yes
**781**	Row 5:**10 X** vs. Row 20:**10 X**	-2.944	-3.421 to -2.467	Yes
**782**	Row 5:**10 X** vs. Row 20:**25 X**	-1.831	-2.258 to -1.404	Yes
**783**	Row 5:**10 X** vs. Row 21:**0 X**	-2.760	-3.237 to -2.283	Yes
**784**	Row 5:**10 X** vs. Row 21:**10 X**	-2.330	-2.807 to -1.852	Yes
**785**	Row 5:**10 X** vs. Row 21:**25 X**	-1.858	-2.285 to -1.431	Yes
**786**	Row 5:**25 X** vs. Row 6:**0 X**	-0.5987	-1.026 to -0.1718	Yes
**787**	Row 5:**25 X** vs. Row 6:**10 X**	-0.4940	-0.9209 to -0.06709	Yes
**788**	Row 5:**25 X** vs. Row 6:**25 X**	-0.4657	-0.8926 to -0.03875	Yes
**789**	Row 5:**25 X** vs. Row 7:**0 X**	-0.9780	-1.405 to -0.5511	Yes
**790**	Row 5:**25 X** vs. Row 7:**10 X**	-0.9007	-1.328 to -0.4738	Yes
**791**	Row 5:**25 X** vs. Row 7:**25 X**	-0.7780	-1.205 to -0.3511	Yes
**792**	Row 5:**25 X** vs. Row 8:**0 X**	-1.209	-1.636 to -0.7824	Yes
**793**	Row 5:**25 X** vs. Row 8:**10 X**	-1.313	-1.740 to -0.8858	Yes
**794**	Row 5:**25 X** vs. Row 8:**25 X**	-1.256	-1.683 to -0.8291	Yes
**795**	Row 5:**25 X** vs. Row 9:**0 X**	-1.564	-1.991 to -1.137	Yes
**796**	Row 5:**25 X** vs. Row 9:**10 X**	-1.548	-1.975 to -1.121	Yes
**797**	Row 5:**25 X** vs. Row 9:**25 X**	-1.364	-1.791 to -0.9374	Yes
**798**	Row 5:**25 X** vs. Row 10:**0 X**	-1.812	-2.239 to -1.385	Yes
**799**	Row 5:**25 X** vs. Row 10:**10 X**	-1.804	-2.231 to -1.377	Yes
**800**	Row 5:**25 X** vs. Row 10:**25 X**	-1.637	-2.064 to -1.210	Yes
**801**	Row 5:**25 X** vs. Row 11:**0 X**	-2.570	-2.997 to -2.143	Yes
**802**	Row 5:**25 X** vs. Row 11:**10 X**	-1.922	-2.399 to -1.445	Yes
**803**	Row 5:**25 X** vs. Row 11:**25 X**	-1.865	-2.292 to -1.438	Yes
**804**	Row 5:**25 X** vs. 11:**0 X**	-2.664	-3.091 to -2.237	Yes
**805**	Row 5:**25 X** vs. 11:**10 X**	-2.238	-2.715 to -1.761	Yes
**806**	Row 5:**25 X** vs. 11:**25 X**	-2.169	-2.596 to -1.742	Yes
**807**	Row 5:**25 X** vs. Row 13:**0 X**	-2.633	-3.060 to -2.206	Yes
**808**	Row 5:**25 X** vs. Row 13:**10 X**	-2.266	-2.743 to -1.789	Yes
**809**	Row 5:**25 X** vs. Row 13:**25 X**	-2.275	-2.702 to -1.848	Yes
**810**	Row 5:**25 X** vs. Row 14:**0 X**	-2.683	-3.110 to -2.256	Yes

	2way ANOVA<br>Multiple comparisons	A<br>Data Set-A<br>Y	B<br>Data Set-B<br>Y	C<br>Data Set-C<br>Y
811	Row 5:**25 X** vs. Row 14:**10 X**	-2.459	-2.886 to -2.032	Yes
812	Row 5:**25 X** vs. Row 14:**25 X**	-2.419	-2.846 to -1.992	Yes
813	Row 5:**25 X** vs. 14:**0 X**	-3.277	-3.704 to -2.850	Yes
814	Row 5:**25 X** vs. 14:**10 X**	-2.674	-3.151 to -2.197	Yes
815	Row 5:**25 X** vs. 14:**25 X**	-2.310	-2.787 to -1.833	Yes
816	Row 5:**25 X** vs. Row 16:**0 X**	-3.390	-3.867 to -2.913	Yes
817	Row 5:**25 X** vs. Row 16:**10 X**	-2.758	-3.235 to -2.281	Yes
818	Row 5:**25 X** vs. Row 16:**25 X**	-1.903	-2.380 to -1.426	Yes
819	Row 5:**25 X** vs. Row 17:**0 X**	-3.016	-3.493 to -2.538	Yes
820	Row 5:**25 X** vs. Row 17:**10 X**	-2.776	-3.253 to -2.298	Yes
821	Row 5:**25 X** vs. Row 17:**25 X**	-2.069	-2.546 to -1.592	Yes
822	Row 5:**25 X** vs. 17:**0 X**	-3.277	-3.754 to -2.799	Yes
823	Row 5:**25 X** vs. 17:**10 X**	-3.070	-3.547 to -2.593	Yes
824	Row 5:**25 X** vs. 17:**25 X**	-1.979	-2.406 to -1.552	Yes
825	Row 5:**25 X** vs. Row 19:**0 X**	-3.373	-3.850 to -2.895	Yes
826	Row 5:**25 X** vs. Row 19:**10 X**	-3.073	-3.550 to -2.595	Yes
827	Row 5:**25 X** vs. Row 19:**25 X**	-1.909	-2.336 to -1.482	Yes
828	Row 5:**25 X** vs. Row 20:**0 X**	-3.425	-3.902 to -2.948	Yes
829	Row 5:**25 X** vs. Row 20:**10 X**	-2.966	-3.443 to -2.489	Yes
830	Row 5:**25 X** vs. Row 20:**25 X**	-1.853	-2.280 to -1.426	Yes
831	Row 5:**25 X** vs. Row 21:**0 X**	-2.782	-3.259 to -2.305	Yes
832	Row 5:**25 X** vs. Row 21:**10 X**	-2.352	-2.829 to -1.874	Yes
833	Row 5:**25 X** vs. Row 21:**25 X**	-1.880	-2.307 to -1.453	Yes
834	Row 6:**0 X** vs. Row 6:**10 X**	0.1047	-0.3222 to 0.5316	No
835	Row 6:**0 X** vs. Row 6:**25 X**	0.1330	-0.2939 to 0.5599	No
836	Row 6:**0 X** vs. Row 7:**0 X**	-0.3793	-0.8062 to 0.04758	No
837	Row 6:**0 X** vs. Row 7:**10 X**	-0.3020	-0.7289 to 0.1249	No
838	Row 6:**0 X** vs. Row 7:**25 X**	-0.1793	-0.6062 to 0.2476	No
839	Row 6:**0 X** vs. Row 8:**0 X**	-0.6107	-1.038 to -0.1838	Yes
840	Row 6:**0 X** vs. Row 8:**10 X**	-0.7140	-1.141 to -0.2871	Yes
841	Row 6:**0 X** vs. Row 8:**25 X**	-0.6573	-1.084 to -0.2304	Yes
842	Row 6:**0 X** vs. Row 9:**0 X**	-0.9653	-1.392 to -0.5384	Yes
843	Row 6:**0 X** vs. Row 9:**10 X**	-0.9493	-1.376 to -0.5224	Yes
844	Row 6:**0 X** vs. Row 9:**25 X**	-0.7657	-1.193 to -0.3388	Yes
845	Row 6:**0 X** vs. Row 10:**0 X**	-1.213	-1.640 to -0.7864	Yes
846	Row 6:**0 X** vs. Row 10:**10 X**	-1.205	-1.632 to -0.7781	Yes
847	Row 6:**0 X** vs. Row 10:**25 X**	-1.038	-1.465 to -0.6114	Yes
848	Row 6:**0 X** vs. Row 11:**0 X**	-1.971	-2.398 to -1.544	Yes
849	Row 6:**0 X** vs. Row 11:**10 X**	-1.323	-1.801 to -0.8460	Yes
850	Row 6:**0 X** vs. Row 11:**25 X**	-1.266	-1.693 to -0.8391	Yes
851	Row 6:**0 X** vs. 11:**0 X**	-2.065	-2.492 to -1.638	Yes
852	Row 6:**0 X** vs. 11:**10 X**	-1.639	-2.117 to -1.162	Yes
853	Row 6:**0 X** vs. 11:**25 X**	-1.570	-1.997 to -1.143	Yes
854	Row 6:**0 X** vs. Row 13:**0 X**	-2.034	-2.461 to -1.607	Yes
855	Row 6:**0 X** vs. Row 13:**10 X**	-1.667	-2.145 to -1.190	Yes

	**2way ANOVA**<br>Multiple comparisons	**A**<br>Data Set-A<br>**Y**	**B**<br>Data Set-B<br>**Y**	**C**<br>Data Set-C<br>**Y**
**856**	Row 6:**0 X** vs. Row 13:**25 X**	-1.676	-2.103 to -1.249	Yes
**857**	Row 6:**0 X** vs. Row 14:**0 X**	-2.085	-2.512 to -1.658	Yes
**858**	Row 6:**0 X** vs. Row 14:**10 X**	-1.861	-2.288 to -1.434	Yes
**859**	Row 6:**0 X** vs. Row 14:**25 X**	-1.821	-2.248 to -1.394	Yes
**860**	Row 6:**0 X** vs. 14:**0 X**	-2.679	-3.106 to -2.252	Yes
**861**	Row 6:**0 X** vs. 14:**10 X**	-2.075	-2.553 to -1.598	Yes
**862**	Row 6:**0 X** vs. 14:**25 X**	-1.711	-2.189 to -1.234	Yes
**863**	Row 6:**0 X** vs. Row 16:**0 X**	-2.791	-3.269 to -2.314	Yes
**864**	Row 6:**0 X** vs. Row 16:**10 X**	-2.159	-2.637 to -1.682	Yes
**865**	Row 6:**0 X** vs. Row 16:**25 X**	-1.304	-1.782 to -0.8270	Yes
**866**	Row 6:**0 X** vs. Row 17:**0 X**	-2.417	-2.894 to -1.940	Yes
**867**	Row 6:**0 X** vs. Row 17:**10 X**	-2.177	-2.654 to -1.700	Yes
**868**	Row 6:**0 X** vs. Row 17:**25 X**	-1.470	-1.948 to -0.9930	Yes
**869**	Row 6:**0 X** vs. 17:**0 X**	-2.678	-3.155 to -2.201	Yes
**870**	Row 6:**0 X** vs. 17:**10 X**	-2.471	-2.949 to -1.994	Yes
**871**	Row 6:**0 X** vs. 17:**25 X**	-1.381	-1.808 to -0.9538	Yes
**872**	Row 6:**0 X** vs. Row 19:**0 X**	-2.774	-3.251 to -2.297	Yes
**873**	Row 6:**0 X** vs. Row 19:**10 X**	-2.474	-2.951 to -1.997	Yes
**874**	Row 6:**0 X** vs. Row 19:**25 X**	-1.310	-1.737 to -0.8834	Yes
**875**	Row 6:**0 X** vs. Row 20:**0 X**	-2.826	-3.304 to -2.349	Yes
**876**	Row 6:**0 X** vs. Row 20:**10 X**	-2.367	-2.845 to -1.890	Yes
**877**	Row 6:**0 X** vs. Row 20:**25 X**	-1.254	-1.681 to -0.8271	Yes
**878**	Row 6:**0 X** vs. Row 21:**0 X**	-2.183	-2.661 to -1.706	Yes
**879**	Row 6:**0 X** vs. Row 21:**10 X**	-1.753	-2.230 to -1.276	Yes
**880**	Row 6:**0 X** vs. Row 21:**25 X**	-1.281	-1.708 to -0.8544	Yes
**881**	Row 6:**10 X** vs. Row 6:**25 X**	0.02833	-0.3986 to 0.4552	No
**882**	Row 6:**10 X** vs. Row 7:**0 X**	-0.4840	-0.9109 to -0.05709	Yes
**883**	Row 6:**10 X** vs. Row 7:**10 X**	-0.4067	-0.8336 to 0.02025	No
**884**	Row 6:**10 X** vs. Row 7:**25 X**	-0.2840	-0.7109 to 0.1429	No
**885**	Row 6:**10 X** vs. Row 8:**0 X**	-0.7153	-1.142 to -0.2884	Yes
**886**	Row 6:**10 X** vs. Row 8:**10 X**	-0.8187	-1.246 to -0.3918	Yes
**887**	Row 6:**10 X** vs. Row 8:**25 X**	-0.7620	-1.189 to -0.3351	Yes
**888**	Row 6:**10 X** vs. Row 9:**0 X**	-1.070	-1.497 to -0.6431	Yes
**889**	Row 6:**10 X** vs. Row 9:**10 X**	-1.054	-1.481 to -0.6271	Yes
**890**	Row 6:**10 X** vs. Row 9:**25 X**	-0.8703	-1.297 to -0.4434	Yes
**891**	Row 6:**10 X** vs. Row 10:**0 X**	-1.318	-1.745 to -0.8911	Yes
**892**	Row 6:**10 X** vs. Row 10:**10 X**	-1.310	-1.737 to -0.8828	Yes
**893**	Row 6:**10 X** vs. Row 10:**25 X**	-1.143	-1.570 to -0.7161	Yes
**894**	Row 6:**10 X** vs. Row 11:**0 X**	-2.076	-2.503 to -1.649	Yes
**895**	Row 6:**10 X** vs. Row 11:**10 X**	-1.428	-1.905 to -0.9507	Yes
**896**	Row 6:**10 X** vs. Row 11:**25 X**	-1.371	-1.798 to -0.9438	Yes
**897**	Row 6:**10 X** vs. 11:**0 X**	-2.170	-2.597 to -1.743	Yes
**898**	Row 6:**10 X** vs. 11:**10 X**	-1.744	-2.221 to -1.267	Yes
**899**	Row 6:**10 X** vs. 11:**25 X**	-1.675	-2.102 to -1.248	Yes
**900**	Row 6:**10 X** vs. Row 13:**0 X**	-2.139	-2.566 to -1.712	Yes

	2way ANOVA<br>Multiple comparisons	A<br>Data Set-A<br>Y	B<br>Data Set-B<br>Y	C<br>Data Set-C<br>Y
**901**	Row 6:**10 X** vs. Row 13:**10 X**	-1.772	-2.249 to -1.295	Yes
**902**	Row 6:**10 X** vs. Row 13:**25 X**	-1.781	-2.208 to -1.354	Yes
**903**	Row 6:**10 X** vs. Row 14:**0 X**	-2.189	-2.616 to -1.762	Yes
**904**	Row 6:**10 X** vs. Row 14:**10 X**	-1.965	-2.392 to -1.538	Yes
**905**	Row 6:**10 X** vs. Row 14:**25 X**	-1.925	-2.352 to -1.498	Yes
**906**	Row 6:**10 X** vs. 14:**0 X**	-2.783	-3.210 to -2.356	Yes
**907**	Row 6:**10 X** vs. 14:**10 X**	-2.180	-2.657 to -1.703	Yes
**908**	Row 6:**10 X** vs. 14:**25 X**	-1.816	-2.293 to -1.339	Yes
**909**	Row 6:**10 X** vs. Row 16:**0 X**	-2.896	-3.373 to -2.419	Yes
**910**	Row 6:**10 X** vs. Row 16:**10 X**	-2.264	-2.741 to -1.787	Yes
**911**	Row 6:**10 X** vs. Row 16:**25 X**	-1.409	-1.886 to -0.9317	Yes
**912**	Row 6:**10 X** vs. Row 17:**0 X**	-2.522	-2.999 to -2.044	Yes
**913**	Row 6:**10 X** vs. Row 17:**10 X**	-2.282	-2.759 to -1.804	Yes
**914**	Row 6:**10 X** vs. Row 17:**25 X**	-1.575	-2.052 to -1.098	Yes
**915**	Row 6:**10 X** vs. 17:**0 X**	-2.783	-3.260 to -2.305	Yes
**916**	Row 6:**10 X** vs. 17:**10 X**	-2.576	-3.053 to -2.099	Yes
**917**	Row 6:**10 X** vs. 17:**25 X**	-1.485	-1.912 to -1.058	Yes
**918**	Row 6:**10 X** vs. Row 19:**0 X**	-2.879	-3.356 to -2.401	Yes
**919**	Row 6:**10 X** vs. Row 19:**10 X**	-2.579	-3.056 to -2.101	Yes
**920**	Row 6:**10 X** vs. Row 19:**25 X**	-1.415	-1.842 to -0.9881	Yes
**921**	Row 6:**10 X** vs. Row 20:**0 X**	-2.931	-3.408 to -2.454	Yes
**922**	Row 6:**10 X** vs. Row 20:**10 X**	-2.472	-2.949 to -1.995	Yes
**923**	Row 6:**10 X** vs. Row 20:**25 X**	-1.359	-1.786 to -0.9318	Yes
**924**	Row 6:**10 X** vs. Row 21:**0 X**	-2.288	-2.765 to -1.811	Yes
**925**	Row 6:**10 X** vs. Row 21:**10 X**	-1.858	-2.335 to -1.380	Yes
**926**	Row 6:**10 X** vs. Row 21:**25 X**	-1.386	-1.813 to -0.9591	Yes
**927**	Row 6:**25 X** vs. Row 7:**0 X**	-0.5123	-0.9392 to -0.08542	Yes
**928**	Row 6:**25 X** vs. Row 7:**10 X**	-0.4350	-0.8619 to -0.008088	Yes
**929**	Row 6:**25 X** vs. Row 7:**25 X**	-0.3123	-0.7392 to 0.1146	No
**930**	Row 6:**25 X** vs. Row 8:**0 X**	-0.7437	-1.171 to -0.3168	Yes
**931**	Row 6:**25 X** vs. Row 8:**10 X**	-0.8470	-1.274 to -0.4201	Yes
**932**	Row 6:**25 X** vs. Row 8:**25 X**	-0.7903	-1.217 to -0.3634	Yes
**933**	Row 6:**25 X** vs. Row 9:**0 X**	-1.098	-1.525 to -0.6714	Yes
**934**	Row 6:**25 X** vs. Row 9:**10 X**	-1.082	-1.509 to -0.6554	Yes
**935**	Row 6:**25 X** vs. Row 9:**25 X**	-0.8987	-1.326 to -0.4718	Yes
**936**	Row 6:**25 X** vs. Row 10:**0 X**	-1.346	-1.773 to -0.9194	Yes
**937**	Row 6:**25 X** vs. Row 10:**10 X**	-1.338	-1.765 to -0.9111	Yes
**938**	Row 6:**25 X** vs. Row 10:**25 X**	-1.171	-1.598 to -0.7444	Yes
**939**	Row 6:**25 X** vs. Row 11:**0 X**	-2.104	-2.531 to -1.677	Yes
**940**	Row 6:**25 X** vs. Row 11:**10 X**	-1.456	-1.934 to -0.9790	Yes
**941**	Row 6:**25 X** vs. Row 11:**25 X**	-1.399	-1.826 to -0.9721	Yes
**942**	Row 6:**25 X** vs. 11:**0 X**	-2.198	-2.625 to -1.771	Yes
**943**	Row 6:**25 X** vs. 11:**10 X**	-1.772	-2.250 to -1.295	Yes
**944**	Row 6:**25 X** vs. 11:**25 X**	-1.703	-2.130 to -1.276	Yes
**945**	Row 6:**25 X** vs. Row 13:**0 X**	-2.167	-2.594 to -1.740	Yes

	2way ANOVA Multiple comparisons	A Data Set-A Y	B Data Set-B Y	C Data Set-C Y
**946**	Row 6:**25 X** vs. Row 13:**10 X**	-1.800	-2.278 to -1.323	Yes
**947**	Row 6:**25 X** vs. Row 13:**25 X**	-1.809	-2.236 to -1.382	Yes
**948**	Row 6:**25 X** vs. Row 14:**0 X**	-2.218	-2.645 to -1.791	Yes
**949**	Row 6:**25 X** vs. Row 14:**10 X**	-1.994	-2.421 to -1.567	Yes
**950**	Row 6:**25 X** vs. Row 14:**25 X**	-1.954	-2.381 to -1.527	Yes
**951**	Row 6:**25 X** vs. 14:**0 X**	-2.812	-3.239 to -2.385	Yes
**952**	Row 6:**25 X** vs. 14:**10 X**	-2.208	-2.686 to -1.731	Yes
**953**	Row 6:**25 X** vs. 14:**25 X**	-1.844	-2.322 to -1.367	Yes
**954**	Row 6:**25 X** vs. Row 16:**0 X**	-2.924	-3.402 to -2.447	Yes
**955**	Row 6:**25 X** vs. Row 16:**10 X**	-2.292	-2.770 to -1.815	Yes
**956**	Row 6:**25 X** vs. Row 16:**25 X**	-1.437	-1.915 to -0.9600	Yes
**957**	Row 6:**25 X** vs. Row 17:**0 X**	-2.550	-3.027 to -2.073	Yes
**958**	Row 6:**25 X** vs. Row 17:**10 X**	-2.310	-2.787 to -1.833	Yes
**959**	Row 6:**25 X** vs. Row 17:**25 X**	-1.603	-2.081 to -1.126	Yes
**960**	Row 6:**25 X** vs. 17:**0 X**	-2.811	-3.288 to -2.334	Yes
**961**	Row 6:**25 X** vs. 17:**10 X**	-2.604	-3.082 to -2.127	Yes
**962**	Row 6:**25 X** vs. 17:**25 X**	-1.514	-1.941 to -1.087	Yes
**963**	Row 6:**25 X** vs. Row 19:**0 X**	-2.907	-3.384 to -2.430	Yes
**964**	Row 6:**25 X** vs. Row 19:**10 X**	-2.607	-3.084 to -2.130	Yes
**965**	Row 6:**25 X** vs. Row 19:**25 X**	-1.443	-1.870 to -1.016	Yes
**966**	Row 6:**25 X** vs. Row 20:**0 X**	-2.959	-3.437 to -2.482	Yes
**967**	Row 6:**25 X** vs. Row 20:**10 X**	-2.500	-2.978 to -2.023	Yes
**968**	Row 6:**25 X** vs. Row 20:**25 X**	-1.387	-1.814 to -0.9601	Yes
**969**	Row 6:**25 X** vs. Row 21:**0 X**	-2.316	-2.794 to -1.839	Yes
**970**	Row 6:**25 X** vs. Row 21:**10 X**	-1.886	-2.363 to -1.409	Yes
**971**	Row 6:**25 X** vs. Row 21:**25 X**	-1.414	-1.841 to -0.9874	Yes
**972**	Row 7:**0 X** vs. Row 7:**10 X**	0.07733	-0.3496 to 0.5042	No
**973**	Row 7:**0 X** vs. Row 7:**25 X**	0.2000	-0.2269 to 0.6269	No
**974**	Row 7:**0 X** vs. Row 8:**0 X**	-0.2313	-0.6582 to 0.1956	No
**975**	Row 7:**0 X** vs. Row 8:**10 X**	-0.3347	-0.7616 to 0.09225	No
**976**	Row 7:**0 X** vs. Row 8:**25 X**	-0.2780	-0.7049 to 0.1489	No
**977**	Row 7:**0 X** vs. Row 9:**0 X**	-0.5860	-1.013 to -0.1591	Yes
**978**	Row 7:**0 X** vs. Row 9:**10 X**	-0.5700	-0.9969 to -0.1431	Yes
**979**	Row 7:**0 X** vs. Row 9:**25 X**	-0.3863	-0.8132 to 0.04058	No
**980**	Row 7:**0 X** vs. Row 10:**0 X**	-0.8340	-1.261 to -0.4071	Yes
**981**	Row 7:**0 X** vs. Row 10:**10 X**	-0.8257	-1.253 to -0.3988	Yes
**982**	Row 7:**0 X** vs. Row 10:**25 X**	-0.6590	-1.086 to -0.2321	Yes
**983**	Row 7:**0 X** vs. Row 11:**0 X**	-1.592	-2.019 to -1.165	Yes
**984**	Row 7:**0 X** vs. Row 11:**10 X**	-0.9440	-1.421 to -0.4667	Yes
**985**	Row 7:**0 X** vs. Row 11:**25 X**	-0.8867	-1.314 to -0.4598	Yes
**986**	Row 7:**0 X** vs. 11:**0 X**	-1.686	-2.113 to -1.259	Yes
**987**	Row 7:**0 X** vs. 11:**10 X**	-1.260	-1.737 to -0.7827	Yes
**988**	Row 7:**0 X** vs. 11:**25 X**	-1.191	-1.618 to -0.7638	Yes
**989**	Row 7:**0 X** vs. Row 13:**0 X**	-1.655	-2.082 to -1.228	Yes
**990**	Row 7:**0 X** vs. Row 13:**10 X**	-1.288	-1.765 to -0.8107	Yes

	2way ANOVA<br>Multiple comparisons	A<br>Data Set-A<br>Y	B<br>Data Set-B<br>Y	C<br>Data Set-C<br>Y
991	Row 7:**0 X** vs. Row 13:**25 X**	-1.297	-1.724 to -0.8698	Yes
992	Row 7:**0 X** vs. Row 14:**0 X**	-1.705	-2.132 to -1.278	Yes
993	Row 7:**0 X** vs. Row 14:**10 X**	-1.481	-1.908 to -1.054	Yes
994	Row 7:**0 X** vs. Row 14:**25 X**	-1.441	-1.868 to -1.014	Yes
995	Row 7:**0 X** vs. 14:**0 X**	-2.299	-2.726 to -1.872	Yes
996	Row 7:**0 X** vs. 14:**10 X**	-1.696	-2.173 to -1.219	Yes
997	Row 7:**0 X** vs. 14:**25 X**	-1.332	-1.809 to -0.8547	Yes
998	Row 7:**0 X** vs. Row 16:**0 X**	-2.412	-2.889 to -1.935	Yes
999	Row 7:**0 X** vs. Row 16:**10 X**	-1.780	-2.257 to -1.303	Yes
1000	Row 7:**0 X** vs. Row 16:**25 X**	-0.9250	-1.402 to -0.4477	Yes
1001	Row 7:**0 X** vs. Row 17:**0 X**	-2.038	-2.515 to -1.560	Yes
1002	Row 7:**0 X** vs. Row 17:**10 X**	-1.798	-2.275 to -1.320	Yes
1003	Row 7:**0 X** vs. Row 17:**25 X**	-1.091	-1.568 to -0.6137	Yes
1004	Row 7:**0 X** vs. 17:**0 X**	-2.299	-2.776 to -1.821	Yes
1005	Row 7:**0 X** vs. 17:**10 X**	-2.092	-2.569 to -1.615	Yes
1006	Row 7:**0 X** vs. 17:**25 X**	-1.001	-1.428 to -0.5744	Yes
1007	Row 7:**0 X** vs. Row 19:**0 X**	-2.395	-2.872 to -1.917	Yes
1008	Row 7:**0 X** vs. Row 19:**10 X**	-2.095	-2.572 to -1.617	Yes
1009	Row 7:**0 X** vs. Row 19:**25 X**	-0.9310	-1.358 to -0.5041	Yes
1010	Row 7:**0 X** vs. Row 20:**0 X**	-2.447	-2.924 to -1.970	Yes
1011	Row 7:**0 X** vs. Row 20:**10 X**	-1.988	-2.465 to -1.511	Yes
1012	Row 7:**0 X** vs. Row 20:**25 X**	-0.8747	-1.302 to -0.4478	Yes
1013	Row 7:**0 X** vs. Row 21:**0 X**	-1.804	-2.281 to -1.327	Yes
1014	Row 7:**0 X** vs. Row 21:**10 X**	-1.374	-1.851 to -0.8962	Yes
1015	Row 7:**0 X** vs. Row 21:**25 X**	-0.9020	-1.329 to -0.4751	Yes
1016	Row 7:**10 X** vs. Row 7:**25 X**	0.1227	-0.3042 to 0.5496	No
1017	Row 7:**10 X** vs. Row 8:**0 X**	-0.3087	-0.7356 to 0.1182	No
1018	Row 7:**10 X** vs. Row 8:**10 X**	-0.4120	-0.8389 to 0.01491	No
1019	Row 7:**10 X** vs. Row 8:**25 X**	-0.3553	-0.7822 to 0.07158	No
1020	Row 7:**10 X** vs. Row 9:**0 X**	-0.6633	-1.090 to -0.2364	Yes
1021	Row 7:**10 X** vs. Row 9:**10 X**	-0.6473	-1.074 to -0.2204	Yes
1022	Row 7:**10 X** vs. Row 9:**25 X**	-0.4637	-0.8906 to -0.03675	Yes
1023	Row 7:**10 X** vs. Row 10:**0 X**	-0.9113	-1.338 to -0.4844	Yes
1024	Row 7:**10 X** vs. Row 10:**10 X**	-0.9030	-1.330 to -0.4761	Yes
1025	Row 7:**10 X** vs. Row 10:**25 X**	-0.7363	-1.163 to -0.3094	Yes
1026	Row 7:**10 X** vs. Row 11:**0 X**	-1.669	-2.096 to -1.242	Yes
1027	Row 7:**10 X** vs. Row 11:**10 X**	-1.021	-1.499 to -0.5440	Yes
1028	Row 7:**10 X** vs. Row 11:**25 X**	-0.9640	-1.391 to -0.5371	Yes
1029	Row 7:**10 X** vs. 11:**0 X**	-1.763	-2.190 to -1.336	Yes
1030	Row 7:**10 X** vs. 11:**10 X**	-1.337	-1.815 to -0.8600	Yes
1031	Row 7:**10 X** vs. 11:**25 X**	-1.268	-1.695 to -0.8411	Yes
1032	Row 7:**10 X** vs. Row 13:**0 X**	-1.732	-2.159 to -1.305	Yes
1033	Row 7:**10 X** vs. Row 13:**10 X**	-1.365	-1.843 to -0.8880	Yes
1034	Row 7:**10 X** vs. Row 13:**25 X**	-1.374	-1.801 to -0.9471	Yes
1035	Row 7:**10 X** vs. Row 14:**0 X**	-1.783	-2.210 to -1.356	Yes

	**2way ANOVA**<br>Multiple comparisons	**A**<br>Data Set-A<br>**Y**	**B**<br>Data Set-B<br>**Y**	**C**<br>Data Set-C<br>**Y**
**1036**	Row 7:**10 X** vs. Row 14:**10 X**	-1.559	-1.986 to -1.132	Yes
**1037**	Row 7:**10 X** vs. Row 14:**25 X**	-1.519	-1.946 to -1.092	Yes
**1038**	Row 7:**10 X** vs. 14:**0 X**	-2.377	-2.804 to -1.950	Yes
**1039**	Row 7:**10 X** vs. 14:**10 X**	-1.773	-2.251 to -1.296	Yes
**1040**	Row 7:**10 X** vs. 14:**25 X**	-1.409	-1.887 to -0.9320	Yes
**1041**	Row 7:**10 X** vs. Row 16:**0 X**	-2.489	-2.967 to -2.012	Yes
**1042**	Row 7:**10 X** vs. Row 16:**10 X**	-1.857	-2.335 to -1.380	Yes
**1043**	Row 7:**10 X** vs. Row 16:**25 X**	-1.002	-1.480 to -0.5250	Yes
**1044**	Row 7:**10 X** vs. Row 17:**0 X**	-2.115	-2.592 to -1.638	Yes
**1045**	Row 7:**10 X** vs. Row 17:**10 X**	-1.875	-2.352 to -1.398	Yes
**1046**	Row 7:**10 X** vs. Row 17:**25 X**	-1.168	-1.646 to -0.6910	Yes
**1047**	Row 7:**10 X** vs. 17:**0 X**	-2.376	-2.853 to -1.899	Yes
**1048**	Row 7:**10 X** vs. 17:**10 X**	-2.169	-2.647 to -1.692	Yes
**1049**	Row 7:**10 X** vs. 17:**25 X**	-1.079	-1.506 to -0.6518	Yes
**1050**	Row 7:**10 X** vs. Row 19:**0 X**	-2.472	-2.949 to -1.995	Yes
**1051**	Row 7:**10 X** vs. Row 19:**10 X**	-2.172	-2.649 to -1.695	Yes
**1052**	Row 7:**10 X** vs. Row 19:**25 X**	-1.008	-1.435 to -0.5814	Yes
**1053**	Row 7:**10 X** vs. Row 20:**0 X**	-2.524	-3.002 to -2.047	Yes
**1054**	Row 7:**10 X** vs. Row 20:**10 X**	-2.065	-2.543 to -1.588	Yes
**1055**	Row 7:**10 X** vs. Row 20:**25 X**	-0.9520	-1.379 to -0.5251	Yes
**1056**	Row 7:**10 X** vs. Row 21:**0 X**	-1.881	-2.359 to -1.404	Yes
**1057**	Row 7:**10 X** vs. Row 21:**10 X**	-1.451	-1.928 to -0.9735	Yes
**1058**	Row 7:**10 X** vs. Row 21:**25 X**	-0.9793	-1.406 to -0.5524	Yes
**1059**	Row 7:**25 X** vs. Row 8:**0 X**	-0.4313	-0.8582 to -0.004421	Yes
**1060**	Row 7:**25 X** vs. Row 8:**10 X**	-0.5347	-0.9616 to -0.1078	Yes
**1061**	Row 7:**25 X** vs. Row 8:**25 X**	-0.4780	-0.9049 to -0.05109	Yes
**1062**	Row 7:**25 X** vs. Row 9:**0 X**	-0.7860	-1.213 to -0.3591	Yes
**1063**	Row 7:**25 X** vs. Row 9:**10 X**	-0.7700	-1.197 to -0.3431	Yes
**1064**	Row 7:**25 X** vs. Row 9:**25 X**	-0.5863	-1.013 to -0.1594	Yes
**1065**	Row 7:**25 X** vs. Row 10:**0 X**	-1.034	-1.461 to -0.6071	Yes
**1066**	Row 7:**25 X** vs. Row 10:**10 X**	-1.026	-1.453 to -0.5988	Yes
**1067**	Row 7:**25 X** vs. Row 10:**25 X**	-0.8590	-1.286 to -0.4321	Yes
**1068**	Row 7:**25 X** vs. Row 11:**0 X**	-1.792	-2.219 to -1.365	Yes
**1069**	Row 7:**25 X** vs. Row 11:**10 X**	-1.144	-1.621 to -0.6667	Yes
**1070**	Row 7:**25 X** vs. Row 11:**25 X**	-1.087	-1.514 to -0.6598	Yes
**1071**	Row 7:**25 X** vs. 11:**0 X**	-1.886	-2.313 to -1.459	Yes
**1072**	Row 7:**25 X** vs. 11:**10 X**	-1.460	-1.937 to -0.9827	Yes
**1073**	Row 7:**25 X** vs. 11:**25 X**	-1.391	-1.818 to -0.9638	Yes
**1074**	Row 7:**25 X** vs. Row 13:**0 X**	-1.855	-2.282 to -1.428	Yes
**1075**	Row 7:**25 X** vs. Row 13:**10 X**	-1.488	-1.965 to -1.011	Yes
**1076**	Row 7:**25 X** vs. Row 13:**25 X**	-1.497	-1.924 to -1.070	Yes
**1077**	Row 7:**25 X** vs. Row 14:**0 X**	-1.905	-2.332 to -1.478	Yes
**1078**	Row 7:**25 X** vs. Row 14:**10 X**	-1.681	-2.108 to -1.254	Yes
**1079**	Row 7:**25 X** vs. Row 14:**25 X**	-1.641	-2.068 to -1.214	Yes
**1080**	Row 7:**25 X** vs. 14:**0 X**	-2.499	-2.926 to -2.072	Yes

	**2way ANOVA**<br>Multiple comparisons	**A**<br>Data Set-A<br>**Y**	**B**<br>Data Set-B<br>**Y**	**C**<br>Data Set-C<br>**Y**
**1081**	Row 7:**25 X** vs. 14:**10 X**	-1.896	-2.373 to -1.419	Yes
**1082**	Row 7:**25 X** vs. 14:**25 X**	-1.532	-2.009 to -1.055	Yes
**1083**	Row 7:**25 X** vs. Row 16:**0 X**	-2.612	-3.089 to -2.135	Yes
**1084**	Row 7:**25 X** vs. Row 16:**10 X**	-1.980	-2.457 to -1.503	Yes
**1085**	Row 7:**25 X** vs. Row 16:**25 X**	-1.125	-1.602 to -0.6477	Yes
**1086**	Row 7:**25 X** vs. Row 17:**0 X**	-2.238	-2.715 to -1.760	Yes
**1087**	Row 7:**25 X** vs. Row 17:**10 X**	-1.998	-2.475 to -1.520	Yes
**1088**	Row 7:**25 X** vs. Row 17:**25 X**	-1.291	-1.768 to -0.8137	Yes
**1089**	Row 7:**25 X** vs. 17:**0 X**	-2.499	-2.976 to -2.021	Yes
**1090**	Row 7:**25 X** vs. 17:**10 X**	-2.292	-2.769 to -1.815	Yes
**1091**	Row 7:**25 X** vs. 17:**25 X**	-1.201	-1.628 to -0.7744	Yes
**1092**	Row 7:**25 X** vs. Row 19:**0 X**	-2.595	-3.072 to -2.117	Yes
**1093**	Row 7:**25 X** vs. Row 19:**10 X**	-2.295	-2.772 to -1.817	Yes
**1094**	Row 7:**25 X** vs. Row 19:**25 X**	-1.131	-1.558 to -0.7041	Yes
**1095**	Row 7:**25 X** vs. Row 20:**0 X**	-2.647	-3.124 to -2.170	Yes
**1096**	Row 7:**25 X** vs. Row 20:**10 X**	-2.188	-2.665 to -1.711	Yes
**1097**	Row 7:**25 X** vs. Row 20:**25 X**	-1.075	-1.502 to -0.6478	Yes
**1098**	Row 7:**25 X** vs. Row 21:**0 X**	-2.004	-2.481 to -1.527	Yes
**1099**	Row 7:**25 X** vs. Row 21:**10 X**	-1.574	-2.051 to -1.096	Yes
**1100**	Row 7:**25 X** vs. Row 21:**25 X**	-1.102	-1.529 to -0.6751	Yes
**1101**	Row 8:**0 X** vs. Row 8:**10 X**	-0.1033	-0.5302 to 0.3236	No
**1102**	Row 8:**0 X** vs. Row 8:**25 X**	-0.04667	-0.4736 to 0.3802	No
**1103**	Row 8:**0 X** vs. Row 9:**0 X**	-0.3547	-0.7816 to 0.07225	No
**1104**	Row 8:**0 X** vs. Row 9:**10 X**	-0.3387	-0.7656 to 0.08825	No
**1105**	Row 8:**0 X** vs. Row 9:**25 X**	-0.1550	-0.5819 to 0.2719	No
**1106**	Row 8:**0 X** vs. Row 10:**0 X**	-0.6027	-1.030 to -0.1758	Yes
**1107**	Row 8:**0 X** vs. Row 10:**10 X**	-0.5943	-1.021 to -0.1674	Yes
**1108**	Row 8:**0 X** vs. Row 10:**25 X**	-0.4277	-0.8546 to -0.0007542	Yes
**1109**	Row 8:**0 X** vs. Row 11:**0 X**	-1.361	-1.788 to -0.9338	Yes
**1110**	Row 8:**0 X** vs. Row 11:**10 X**	-0.7127	-1.190 to -0.2354	Yes
**1111**	Row 8:**0 X** vs. Row 11:**25 X**	-0.6553	-1.082 to -0.2284	Yes
**1112**	Row 8:**0 X** vs. 11:**0 X**	-1.454	-1.881 to -1.027	Yes
**1113**	Row 8:**0 X** vs. 11:**10 X**	-1.029	-1.506 to -0.5514	Yes
**1114**	Row 8:**0 X** vs. 11:**25 X**	-0.9593	-1.386 to -0.5324	Yes
**1115**	Row 8:**0 X** vs. Row 13:**0 X**	-1.423	-1.850 to -0.9964	Yes
**1116**	Row 8:**0 X** vs. Row 13:**10 X**	-1.057	-1.534 to -0.5794	Yes
**1117**	Row 8:**0 X** vs. Row 13:**25 X**	-1.065	-1.492 to -0.6384	Yes
**1118**	Row 8:**0 X** vs. Row 14:**0 X**	-1.474	-1.901 to -1.047	Yes
**1119**	Row 8:**0 X** vs. Row 14:**10 X**	-1.250	-1.677 to -0.8231	Yes
**1120**	Row 8:**0 X** vs. Row 14:**25 X**	-1.210	-1.637 to -0.7831	Yes
**1121**	Row 8:**0 X** vs. 14:**0 X**	-2.068	-2.495 to -1.641	Yes
**1122**	Row 8:**0 X** vs. 14:**10 X**	-1.465	-1.942 to -0.9874	Yes
**1123**	Row 8:**0 X** vs. 14:**25 X**	-1.101	-1.578 to -0.6234	Yes
**1124**	Row 8:**0 X** vs. Row 16:**0 X**	-2.181	-2.658 to -1.703	Yes
**1125**	Row 8:**0 X** vs. Row 16:**10 X**	-1.549	-2.026 to -1.071	Yes

	2way ANOVA<br>Multiple comparisons	A<br>Data Set-A<br>Y	B<br>Data Set-B<br>Y	C<br>Data Set-C<br>Y
1126	Row 8:**0 X** vs. Row 16:**25 X**	-0.6937	-1.171 to -0.2164	Yes
1127	Row 8:**0 X** vs. Row 17:**0 X**	-1.806	-2.283 to -1.329	Yes
1128	Row 8:**0 X** vs. Row 17:**10 X**	-1.566	-2.043 to -1.089	Yes
1129	Row 8:**0 X** vs. Row 17:**25 X**	-0.8597	-1.337 to -0.3824	Yes
1130	Row 8:**0 X** vs. 17:**0 X**	-2.067	-2.544 to -1.590	Yes
1131	Row 8:**0 X** vs. 17:**10 X**	-1.861	-2.338 to -1.383	Yes
1132	Row 8:**0 X** vs. 17:**25 X**	-0.7700	-1.197 to -0.3431	Yes
1133	Row 8:**0 X** vs. Row 19:**0 X**	-2.163	-2.640 to -1.686	Yes
1134	Row 8:**0 X** vs. Row 19:**10 X**	-1.863	-2.340 to -1.386	Yes
1135	Row 8:**0 X** vs. Row 19:**25 X**	-0.6997	-1.127 to -0.2728	Yes
1136	Row 8:**0 X** vs. Row 20:**0 X**	-2.216	-2.693 to -1.738	Yes
1137	Row 8:**0 X** vs. Row 20:**10 X**	-1.757	-2.234 to -1.279	Yes
1138	Row 8:**0 X** vs. Row 20:**25 X**	-0.6433	-1.070 to -0.2164	Yes
1139	Row 8:**0 X** vs. Row 21:**0 X**	-1.573	-2.050 to -1.095	Yes
1140	Row 8:**0 X** vs. Row 21:**10 X**	-1.142	-1.619 to -0.6649	Yes
1141	Row 8:**0 X** vs. Row 21:**25 X**	-0.6707	-1.098 to -0.2438	Yes
1142	Row 8:**10 X** vs. Row 8:**25 X**	0.05667	-0.3702 to 0.4836	No
1143	Row 8:**10 X** vs. Row 9:**0 X**	-0.2513	-0.6782 to 0.1756	No
1144	Row 8:**10 X** vs. Row 9:**10 X**	-0.2353	-0.6622 to 0.1916	No
1145	Row 8:**10 X** vs. Row 9:**25 X**	-0.05167	-0.4786 to 0.3752	No
1146	Row 8:**10 X** vs. Row 10:**0 X**	-0.4993	-0.9262 to -0.07242	Yes
1147	Row 8:**10 X** vs. Row 10:**10 X**	-0.4910	-0.9179 to -0.06409	Yes
1148	Row 8:**10 X** vs. Row 10:**25 X**	-0.3243	-0.7512 to 0.1026	No
1149	Row 8:**10 X** vs. Row 11:**0 X**	-1.257	-1.684 to -0.8304	Yes
1150	Row 8:**10 X** vs. Row 11:**10 X**	-0.6093	-1.087 to -0.1320	Yes
1151	Row 8:**10 X** vs. Row 11:**25 X**	-0.5520	-0.9789 to -0.1251	Yes
1152	Row 8:**10 X** vs. 11:**0 X**	-1.351	-1.778 to -0.9241	Yes
1153	Row 8:**10 X** vs. 11:**10 X**	-0.9253	-1.403 to -0.4480	Yes
1154	Row 8:**10 X** vs. 11:**25 X**	-0.8560	-1.283 to -0.4291	Yes
1155	Row 8:**10 X** vs. Row 13:**0 X**	-1.320	-1.747 to -0.8931	Yes
1156	Row 8:**10 X** vs. Row 13:**10 X**	-0.9533	-1.431 to -0.4760	Yes
1157	Row 8:**10 X** vs. Row 13:**25 X**	-0.9620	-1.389 to -0.5351	Yes
1158	Row 8:**10 X** vs. Row 14:**0 X**	-1.371	-1.798 to -0.9438	Yes
1159	Row 8:**10 X** vs. Row 14:**10 X**	-1.147	-1.574 to -0.7198	Yes
1160	Row 8:**10 X** vs. Row 14:**25 X**	-1.107	-1.534 to -0.6798	Yes
1161	Row 8:**10 X** vs. 14:**0 X**	-1.965	-2.392 to -1.538	Yes
1162	Row 8:**10 X** vs. 14:**10 X**	-1.361	-1.839 to -0.8840	Yes
1163	Row 8:**10 X** vs. 14:**25 X**	-0.9973	-1.475 to -0.5200	Yes
1164	Row 8:**10 X** vs. Row 16:**0 X**	-2.077	-2.555 to -1.600	Yes
1165	Row 8:**10 X** vs. Row 16:**10 X**	-1.445	-1.923 to -0.9680	Yes
1166	Row 8:**10 X** vs. Row 16:**25 X**	-0.5903	-1.068 to -0.1130	Yes
1167	Row 8:**10 X** vs. Row 17:**0 X**	-1.703	-2.180 to -1.226	Yes
1168	Row 8:**10 X** vs. Row 17:**10 X**	-1.463	-1.940 to -0.9855	Yes
1169	Row 8:**10 X** vs. Row 17:**25 X**	-0.7563	-1.234 to -0.2790	Yes
1170	Row 8:**10 X** vs. 17:**0 X**	-1.964	-2.441 to -1.487	Yes

	**2way ANOVA** Multiple comparisons	**A** Data Set-A **Y**	**B** Data Set-B **Y**	**C** Data Set-C **Y**
**1171**	Row 8:**10 X** vs. 17:**10 X**	-1.757	-2.235 to -1.280	Yes
**1172**	Row 8:**10 X** vs. 17:**25 X**	-0.6667	-1.094 to -0.2398	Yes
**1173**	Row 8:**10 X** vs. Row 19:**0 X**	-2.060	-2.537 to -1.583	Yes
**1174**	Row 8:**10 X** vs. Row 19:**10 X**	-1.760	-2.237 to -1.283	Yes
**1175**	Row 8:**10 X** vs. Row 19:**25 X**	-0.5963	-1.023 to -0.1694	Yes
**1176**	Row 8:**10 X** vs. Row 20:**0 X**	-2.112	-2.590 to -1.635	Yes
**1177**	Row 8:**10 X** vs. Row 20:**10 X**	-1.653	-2.131 to -1.176	Yes
**1178**	Row 8:**10 X** vs. Row 20:**25 X**	-0.5400	-0.9669 to -0.1131	Yes
**1179**	Row 8:**10 X** vs. Row 21:**0 X**	-1.469	-1.947 to -0.9920	Yes
**1180**	Row 8:**10 X** vs. Row 21:**10 X**	-1.039	-1.516 to -0.5615	Yes
**1181**	Row 8:**10 X** vs. Row 21:**25 X**	-0.5673	-0.9942 to -0.1404	Yes
**1182**	Row 8:**25 X** vs. Row 9:**0 X**	-0.3080	-0.7349 to 0.1189	No
**1183**	Row 8:**25 X** vs. Row 9:**10 X**	-0.2920	-0.7189 to 0.1349	No
**1184**	Row 8:**25 X** vs. Row 9:**25 X**	-0.1083	-0.5352 to 0.3186	No
**1185**	Row 8:**25 X** vs. Row 10:**0 X**	-0.5560	-0.9829 to -0.1291	Yes
**1186**	Row 8:**25 X** vs. Row 10:**10 X**	-0.5477	-0.9746 to -0.1208	Yes
**1187**	Row 8:**25 X** vs. Row 10:**25 X**	-0.3810	-0.8079 to 0.04591	No
**1188**	Row 8:**25 X** vs. Row 11:**0 X**	-1.314	-1.741 to -0.8871	Yes
**1189**	Row 8:**25 X** vs. Row 11:**10 X**	-0.6660	-1.143 to -0.1887	Yes
**1190**	Row 8:**25 X** vs. Row 11:**25 X**	-0.6087	-1.036 to -0.1818	Yes
**1191**	Row 8:**25 X** vs. 11:**0 X**	-1.408	-1.835 to -0.9808	Yes
**1192**	Row 8:**25 X** vs. 11:**10 X**	-0.9820	-1.459 to -0.5047	Yes
**1193**	Row 8:**25 X** vs. 11:**25 X**	-0.9127	-1.340 to -0.4858	Yes
**1194**	Row 8:**25 X** vs. Row 13:**0 X**	-1.377	-1.804 to -0.9498	Yes
**1195**	Row 8:**25 X** vs. Row 13:**10 X**	-1.010	-1.487 to -0.5327	Yes
**1196**	Row 8:**25 X** vs. Row 13:**25 X**	-1.019	-1.446 to -0.5918	Yes
**1197**	Row 8:**25 X** vs. Row 14:**0 X**	-1.427	-1.854 to -1.000	Yes
**1198**	Row 8:**25 X** vs. Row 14:**10 X**	-1.203	-1.630 to -0.7764	Yes
**1199**	Row 8:**25 X** vs. Row 14:**25 X**	-1.163	-1.590 to -0.7364	Yes
**1200**	Row 8:**25 X** vs. 14:**0 X**	-2.021	-2.448 to -1.594	Yes
**1201**	Row 8:**25 X** vs. 14:**10 X**	-1.418	-1.895 to -0.9407	Yes
**1202**	Row 8:**25 X** vs. 14:**25 X**	-1.054	-1.531 to -0.5767	Yes
**1203**	Row 8:**25 X** vs. Row 16:**0 X**	-2.134	-2.611 to -1.657	Yes
**1204**	Row 8:**25 X** vs. Row 16:**10 X**	-1.502	-1.979 to -1.025	Yes
**1205**	Row 8:**25 X** vs. Row 16:**25 X**	-0.6470	-1.124 to -0.1697	Yes
**1206**	Row 8:**25 X** vs. Row 17:**0 X**	-1.760	-2.237 to -1.282	Yes
**1207**	Row 8:**25 X** vs. Row 17:**10 X**	-1.520	-1.997 to -1.042	Yes
**1208**	Row 8:**25 X** vs. Row 17:**25 X**	-0.8130	-1.290 to -0.3357	Yes
**1209**	Row 8:**25 X** vs. 17:**0 X**	-2.021	-2.498 to -1.543	Yes
**1210**	Row 8:**25 X** vs. 17:**10 X**	-1.814	-2.291 to -1.337	Yes
**1211**	Row 8:**25 X** vs. 17:**25 X**	-0.7233	-1.150 to -0.2964	Yes
**1212**	Row 8:**25 X** vs. Row 19:**0 X**	-2.117	-2.594 to -1.639	Yes
**1213**	Row 8:**25 X** vs. Row 19:**10 X**	-1.817	-2.294 to -1.339	Yes
**1214**	Row 8:**25 X** vs. Row 19:**25 X**	-0.6530	-1.080 to -0.2261	Yes
**1215**	Row 8:**25 X** vs. Row 20:**0 X**	-2.169	-2.646 to -1.692	Yes

	2way ANOVA<br>Multiple comparisons	A<br>Data Set-A<br>Y	B<br>Data Set-B<br>Y	C<br>Data Set-C<br>Y
**1216**	Row 8:**25 X** vs. Row 20:**10 X**	-1.710	-2.187 to -1.233	Yes
**1217**	Row 8:**25 X** vs. Row 20:**25 X**	-0.5967	-1.024 to -0.1698	Yes
**1218**	Row 8:**25 X** vs. Row 21:**0 X**	-1.526	-2.003 to -1.049	Yes
**1219**	Row 8:**25 X** vs. Row 21:**10 X**	-1.096	-1.573 to -0.6182	Yes
**1220**	Row 8:**25 X** vs. Row 21:**25 X**	-0.6240	-1.051 to -0.1971	Yes
**1221**	Row 9:**0 X** vs. Row 9:**10 X**	0.01600	-0.4109 to 0.4429	No
**1222**	Row 9:**0 X** vs. Row 9:**25 X**	0.1997	-0.2272 to 0.6266	No
**1223**	Row 9:**0 X** vs. Row 10:**0 X**	-0.2480	-0.6749 to 0.1789	No
**1224**	Row 9:**0 X** vs. Row 10:**10 X**	-0.2397	-0.6666 to 0.1872	No
**1225**	Row 9:**0 X** vs. Row 10:**25 X**	-0.07300	-0.4999 to 0.3539	No
**1226**	Row 9:**0 X** vs. Row 11:**0 X**	-1.006	-1.433 to -0.5791	Yes
**1227**	Row 9:**0 X** vs. Row 11:**10 X**	-0.3580	-0.8353 to 0.1193	No
**1228**	Row 9:**0 X** vs. Row 11:**25 X**	-0.3007	-0.7276 to 0.1262	No
**1229**	Row 9:**0 X** vs. 11:**0 X**	-1.100	-1.527 to -0.6728	Yes
**1230**	Row 9:**0 X** vs. 11:**10 X**	-0.6740	-1.151 to -0.1967	Yes
**1231**	Row 9:**0 X** vs. 11:**25 X**	-0.6047	-1.032 to -0.1778	Yes
**1232**	Row 9:**0 X** vs. Row 13:**0 X**	-1.069	-1.496 to -0.6418	Yes
**1233**	Row 9:**0 X** vs. Row 13:**10 X**	-0.7020	-1.179 to -0.2247	Yes
**1234**	Row 9:**0 X** vs. Row 13:**25 X**	-0.7107	-1.138 to -0.2838	Yes
**1235**	Row 9:**0 X** vs. Row 14:**0 X**	-1.119	-1.546 to -0.6924	Yes
**1236**	Row 9:**0 X** vs. Row 14:**10 X**	-0.8953	-1.322 to -0.4684	Yes
**1237**	Row 9:**0 X** vs. Row 14:**25 X**	-0.8553	-1.282 to -0.4284	Yes
**1238**	Row 9:**0 X** vs. 14:**0 X**	-1.713	-2.140 to -1.286	Yes
**1239**	Row 9:**0 X** vs. 14:**10 X**	-1.110	-1.587 to -0.6327	Yes
**1240**	Row 9:**0 X** vs. 14:**25 X**	-0.7460	-1.223 to -0.2687	Yes
**1241**	Row 9:**0 X** vs. Row 16:**0 X**	-1.826	-2.303 to -1.349	Yes
**1242**	Row 9:**0 X** vs. Row 16:**10 X**	-1.194	-1.671 to -0.7167	Yes
**1243**	Row 9:**0 X** vs. Row 16:**25 X**	-0.3390	-0.8163 to 0.1383	No
**1244**	Row 9:**0 X** vs. Row 17:**0 X**	-1.452	-1.929 to -0.9742	Yes
**1245**	Row 9:**0 X** vs. Row 17:**10 X**	-1.212	-1.689 to -0.7342	Yes
**1246**	Row 9:**0 X** vs. Row 17:**25 X**	-0.5050	-0.9823 to -0.02770	Yes
**1247**	Row 9:**0 X** vs. 17:**0 X**	-1.713	-2.190 to -1.235	Yes
**1248**	Row 9:**0 X** vs. 17:**10 X**	-1.506	-1.983 to -1.029	Yes
**1249**	Row 9:**0 X** vs. 17:**25 X**	-0.4153	-0.8422 to 0.01158	No
**1250**	Row 9:**0 X** vs. Row 19:**0 X**	-1.809	-2.286 to -1.331	Yes
**1251**	Row 9:**0 X** vs. Row 19:**10 X**	-1.509	-1.986 to -1.031	Yes
**1252**	Row 9:**0 X** vs. Row 19:**25 X**	-0.3450	-0.7719 to 0.08191	No
**1253**	Row 9:**0 X** vs. Row 20:**0 X**	-1.861	-2.338 to -1.384	Yes
**1254**	Row 9:**0 X** vs. Row 20:**10 X**	-1.402	-1.879 to -0.9247	Yes
**1255**	Row 9:**0 X** vs. Row 20:**25 X**	-0.2887	-0.7156 to 0.1382	No
**1256**	Row 9:**0 X** vs. Row 21:**0 X**	-1.218	-1.695 to -0.7407	Yes
**1257**	Row 9:**0 X** vs. Row 21:**10 X**	-0.7875	-1.265 to -0.3102	Yes
**1258**	Row 9:**0 X** vs. Row 21:**25 X**	-0.3160	-0.7429 to 0.1109	No
**1259**	Row 9:**10 X** vs. Row 9:**25 X**	0.1837	-0.2432 to 0.6106	No
**1260**	Row 9:**10 X** vs. Row 10:**0 X**	-0.2640	-0.6909 to 0.1629	No

	2way ANOVA<br>Multiple comparisons	A<br>Data Set-A<br>Y	B<br>Data Set-B<br>Y	C<br>Data Set-C<br>Y
**1261**	Row 9:**10 X** vs. Row 10:**10 X**	-0.2557	-0.6826 to 0.1712	No
**1262**	Row 9:**10 X** vs. Row 10:**25 X**	-0.08900	-0.5159 to 0.3379	No
**1263**	Row 9:**10 X** vs. Row 11:**0 X**	-1.022	-1.449 to -0.5951	Yes
**1264**	Row 9:**10 X** vs. Row 11:**10 X**	-0.3740	-0.8513 to 0.1033	No
**1265**	Row 9:**10 X** vs. Row 11:**25 X**	-0.3167	-0.7436 to 0.1102	No
**1266**	Row 9:**10 X** vs. 11:**0 X**	-1.116	-1.543 to -0.6888	Yes
**1267**	Row 9:**10 X** vs. 11:**10 X**	-0.6900	-1.167 to -0.2127	Yes
**1268**	Row 9:**10 X** vs. 11:**25 X**	-0.6207	-1.048 to -0.1938	Yes
**1269**	Row 9:**10 X** vs. Row 13:**0 X**	-1.085	-1.512 to -0.6578	Yes
**1270**	Row 9:**10 X** vs. Row 13:**10 X**	-0.7180	-1.195 to -0.2407	Yes
**1271**	Row 9:**10 X** vs. Row 13:**25 X**	-0.7267	-1.154 to -0.2998	Yes
**1272**	Row 9:**10 X** vs. Row 14:**0 X**	-1.135	-1.562 to -0.7084	Yes
**1273**	Row 9:**10 X** vs. Row 14:**10 X**	-0.9113	-1.338 to -0.4844	Yes
**1274**	Row 9:**10 X** vs. Row 14:**25 X**	-0.8713	-1.298 to -0.4444	Yes
**1275**	Row 9:**10 X** vs. 14:**0 X**	-1.729	-2.156 to -1.302	Yes
**1276**	Row 9:**10 X** vs. 14:**10 X**	-1.126	-1.603 to -0.6487	Yes
**1277**	Row 9:**10 X** vs. 14:**25 X**	-0.7620	-1.239 to -0.2847	Yes
**1278**	Row 9:**10 X** vs. Row 16:**0 X**	-1.842	-2.319 to -1.365	Yes
**1279**	Row 9:**10 X** vs. Row 16:**10 X**	-1.210	-1.687 to -0.7327	Yes
**1280**	Row 9:**10 X** vs. Row 16:**25 X**	-0.3550	-0.8323 to 0.1223	No
**1281**	Row 9:**10 X** vs. Row 17:**0 X**	-1.468	-1.945 to -0.9902	Yes
**1282**	Row 9:**10 X** vs. Row 17:**10 X**	-1.228	-1.705 to -0.7502	Yes
**1283**	Row 9:**10 X** vs. Row 17:**25 X**	-0.5210	-0.9983 to -0.04370	Yes
**1284**	Row 9:**10 X** vs. 17:**0 X**	-1.729	-2.206 to -1.251	Yes
**1285**	Row 9:**10 X** vs. 17:**10 X**	-1.522	-1.999 to -1.045	Yes
**1286**	Row 9:**10 X** vs. 17:**25 X**	-0.4313	-0.8582 to -0.004421	Yes
**1287**	Row 9:**10 X** vs. Row 19:**0 X**	-1.825	-2.302 to -1.347	Yes
**1288**	Row 9:**10 X** vs. Row 19:**10 X**	-1.525	-2.002 to -1.047	Yes
**1289**	Row 9:**10 X** vs. Row 19:**25 X**	-0.3610	-0.7879 to 0.06591	No
**1290**	Row 9:**10 X** vs. Row 20:**0 X**	-1.877	-2.354 to -1.400	Yes
**1291**	Row 9:**10 X** vs. Row 20:**10 X**	-1.418	-1.895 to -0.9407	Yes
**1292**	Row 9:**10 X** vs. Row 20:**25 X**	-0.3047	-0.7316 to 0.1222	No
**1293**	Row 9:**10 X** vs. Row 21:**0 X**	-1.234	-1.711 to -0.7567	Yes
**1294**	Row 9:**10 X** vs. Row 21:**10 X**	-0.8035	-1.281 to -0.3262	Yes
**1295**	Row 9:**10 X** vs. Row 21:**25 X**	-0.3320	-0.7589 to 0.09491	No
**1296**	Row 9:**25 X** vs. Row 10:**0 X**	-0.4477	-0.8746 to -0.02075	Yes
**1297**	Row 9:**25 X** vs. Row 10:**10 X**	-0.4393	-0.8662 to -0.01242	Yes
**1298**	Row 9:**25 X** vs. Row 10:**25 X**	-0.2727	-0.6996 to 0.1542	No
**1299**	Row 9:**25 X** vs. Row 11:**0 X**	-1.206	-1.633 to -0.7788	Yes
**1300**	Row 9:**25 X** vs. Row 11:**10 X**	-0.5577	-1.035 to -0.08036	Yes
**1301**	Row 9:**25 X** vs. Row 11:**25 X**	-0.5003	-0.9272 to -0.07342	Yes
**1302**	Row 9:**25 X** vs. 11:**0 X**	-1.299	-1.726 to -0.8724	Yes
**1303**	Row 9:**25 X** vs. 11:**10 X**	-0.8737	-1.351 to -0.3964	Yes
**1304**	Row 9:**25 X** vs. 11:**25 X**	-0.8043	-1.231 to -0.3774	Yes
**1305**	Row 9:**25 X** vs. Row 13:**0 X**	-1.268	-1.695 to -0.8414	Yes

	**2way ANOVA** Multiple comparisons	A Data Set-A Y	B Data Set-B Y	C Data Set-C Y
**1306**	Row 9:**25 X** vs. Row 13:**10 X**	-0.9017	-1.379 to -0.4244	Yes
**1307**	Row 9:**25 X** vs. Row 13:**25 X**	-0.9103	-1.337 to -0.4834	Yes
**1308**	Row 9:**25 X** vs. Row 14:**0 X**	-1.319	-1.746 to -0.8921	Yes
**1309**	Row 9:**25 X** vs. Row 14:**10 X**	-1.095	-1.522 to -0.6681	Yes
**1310**	Row 9:**25 X** vs. Row 14:**25 X**	-1.055	-1.482 to -0.6281	Yes
**1311**	Row 9:**25 X** vs. 14:**0 X**	-1.913	-2.340 to -1.486	Yes
**1312**	Row 9:**25 X** vs. 14:**10 X**	-1.310	-1.787 to -0.8324	Yes
**1313**	Row 9:**25 X** vs. 14:**25 X**	-0.9457	-1.423 to -0.4684	Yes
**1314**	Row 9:**25 X** vs. Row 16:**0 X**	-2.026	-2.503 to -1.548	Yes
**1315**	Row 9:**25 X** vs. Row 16:**10 X**	-1.394	-1.871 to -0.9164	Yes
**1316**	Row 9:**25 X** vs. Row 16:**25 X**	-0.5387	-1.016 to -0.06136	Yes
**1317**	Row 9:**25 X** vs. Row 17:**0 X**	-1.651	-2.128 to -1.174	Yes
**1318**	Row 9:**25 X** vs. Row 17:**10 X**	-1.411	-1.888 to -0.9339	Yes
**1319**	Row 9:**25 X** vs. Row 17:**25 X**	-0.7047	-1.182 to -0.2274	Yes
**1320**	Row 9:**25 X** vs. 17:**0 X**	-1.912	-2.389 to -1.435	Yes
**1321**	Row 9:**25 X** vs. 17:**10 X**	-1.706	-2.183 to -1.228	Yes
**1322**	Row 9:**25 X** vs. 17:**25 X**	-0.6150	-1.042 to -0.1881	Yes
**1323**	Row 9:**25 X** vs. Row 19:**0 X**	-2.008	-2.485 to -1.531	Yes
**1324**	Row 9:**25 X** vs. Row 19:**10 X**	-1.708	-2.185 to -1.231	Yes
**1325**	Row 9:**25 X** vs. Row 19:**25 X**	-0.5447	-0.9716 to -0.1178	Yes
**1326**	Row 9:**25 X** vs. Row 20:**0 X**	-2.061	-2.538 to -1.583	Yes
**1327**	Row 9:**25 X** vs. Row 20:**10 X**	-1.602	-2.079 to -1.124	Yes
**1328**	Row 9:**25 X** vs. Row 20:**25 X**	-0.4883	-0.9152 to -0.06142	Yes
**1329**	Row 9:**25 X** vs. Row 21:**0 X**	-1.418	-1.895 to -0.9404	Yes
**1330**	Row 9:**25 X** vs. Row 21:**10 X**	-0.9872	-1.464 to -0.5099	Yes
**1331**	Row 9:**25 X** vs. Row 21:**25 X**	-0.5157	-0.9426 to -0.08875	Yes
**1332**	Row 10:**0 X** vs. Row 10:**10 X**	0.008333	-0.4186 to 0.4352	No
**1333**	Row 10:**0 X** vs. Row 10:**25 X**	0.1750	-0.2519 to 0.6019	No
**1334**	Row 10:**0 X** vs. Row 11:**0 X**	-0.7580	-1.185 to -0.3311	Yes
**1335**	Row 10:**0 X** vs. Row 11:**10 X**	-0.1100	-0.5873 to 0.3673	No
**1336**	Row 10:**0 X** vs. Row 11:**25 X**	-0.05267	-0.4796 to 0.3742	No
**1337**	Row 10:**0 X** vs. 11:**0 X**	-0.8517	-1.279 to -0.4248	Yes
**1338**	Row 10:**0 X** vs. 11:**10 X**	-0.4260	-0.9033 to 0.05130	No
**1339**	Row 10:**0 X** vs. 11:**25 X**	-0.3567	-0.7836 to 0.07025	No
**1340**	Row 10:**0 X** vs. Row 13:**0 X**	-0.8207	-1.248 to -0.3938	Yes
**1341**	Row 10:**0 X** vs. Row 13:**10 X**	-0.4540	-0.9313 to 0.02330	No
**1342**	Row 10:**0 X** vs. Row 13:**25 X**	-0.4627	-0.8896 to -0.03575	Yes
**1343**	Row 10:**0 X** vs. Row 14:**0 X**	-0.8713	-1.298 to -0.4444	Yes
**1344**	Row 10:**0 X** vs. Row 14:**10 X**	-0.6473	-1.074 to -0.2204	Yes
**1345**	Row 10:**0 X** vs. Row 14:**25 X**	-0.6073	-1.034 to -0.1804	Yes
**1346**	Row 10:**0 X** vs. 14:**0 X**	-1.465	-1.892 to -1.038	Yes
**1347**	Row 10:**0 X** vs. 14:**10 X**	-0.8620	-1.339 to -0.3847	Yes
**1348**	Row 10:**0 X** vs. 14:**25 X**	-0.4980	-0.9753 to -0.02070	Yes
**1349**	Row 10:**0 X** vs. Row 16:**0 X**	-1.578	-2.055 to -1.101	Yes
**1350**	Row 10:**0 X** vs. Row 16:**10 X**	-0.9460	-1.423 to -0.4687	Yes

	2way ANOVA<br>Multiple comparisons	A<br>Data Set-A<br>Y	B<br>Data Set-B<br>Y	C<br>Data Set-C<br>Y
1351	Row 10:**0 X** vs. Row 16:**25 X**	-0.0910	-0.5683 to 0.3863	No
1352	Row 10:**0 X** vs. Row 17:**0 X**	-1.204	-1.681 to -0.7262	Yes
1353	Row 10:**0 X** vs. Row 17:**10 X**	-0.9635	-1.441 to -0.4862	Yes
1354	Row 10:**0 X** vs. Row 17:**25 X**	-0.2570	-0.7343 to 0.2203	No
1355	Row 10:**0 X** vs. 17:**0 X**	-1.465	-1.942 to -0.9872	Yes
1356	Row 10:**0 X** vs. 17:**10 X**	-1.258	-1.735 to -0.7807	Yes
1357	Row 10:**0 X** vs. 17:**25 X**	-0.1673	-0.5942 to 0.2596	No
1358	Row 10:**0 X** vs. Row 19:**0 X**	-1.561	-2.038 to -1.083	Yes
1359	Row 10:**0 X** vs. Row 19:**10 X**	-1.261	-1.738 to -0.7832	Yes
1360	Row 10:**0 X** vs. Row 19:**25 X**	-0.09700	-0.5239 to 0.3299	No
1361	Row 10:**0 X** vs. Row 20:**0 X**	-1.613	-2.090 to -1.136	Yes
1362	Row 10:**0 X** vs. Row 20:**10 X**	-1.154	-1.631 to -0.6767	Yes
1363	Row 10:**0 X** vs. Row 20:**25 X**	-0.04067	-0.4676 to 0.3862	No
1364	Row 10:**0 X** vs. Row 21:**0 X**	-0.9700	-1.447 to -0.4927	Yes
1365	Row 10:**0 X** vs. Row 21:**10 X**	-0.5395	-1.017 to -0.06220	Yes
1366	Row 10:**0 X** vs. Row 21:**25 X**	-0.06800	-0.4949 to 0.3589	No
1367	Row 10:**10 X** vs. Row 10:**25 X**	0.1667	-0.2602 to 0.5936	No
1368	Row 10:**10 X** vs. Row 11:**0 X**	-0.7663	-1.193 to -0.3394	Yes
1369	Row 10:**10 X** vs. Row 11:**10 X**	-0.1183	-0.5956 to 0.3590	No
1370	Row 10:**10 X** vs. Row 11:**25 X**	-0.06100	-0.4879 to 0.3659	No
1371	Row 10:**10 X** vs. 11:**0 X**	-0.8600	-1.287 to -0.4331	Yes
1372	Row 10:**10 X** vs. 11:**10 X**	-0.4343	-0.9116 to 0.04297	No
1373	Row 10:**10 X** vs. 11:**25 X**	-0.3650	-0.7919 to 0.06191	No
1374	Row 10:**10 X** vs. Row 13:**0 X**	-0.8290	-1.256 to -0.4021	Yes
1375	Row 10:**10 X** vs. Row 13:**10 X**	-0.4623	-0.9396 to 0.01497	No
1376	Row 10:**10 X** vs. Row 13:**25 X**	-0.4710	-0.8979 to -0.04409	Yes
1377	Row 10:**10 X** vs. Row 14:**0 X**	-0.8797	-1.307 to -0.4528	Yes
1378	Row 10:**10 X** vs. Row 14:**10 X**	-0.6557	-1.083 to -0.2288	Yes
1379	Row 10:**10 X** vs. Row 14:**25 X**	-0.6157	-1.043 to -0.1888	Yes
1380	Row 10:**10 X** vs. 14:**0 X**	-1.474	-1.901 to -1.047	Yes
1381	Row 10:**10 X** vs. 14:**10 X**	-0.8703	-1.348 to -0.3930	Yes
1382	Row 10:**10 X** vs. 14:**25 X**	-0.5063	-0.9836 to -0.02903	Yes
1383	Row 10:**10 X** vs. Row 16:**0 X**	-1.586	-2.064 to -1.109	Yes
1384	Row 10:**10 X** vs. Row 16:**10 X**	-0.9543	-1.432 to -0.4770	Yes
1385	Row 10:**10 X** vs. Row 16:**25 X**	-0.09933	-0.5766 to 0.3780	No
1386	Row 10:**10 X** vs. Row 17:**0 X**	-1.212	-1.689 to -0.7345	Yes
1387	Row 10:**10 X** vs. Row 17:**10 X**	-0.9718	-1.449 to -0.4945	Yes
1388	Row 10:**10 X** vs. Row 17:**25 X**	-0.2653	-0.7426 to 0.2120	No
1389	Row 10:**10 X** vs. 17:**0 X**	-1.473	-1.950 to -0.9955	Yes
1390	Row 10:**10 X** vs. 17:**10 X**	-1.266	-1.744 to -0.7890	Yes
1391	Row 10:**10 X** vs. 17:**25 X**	-0.1757	-0.6026 to 0.2512	No
1392	Row 10:**10 X** vs. Row 19:**0 X**	-1.569	-2.046 to -1.092	Yes
1393	Row 10:**10 X** vs. Row 19:**10 X**	-1.269	-1.746 to -0.7915	Yes
1394	Row 10:**10 X** vs. Row 19:**25 X**	-0.1053	-0.5322 to 0.3216	No
1395	Row 10:**10 X** vs. Row 20:**0 X**	-1.621	-2.099 to -1.144	Yes

	2way ANOVA<br>Multiple comparisons	A<br>Data Set-A<br>Y	B<br>Data Set-B<br>Y	C<br>Data Set-C<br>Y
1396	Row 10:**10 X** vs. Row 20:**10 X**	-1.162	-1.640 to -0.6850	Yes
1397	Row 10:**10 X** vs. Row 20:**25 X**	-0.04900	-0.4759 to 0.3779	No
1398	Row 10:**10 X** vs. Row 21:**0 X**	-0.9783	-1.456 to -0.5010	Yes
1399	Row 10:**10 X** vs. Row 21:**10 X**	-0.5478	-1.025 to -0.07053	Yes
1400	Row 10:**10 X** vs. Row 21:**25 X**	-0.07633	-0.5032 to 0.3506	No
1401	Row 10:**25 X** vs. Row 11:**0 X**	-0.9330	-1.360 to -0.5061	Yes
1402	Row 10:**25 X** vs. Row 11:**10 X**	-0.2850	-0.7623 to 0.1923	No
1403	Row 10:**25 X** vs. Row 11:**25 X**	-0.2277	-0.6546 to 0.1992	No
1404	Row 10:**25 X** vs. 11:**0 X**	-1.027	-1.454 to -0.5998	Yes
1405	Row 10:**25 X** vs. 11:**10 X**	-0.6010	-1.078 to -0.1237	Yes
1406	Row 10:**25 X** vs. 11:**25 X**	-0.5317	-0.9586 to -0.1048	Yes
1407	Row 10:**25 X** vs. Row 13:**0 X**	-0.9957	-1.423 to -0.5688	Yes
1408	Row 10:**25 X** vs. Row 13:**10 X**	-0.6290	-1.106 to -0.1517	Yes
1409	Row 10:**25 X** vs. Row 13:**25 X**	-0.6377	-1.065 to -0.2108	Yes
1410	Row 10:**25 X** vs. Row 14:**0 X**	-1.046	-1.473 to -0.6194	Yes
1411	Row 10:**25 X** vs. Row 14:**10 X**	-0.8223	-1.249 to -0.3954	Yes
1412	Row 10:**25 X** vs. Row 14:**25 X**	-0.7823	-1.209 to -0.3554	Yes
1413	Row 10:**25 X** vs. 14:**0 X**	-1.640	-2.067 to -1.213	Yes
1414	Row 10:**25 X** vs. 14:**10 X**	-1.037	-1.514 to -0.5597	Yes
1415	Row 10:**25 X** vs. 14:**25 X**	-0.6730	-1.150 to -0.1957	Yes
1416	Row 10:**25 X** vs. Row 16:**0 X**	-1.753	-2.230 to -1.276	Yes
1417	Row 10:**25 X** vs. Row 16:**10 X**	-1.121	-1.598 to -0.6437	Yes
1418	Row 10:**25 X** vs. Row 16:**25 X**	-0.2660	-0.7433 to 0.2113	No
1419	Row 10:**25 X** vs. Row 17:**0 X**	-1.379	-1.856 to -0.9012	Yes
1420	Row 10:**25 X** vs. Row 17:**10 X**	-1.139	-1.616 to -0.6612	Yes
1421	Row 10:**25 X** vs. Row 17:**25 X**	-0.4320	-0.9093 to 0.04530	No
1422	Row 10:**25 X** vs. 17:**0 X**	-1.640	-2.117 to -1.162	Yes
1423	Row 10:**25 X** vs. 17:**10 X**	-1.433	-1.910 to -0.9557	Yes
1424	Row 10:**25 X** vs. 17:**25 X**	-0.3423	-0.7692 to 0.08458	No
1425	Row 10:**25 X** vs. Row 19:**0 X**	-1.736	-2.213 to -1.258	Yes
1426	Row 10:**25 X** vs. Row 19:**10 X**	-1.436	-1.913 to -0.9582	Yes
1427	Row 10:**25 X** vs. Row 19:**25 X**	-0.2720	-0.6989 to 0.1549	No
1428	Row 10:**25 X** vs. Row 20:**0 X**	-1.788	-2.265 to -1.311	Yes
1429	Row 10:**25 X** vs. Row 20:**10 X**	-1.329	-1.806 to -0.8517	Yes
1430	Row 10:**25 X** vs. Row 20:**25 X**	-0.2157	-0.6426 to 0.2112	No
1431	Row 10:**25 X** vs. Row 21:**0 X**	-1.145	-1.622 to -0.6677	Yes
1432	Row 10:**25 X** vs. Row 21:**10 X**	-0.7145	-1.192 to -0.2372	Yes
1433	Row 10:**25 X** vs. Row 21:**25 X**	-0.2430	-0.6699 to 0.1839	No
1434	Row 11:**0 X** vs. Row 11:**10 X**	0.6480	0.1707 to 1.125	Yes
1435	Row 11:**0 X** vs. Row 11:**25 X**	0.7053	0.2784 to 1.132	Yes
1436	Row 11:**0 X** vs. 11:**0 X**	-0.09367	-0.5206 to 0.3332	No
1437	Row 11:**0 X** vs. 11:**10 X**	0.3320	-0.1453 to 0.8093	No
1438	Row 11:**0 X** vs. 11:**25 X**	0.4013	-0.02558 to 0.8282	No
1439	Row 11:**0 X** vs. Row 13:**0 X**	-0.06267	-0.4896 to 0.3642	No
1440	Row 11:**0 X** vs. Row 13:**10 X**	0.3040	-0.1733 to 0.7813	No

	2way ANOVA<br>Multiple comparisons	A<br>Data Set-A<br>Y	B<br>Data Set-B<br>Y	C<br>Data Set-C<br>Y
**1441**	Row 11:**0 X** vs. Row 13:**25 X**	0.2953	-0.1316 to 0.7222	No
**1442**	Row 11:**0 X** vs. Row 14:**0 X**	-0.1133	-0.5402 to 0.3136	No
**1443**	Row 11:**0 X** vs. Row 14:**10 X**	0.1107	-0.3162 to 0.5376	No
**1444**	Row 11:**0 X** vs. Row 14:**25 X**	0.1507	-0.2762 to 0.5776	No
**1445**	Row 11:**0 X** vs. 14:**0 X**	-0.7073	-1.134 to -0.2804	Yes
**1446**	Row 11:**0 X** vs. 14:**10 X**	-0.1040	-0.5813 to 0.3733	No
**1447**	Row 11:**0 X** vs. 14:**25 X**	0.2600	-0.2173 to 0.7373	No
**1448**	Row 11:**0 X** vs. Row 16:**0 X**	-0.8200	-1.297 to -0.3427	Yes
**1449**	Row 11:**0 X** vs. Row 16:**10 X**	-0.1880	-0.6653 to 0.2893	No
**1450**	Row 11:**0 X** vs. Row 16:**25 X**	0.6670	0.1897 to 1.144	Yes
**1451**	Row 11:**0 X** vs. Row 17:**0 X**	-0.4455	-0.9228 to 0.03180	No
**1452**	Row 11:**0 X** vs. Row 17:**10 X**	-0.2055	-0.6828 to 0.2718	No
**1453**	Row 11:**0 X** vs. Row 17:**25 X**	0.5010	0.02370 to 0.9783	Yes
**1454**	Row 11:**0 X** vs. 17:**0 X**	-0.7065	-1.184 to -0.2292	Yes
**1455**	Row 11:**0 X** vs. 17:**10 X**	-0.5000	-0.9773 to -0.02270	Yes
**1456**	Row 11:**0 X** vs. 17:**25 X**	0.5907	0.1638 to 1.018	Yes
**1457**	Row 11:**0 X** vs. Row 19:**0 X**	-0.8025	-1.280 to -0.3252	Yes
**1458**	Row 11:**0 X** vs. Row 19:**10 X**	-0.5025	-0.9798 to -0.02520	Yes
**1459**	Row 11:**0 X** vs. Row 19:**25 X**	0.6610	0.2341 to 1.088	Yes
**1460**	Row 11:**0 X** vs. Row 20:**0 X**	-0.8550	-1.332 to -0.3777	Yes
**1461**	Row 11:**0 X** vs. Row 20:**10 X**	-0.3960	-0.8733 to 0.08130	No
**1462**	Row 11:**0 X** vs. Row 20:**25 X**	0.7173	0.2904 to 1.144	Yes
**1463**	Row 11:**0 X** vs. Row 21:**0 X**	-0.2120	-0.6893 to 0.2653	No
**1464**	Row 11:**0 X** vs. Row 21:**10 X**	0.2185	-0.2588 to 0.6958	No
**1465**	Row 11:**0 X** vs. Row 21:**25 X**	0.6900	0.2631 to 1.117	Yes
**1466**	Row 11:**10 X** vs. Row 11:**25 X**	0.05733	-0.4200 to 0.5346	No
**1467**	Row 11:**10 X** vs. 11:**0 X**	-0.7417	-1.219 to -0.2644	Yes
**1468**	Row 11:**10 X** vs. 11:**10 X**	-0.3160	-0.8389 to 0.2069	No
**1469**	Row 11:**10 X** vs. 11:**25 X**	-0.2467	-0.7240 to 0.2306	No
**1470**	Row 11:**10 X** vs. Row 13:**0 X**	-0.7107	-1.188 to -0.2334	Yes
**1471**	Row 11:**10 X** vs. Row 13:**10 X**	-0.3440	-0.8669 to 0.1789	No
**1472**	Row 11:**10 X** vs. Row 13:**25 X**	-0.3527	-0.8300 to 0.1246	No
**1473**	Row 11:**10 X** vs. Row 14:**0 X**	-0.7613	-1.239 to -0.2840	Yes
**1474**	Row 11:**10 X** vs. Row 14:**10 X**	-0.5373	-1.015 to -0.06003	Yes
**1475**	Row 11:**10 X** vs. Row 14:**25 X**	-0.4973	-0.9746 to -0.02003	Yes
**1476**	Row 11:**10 X** vs. 14:**0 X**	-1.355	-1.833 to -0.8780	Yes
**1477**	Row 11:**10 X** vs. 14:**10 X**	-0.7520	-1.275 to -0.2291	Yes
**1478**	Row 11:**10 X** vs. 14:**25 X**	-0.3880	-0.9109 to 0.1349	No
**1479**	Row 11:**10 X** vs. Row 16:**0 X**	-1.468	-1.991 to -0.9451	Yes
**1480**	Row 11:**10 X** vs. Row 16:**10 X**	-0.8360	-1.359 to -0.3131	Yes
**1481**	Row 11:**10 X** vs. Row 16:**25 X**	0.01900	-0.5039 to 0.5419	No
**1482**	Row 11:**10 X** vs. Row 17:**0 X**	-1.094	-1.616 to -0.5706	Yes
**1483**	Row 11:**10 X** vs. Row 17:**10 X**	-0.8535	-1.376 to -0.3306	Yes
**1484**	Row 11:**10 X** vs. Row 17:**25 X**	-0.1470	-0.6699 to 0.3759	No
**1485**	Row 11:**10 X** vs. 17:**0 X**	-1.355	-1.877 to -0.8316	Yes

	2way ANOVA<br>Multiple comparisons	A<br>Data Set-A<br>Y	B<br>Data Set-B<br>Y	C<br>Data Set-C<br>Y
**1486**	Row 11:**10 X** vs. 17:**10 X**	-1.148	-1.671 to -0.6251	Yes
**1487**	Row 11:**10 X** vs. 17:**25 X**	-0.05733	-0.5346 to 0.4200	No
**1488**	Row 11:**10 X** vs. Row 19:**0 X**	-1.451	-1.973 to -0.9276	Yes
**1489**	Row 11:**10 X** vs. Row 19:**10 X**	-1.151	-1.673 to -0.6276	Yes
**1490**	Row 11:**10 X** vs. Row 19:**25 X**	0.01300	-0.4643 to 0.4903	No
**1491**	Row 11:**10 X** vs. Row 20:**0 X**	-1.503	-2.026 to -0.9801	Yes
**1492**	Row 11:**10 X** vs. Row 20:**10 X**	-1.044	-1.567 to -0.5211	Yes
**1493**	Row 11:**10 X** vs. Row 20:**25 X**	0.06933	-0.4080 to 0.5466	No
**1494**	Row 11:**10 X** vs. Row 21:**0 X**	-0.8600	-1.383 to -0.3371	Yes
**1495**	Row 11:**10 X** vs. Row 21:**10 X**	-0.4295	-0.9524 to 0.09336	No
**1496**	Row 11:**10 X** vs. Row 21:**25 X**	0.04200	-0.4353 to 0.5193	No
**1497**	Row 11:**25 X** vs. 11:**0 X**	-0.7990	-1.226 to -0.3721	Yes
**1498**	Row 11:**25 X** vs. 11:**10 X**	-0.3733	-0.8506 to 0.1040	No
**1499**	Row 11:**25 X** vs. 11:**25 X**	-0.3040	-0.7309 to 0.1229	No
**1500**	Row 11:**25 X** vs. Row 13:**0 X**	-0.7680	-1.195 to -0.3411	Yes
**1501**	Row 11:**25 X** vs. Row 13:**10 X**	-0.4013	-0.8786 to 0.07597	No
**1502**	Row 11:**25 X** vs. Row 13:**25 X**	-0.4100	-0.8369 to 0.01691	No
**1503**	Row 11:**25 X** vs. Row 14:**0 X**	-0.8187	-1.246 to -0.3918	Yes
**1504**	Row 11:**25 X** vs. Row 14:**10 X**	-0.5947	-1.022 to -0.1678	Yes
**1505**	Row 11:**25 X** vs. Row 14:**25 X**	-0.5547	-0.9816 to -0.1278	Yes
**1506**	Row 11:**25 X** vs. 14:**0 X**	-1.413	-1.840 to -0.9858	Yes
**1507**	Row 11:**25 X** vs. 14:**10 X**	-0.8093	-1.287 to -0.3320	Yes
**1508**	Row 11:**25 X** vs. 14:**25 X**	-0.4453	-0.9226 to 0.03197	No
**1509**	Row 11:**25 X** vs. Row 16:**0 X**	-1.525	-2.003 to -1.048	Yes
**1510**	Row 11:**25 X** vs. Row 16:**10 X**	-0.8933	-1.371 to -0.4160	Yes
**1511**	Row 11:**25 X** vs. Row 16:**25 X**	-0.03833	-0.5156 to 0.4390	No
**1512**	Row 11:**25 X** vs. Row 17:**0 X**	-1.151	-1.628 to -0.6735	Yes
**1513**	Row 11:**25 X** vs. Row 17:**10 X**	-0.9108	-1.388 to -0.4335	Yes
**1514**	Row 11:**25 X** vs. Row 17:**25 X**	-0.2043	-0.6816 to 0.2730	No
**1515**	Row 11:**25 X** vs. 17:**0 X**	-1.412	-1.889 to -0.9345	Yes
**1516**	Row 11:**25 X** vs. 17:**10 X**	-1.205	-1.683 to -0.7280	Yes
**1517**	Row 11:**25 X** vs. 17:**25 X**	-0.1147	-0.5416 to 0.3122	No
**1518**	Row 11:**25 X** vs. Row 19:**0 X**	-1.508	-1.985 to -1.031	Yes
**1519**	Row 11:**25 X** vs. Row 19:**10 X**	-1.208	-1.685 to -0.7305	Yes
**1520**	Row 11:**25 X** vs. Row 19:**25 X**	-0.04433	-0.4712 to 0.3826	No
**1521**	Row 11:**25 X** vs. Row 20:**0 X**	-1.560	-2.038 to -1.083	Yes
**1522**	Row 11:**25 X** vs. Row 20:**10 X**	-1.101	-1.579 to -0.6240	Yes
**1523**	Row 11:**25 X** vs. Row 20:**25 X**	0.01200	-0.4149 to 0.4389	No
**1524**	Row 11:**25 X** vs. Row 21:**0 X**	-0.9173	-1.395 to -0.4400	Yes
**1525**	Row 11:**25 X** vs. Row 21:**10 X**	-0.4868	-0.9641 to -0.009531	Yes
**1526**	Row 11:**25 X** vs. Row 21:**25 X**	-0.01533	-0.4422 to 0.4116	No
**1527**	11:**0 X** vs. 11:**10 X**	0.4257	-0.05164 to 0.9030	No
**1528**	11:**0 X** vs. 11:**25 X**	0.4950	0.06809 to 0.9219	Yes
**1529**	11:**0 X** vs. Row 13:**0 X**	0.03100	-0.3959 to 0.4579	No
**1530**	11:**0 X** vs. Row 13:**10 X**	0.3977	-0.07964 to 0.8750	No

	2way ANOVA Multiple comparisons	A Data Set-A Y	B Data Set-B Y	C Data Set-C Y
1531	11:**0 X** vs. Row 13:**25 X**	0.3890	-0.03791 to 0.8159	No
1532	11:**0 X** vs. Row 14:**0 X**	-0.01967	-0.4466 to 0.4072	No
1533	11:**0 X** vs. Row 14:**10 X**	0.2043	-0.2226 to 0.6312	No
1534	11:**0 X** vs. Row 14:**25 X**	0.2443	-0.1826 to 0.6712	No
1535	11:**0 X** vs. 14:**0 X**	-0.6137	-1.041 to -0.1868	Yes
1536	11:**0 X** vs. 14:**10 X**	-0.01033	-0.4876 to 0.4670	No
1537	11:**0 X** vs. 14:**25 X**	0.3537	-0.1236 to 0.8310	No
1538	11:**0 X** vs. Row 16:**0 X**	-0.7263	-1.204 to -0.2490	Yes
1539	11:**0 X** vs. Row 16:**10 X**	-0.09433	-0.5716 to 0.3830	No
1540	11:**0 X** vs. Row 16:**25 X**	0.7607	0.2834 to 1.238	Yes
1541	11:**0 X** vs. Row 17:**0 X**	-0.3518	-0.8291 to 0.1255	No
1542	11:**0 X** vs. Row 17:**10 X**	-0.1118	-0.5891 to 0.3655	No
1543	11:**0 X** vs. Row 17:**25 X**	0.5947	0.1174 to 1.072	Yes
1544	11:**0 X** vs. 17:**0 X**	-0.6128	-1.090 to -0.1355	Yes
1545	11:**0 X** vs. 17:**10 X**	-0.4063	-0.8836 to 0.07097	No
1546	11:**0 X** vs. 17:**25 X**	0.6843	0.2574 to 1.111	Yes
1547	11:**0 X** vs. Row 19:**0 X**	-0.7088	-1.186 to -0.2315	Yes
1548	11:**0 X** vs. Row 19:**10 X**	-0.4088	-0.8861 to 0.06847	No
1549	11:**0 X** vs. Row 19:**25 X**	0.7547	0.3278 to 1.182	Yes
1550	11:**0 X** vs. Row 20:**0 X**	-0.7613	-1.239 to -0.2840	Yes
1551	11:**0 X** vs. Row 20:**10 X**	-0.3023	-0.7796 to 0.1750	No
1552	11:**0 X** vs. Row 20:**25 X**	0.8110	0.3841 to 1.238	Yes
1553	11:**0 X** vs. Row 21:**0 X**	-0.1183	-0.5956 to 0.3590	No
1554	11:**0 X** vs. Row 21:**10 X**	0.3122	-0.1651 to 0.7895	No
1555	11:**0 X** vs. Row 21:**25 X**	0.7837	0.3568 to 1.211	Yes
1556	11:**10 X** vs. 11:**25 X**	0.06933	-0.4080 to 0.5466	No
1557	11:**10 X** vs. Row 13:**0 X**	-0.3947	-0.8720 to 0.08264	No
1558	11:**10 X** vs. Row 13:**10 X**	-0.02800	-0.5509 to 0.4949	No
1559	11:**10 X** vs. Row 13:**25 X**	-0.03667	-0.5140 to 0.4406	No
1560	11:**10 X** vs. Row 14:**0 X**	-0.4453	-0.9226 to 0.03197	No
1561	11:**10 X** vs. Row 14:**10 X**	-0.2213	-0.6986 to 0.2560	No
1562	11:**10 X** vs. Row 14:**25 X**	-0.1813	-0.6586 to 0.2960	No
1563	11:**10 X** vs. 14:**0 X**	-1.039	-1.517 to -0.5620	Yes
1564	11:**10 X** vs. 14:**10 X**	-0.4360	-0.9589 to 0.08686	No
1565	11:**10 X** vs. 14:**25 X**	-0.07200	-0.5949 to 0.4509	No
1566	11:**10 X** vs. Row 16:**0 X**	-1.152	-1.675 to -0.6291	Yes
1567	11:**10 X** vs. Row 16:**10 X**	-0.5200	-1.043 to 0.002859	No
1568	11:**10 X** vs. Row 16:**25 X**	0.3350	-0.1879 to 0.8579	No
1569	11:**10 X** vs. Row 17:**0 X**	-0.7775	-1.300 to -0.2546	Yes
1570	11:**10 X** vs. Row 17:**10 X**	-0.5375	-1.060 to -0.01464	Yes
1571	11:**10 X** vs. Row 17:**25 X**	0.1690	-0.3539 to 0.6919	No
1572	11:**10 X** vs. 17:**0 X**	-1.039	-1.561 to -0.5156	Yes
1573	11:**10 X** vs. 17:**10 X**	-0.8320	-1.355 to -0.3091	Yes
1574	11:**10 X** vs. 17:**25 X**	0.2587	-0.2186 to 0.7360	No
1575	11:**10 X** vs. Row 19:**0 X**	-1.135	-1.657 to -0.6116	Yes

	2way ANOVA Multiple comparisons	A Data Set-A Y	B Data Set-B Y	C Data Set-C Y
**1576**	11:**10 X** vs. Row 19:**10 X**	-0.8345	-1.357 to -0.3116	Yes
**1577**	11:**10 X** vs. Row 19:**25 X**	0.3290	-0.1483 to 0.8063	No
**1578**	11:**10 X** vs. Row 20:**0 X**	-1.187	-1.710 to -0.6641	Yes
**1579**	11:**10 X** vs. Row 20:**10 X**	-0.7280	-1.251 to -0.2051	Yes
**1580**	11:**10 X** vs. Row 20:**25 X**	0.3853	-0.09197 to 0.8626	No
**1581**	11:**10 X** vs. Row 21:**0 X**	-0.5440	-1.067 to -0.02114	Yes
**1582**	11:**10 X** vs. Row 21:**10 X**	-0.1135	-0.6364 to 0.4094	No
**1583**	11:**10 X** vs. Row 21:**25 X**	0.3580	-0.1193 to 0.8353	No
**1584**	11:**25 X** vs. Row 13:**0 X**	-0.4640	-0.8909 to -0.03709	Yes
**1585**	11:**25 X** vs. Row 13:**10 X**	-0.09733	-0.5746 to 0.3800	No
**1586**	11:**25 X** vs. Row 13:**25 X**	-0.1060	-0.5329 to 0.3209	No
**1587**	11:**25 X** vs. Row 14:**0 X**	-0.5147	-0.9416 to -0.08775	Yes
**1588**	11:**25 X** vs. Row 14:**10 X**	-0.2907	-0.7176 to 0.1362	No
**1589**	11:**25 X** vs. Row 14:**25 X**	-0.2507	-0.6776 to 0.1762	No
**1590**	11:**25 X** vs. 14:**0 X**	-1.109	-1.536 to -0.6818	Yes
**1591**	11:**25 X** vs. 14:**10 X**	-0.5053	-0.9826 to -0.02803	Yes
**1592**	11:**25 X** vs. 14:**25 X**	-0.1413	-0.6186 to 0.3360	No
**1593**	11:**25 X** vs. Row 16:**0 X**	-1.221	-1.699 to -0.7440	Yes
**1594**	11:**25 X** vs. Row 16:**10 X**	-0.5893	-1.067 to -0.1120	Yes
**1595**	11:**25 X** vs. Row 16:**25 X**	0.2657	-0.2116 to 0.7430	No
**1596**	11:**25 X** vs. Row 17:**0 X**	-0.8468	-1.324 to -0.3695	Yes
**1597**	11:**25 X** vs. Row 17:**10 X**	-0.6068	-1.084 to -0.1295	Yes
**1598**	11:**25 X** vs. Row 17:**25 X**	0.09967	-0.3776 to 0.5770	No
**1599**	11:**25 X** vs. 17:**0 X**	-1.108	-1.585 to -0.6305	Yes
**1600**	11:**25 X** vs. 17:**10 X**	-0.9013	-1.379 to -0.4240	Yes
**1601**	11:**25 X** vs. 17:**25 X**	0.1893	-0.2376 to 0.6162	No
**1602**	11:**25 X** vs. Row 19:**0 X**	-1.204	-1.681 to -0.7265	Yes
**1603**	11:**25 X** vs. Row 19:**10 X**	-0.9038	-1.381 to -0.4265	Yes
**1604**	11:**25 X** vs. Row 19:**25 X**	0.2597	-0.1672 to 0.6866	No
**1605**	11:**25 X** vs. Row 20:**0 X**	-1.256	-1.734 to -0.7790	Yes
**1606**	11:**25 X** vs. Row 20:**10 X**	-0.7973	-1.275 to -0.3200	Yes
**1607**	11:**25 X** vs. Row 20:**25 X**	0.3160	-0.1109 to 0.7429	No
**1608**	11:**25 X** vs. Row 21:**0 X**	-0.6133	-1.091 to -0.1360	Yes
**1609**	11:**25 X** vs. Row 21:**10 X**	-0.1828	-0.6601 to 0.2945	No
**1610**	11:**25 X** vs. Row 21:**25 X**	0.2887	-0.1382 to 0.7156	No
**1611**	Row 13:**0 X** vs. Row 13:**10 X**	0.3667	-0.1106 to 0.8440	No
**1612**	Row 13:**0 X** vs. Row 13:**25 X**	0.3580	-0.06891 to 0.7849	No
**1613**	Row 13:**0 X** vs. Row 14:**0 X**	-0.05067	-0.4776 to 0.3762	No
**1614**	Row 13:**0 X** vs. Row 14:**10 X**	0.1733	-0.2536 to 0.6002	No
**1615**	Row 13:**0 X** vs. Row 14:**25 X**	0.2133	-0.2136 to 0.6402	No
**1616**	Row 13:**0 X** vs. 14:**0 X**	-0.6447	-1.072 to -0.2178	Yes
**1617**	Row 13:**0 X** vs. 14:**10 X**	-0.04133	-0.5186 to 0.4360	No
**1618**	Row 13:**0 X** vs. 14:**25 X**	0.3227	-0.1546 to 0.8000	No
**1619**	Row 13:**0 X** vs. Row 16:**0 X**	-0.7573	-1.235 to -0.2800	Yes
**1620**	Row 13:**0 X** vs. Row 16:**10 X**	-0.1253	-0.6026 to 0.3520	No

	2way ANOVA<br>Multiple comparisons	A<br>Data Set-A<br>Y	B<br>Data Set-B<br>Y	C<br>Data Set-C<br>Y
**1621**	Row 13:**0 X** vs. Row 16:**25 X**	0.7297	0.2524 to 1.207	Yes
**1622**	Row 13:**0 X** vs. Row 17:**0 X**	-0.3828	-0.8601 to 0.09447	No
**1623**	Row 13:**0 X** vs. Row 17:**10 X**	-0.1428	-0.6201 to 0.3345	No
**1624**	Row 13:**0 X** vs. Row 17:**25 X**	0.5637	0.08636 to 1.041	Yes
**1625**	Row 13:**0 X** vs. 17:**0 X**	-0.6438	-1.121 to -0.1665	Yes
**1626**	Row 13:**0 X** vs. 17:**10 X**	-0.4373	-0.9146 to 0.03997	No
**1627**	Row 13:**0 X** vs. 17:**25 X**	0.6533	0.2264 to 1.080	Yes
**1628**	Row 13:**0 X** vs. Row 19:**0 X**	-0.7398	-1.217 to -0.2625	Yes
**1629**	Row 13:**0 X** vs. Row 19:**10 X**	-0.4398	-0.9171 to 0.03747	No
**1630**	Row 13:**0 X** vs. Row 19:**25 X**	0.7237	0.2968 to 1.151	Yes
**1631**	Row 13:**0 X** vs. Row 20:**0 X**	-0.7923	-1.270 to -0.3150	Yes
**1632**	Row 13:**0 X** vs. Row 20:**10 X**	-0.3333	-0.8106 to 0.1440	No
**1633**	Row 13:**0 X** vs. Row 20:**25 X**	0.7800	0.3531 to 1.207	Yes
**1634**	Row 13:**0 X** vs. Row 21:**0 X**	-0.1493	-0.6266 to 0.3280	No
**1635**	Row 13:**0 X** vs. Row 21:**10 X**	0.2812	-0.1961 to 0.7585	No
**1636**	Row 13:**0 X** vs. Row 21:**25 X**	0.7527	0.3258 to 1.180	Yes
**1637**	Row 13:**10 X** vs. Row 13:**25 X**	-0.008667	-0.4860 to 0.4686	No
**1638**	Row 13:**10 X** vs. Row 14:**0 X**	-0.4173	-0.8946 to 0.05997	No
**1639**	Row 13:**10 X** vs. Row 14:**10 X**	-0.1933	-0.6706 to 0.2840	No
**1640**	Row 13:**10 X** vs. Row 14:**25 X**	-0.1533	-0.6306 to 0.3240	No
**1641**	Row 13:**10 X** vs. 14:**0 X**	-1.011	-1.489 to -0.5340	Yes
**1642**	Row 13:**10 X** vs. 14:**10 X**	-0.4080	-0.9309 to 0.1149	No
**1643**	Row 13:**10 X** vs. 14:**25 X**	-0.04400	-0.5669 to 0.4789	No
**1644**	Row 13:**10 X** vs. Row 16:**0 X**	-1.124	-1.647 to -0.6011	Yes
**1645**	Row 13:**10 X** vs. Row 16:**10 X**	-0.4920	-1.015 to 0.03086	No
**1646**	Row 13:**10 X** vs. Row 16:**25 X**	0.3630	-0.1599 to 0.8859	No
**1647**	Row 13:**10 X** vs. Row 17:**0 X**	-0.7495	-1.272 to -0.2266	Yes
**1648**	Row 13:**10 X** vs. Row 17:**10 X**	-0.5095	-1.032 to 0.01336	No
**1649**	Row 13:**10 X** vs. Row 17:**25 X**	0.1970	-0.3259 to 0.7199	No
**1650**	Row 13:**10 X** vs. 17:**0 X**	-1.011	-1.533 to -0.4876	Yes
**1651**	Row 13:**10 X** vs. 17:**10 X**	-0.8040	-1.327 to -0.2811	Yes
**1652**	Row 13:**10 X** vs. 17:**25 X**	0.2867	-0.1906 to 0.7640	No
**1653**	Row 13:**10 X** vs. Row 19:**0 X**	-1.107	-1.629 to -0.5836	Yes
**1654**	Row 13:**10 X** vs. Row 19:**10 X**	-0.8065	-1.329 to -0.2836	Yes
**1655**	Row 13:**10 X** vs. Row 19:**25 X**	0.3570	-0.1203 to 0.8343	No
**1656**	Row 13:**10 X** vs. Row 20:**0 X**	-1.159	-1.682 to -0.6361	Yes
**1657**	Row 13:**10 X** vs. Row 20:**10 X**	-0.7000	-1.223 to -0.1771	Yes
**1658**	Row 13:**10 X** vs. Row 20:**25 X**	0.4133	-0.06397 to 0.8906	No
**1659**	Row 13:**10 X** vs. Row 21:**0 X**	-0.5160	-1.039 to 0.006859	No
**1660**	Row 13:**10 X** vs. Row 21:**10 X**	-0.0855	-0.6084 to 0.4374	No
**1661**	Row 13:**10 X** vs. Row 21:**25 X**	0.3860	-0.09130 to 0.8633	No
**1662**	Row 13:**25 X** vs. Row 14:**0 X**	-0.4087	-0.8356 to 0.01825	No
**1663**	Row 13:**25 X** vs. Row 14:**10 X**	-0.1847	-0.6116 to 0.2422	No
**1664**	Row 13:**25 X** vs. Row 14:**25 X**	-0.1447	-0.5716 to 0.2822	No
**1665**	Row 13:**25 X** vs. 14:**0 X**	-1.003	-1.430 to -0.5758	Yes

	2way ANOVA<br>Multiple comparisons	A<br>Data Set-A<br>Y	B<br>Data Set-B<br>Y	C<br>Data Set-C<br>Y
**1666**	Row 13:**25 X** vs. 14:**10 X**	-0.3993	-0.8766 to 0.07797	No
**1667**	Row 13:**25 X** vs. 14:**25 X**	-0.03533	-0.5126 to 0.4420	No
**1668**	Row 13:**25 X** vs. Row 16:**0 X**	-1.115	-1.593 to -0.6380	Yes
**1669**	Row 13:**25 X** vs. Row 16:**10 X**	-0.4833	-0.9606 to -0.006031	Yes
**1670**	Row 13:**25 X** vs. Row 16:**25 X**	0.3717	-0.1056 to 0.8490	No
**1671**	Row 13:**25 X** vs. Row 17:**0 X**	-0.7408	-1.218 to -0.2635	Yes
**1672**	Row 13:**25 X** vs. Row 17:**10 X**	-0.5008	-0.9781 to -0.02353	Yes
**1673**	Row 13:**25 X** vs. Row 17:**25 X**	0.2057	-0.2716 to 0.6830	No
**1674**	Row 13:**25 X** vs. 17:**0 X**	-1.002	-1.479 to -0.5245	Yes
**1675**	Row 13:**25 X** vs. 17:**10 X**	-0.7953	-1.273 to -0.3180	Yes
**1676**	Row 13:**25 X** vs. 17:**25 X**	0.2953	-0.1316 to 0.7222	No
**1677**	Row 13:**25 X** vs. Row 19:**0 X**	-1.098	-1.575 to -0.6205	Yes
**1678**	Row 13:**25 X** vs. Row 19:**10 X**	-0.7978	-1.275 to -0.3205	Yes
**1679**	Row 13:**25 X** vs. Row 19:**25 X**	0.3657	-0.06125 to 0.7926	No
**1680**	Row 13:**25 X** vs. Row 20:**0 X**	-1.150	-1.628 to -0.6730	Yes
**1681**	Row 13:**25 X** vs. Row 20:**10 X**	-0.6913	-1.169 to -0.2140	Yes
**1682**	Row 13:**25 X** vs. Row 20:**25 X**	0.4220	-0.004912 to 0.8489	No
**1683**	Row 13:**25 X** vs. Row 21:**0 X**	-0.5073	-0.9846 to -0.03003	Yes
**1684**	Row 13:**25 X** vs. Row 21:**10 X**	-0.07683	-0.5541 to 0.4005	No
**1685**	Row 13:**25 X** vs. Row 21:**25 X**	0.3947	-0.03225 to 0.8216	No
**1686**	Row 14:**0 X** vs. Row 14:**10 X**	0.2240	-0.2029 to 0.6509	No
**1687**	Row 14:**0 X** vs. Row 14:**25 X**	0.2640	-0.1629 to 0.6909	No
**1688**	Row 14:**0 X** vs. 14:**0 X**	-0.5940	-1.021 to -0.1671	Yes
**1689**	Row 14:**0 X** vs. 14:**10 X**	0.009333	-0.4680 to 0.4866	No
**1690**	Row 14:**0 X** vs. 14:**25 X**	0.3733	-0.1040 to 0.8506	No
**1691**	Row 14:**0 X** vs. Row 16:**0 X**	-0.7067	-1.184 to -0.2294	Yes
**1692**	Row 14:**0 X** vs. Row 16:**10 X**	-0.07467	-0.5520 to 0.4026	No
**1693**	Row 14:**0 X** vs. Row 16:**25 X**	0.7803	0.3030 to 1.258	Yes
**1694**	Row 14:**0 X** vs. Row 17:**0 X**	-0.3322	-0.8095 to 0.1451	No
**1695**	Row 14:**0 X** vs. Row 17:**10 X**	-0.09217	-0.5695 to 0.3851	No
**1696**	Row 14:**0 X** vs. Row 17:**25 X**	0.6143	0.1370 to 1.092	Yes
**1697**	Row 14:**0 X** vs. 17:**0 X**	-0.5932	-1.070 to -0.1159	Yes
**1698**	Row 14:**0 X** vs. 17:**10 X**	-0.3867	-0.8640 to 0.09064	No
**1699**	Row 14:**0 X** vs. 17:**25 X**	0.7040	0.2771 to 1.131	Yes
**1700**	Row 14:**0 X** vs. Row 19:**0 X**	-0.6892	-1.166 to -0.2119	Yes
**1701**	Row 14:**0 X** vs. Row 19:**10 X**	-0.3892	-0.8665 to 0.08814	No
**1702**	Row 14:**0 X** vs. Row 19:**25 X**	0.7743	0.3474 to 1.201	Yes
**1703**	Row 14:**0 X** vs. Row 20:**0 X**	-0.7417	-1.219 to -0.2644	Yes
**1704**	Row 14:**0 X** vs. Row 20:**10 X**	-0.2827	-0.7600 to 0.1946	No
**1705**	Row 14:**0 X** vs. Row 20:**25 X**	0.8307	0.4038 to 1.258	Yes
**1706**	Row 14:**0 X** vs. Row 21:**0 X**	-0.09867	-0.5760 to 0.3786	No
**1707**	Row 14:**0 X** vs. Row 21:**10 X**	0.3318	-0.1455 to 0.8091	No
**1708**	Row 14:**0 X** vs. Row 21:**25 X**	0.8033	0.3764 to 1.230	Yes
**1709**	Row 14:**10 X** vs. Row 14:**25 X**	0.04000	-0.3869 to 0.4669	No
**1710**	Row 14:**10 X** vs. 14:**0 X**	-0.8180	-1.245 to -0.3911	Yes

	2way ANOVA<br>Multiple comparisons	A<br>Data Set-A<br>Y	B<br>Data Set-B<br>Y	C<br>Data Set-C<br>Y
**1711**	Row 14:**10 X** vs. 14:**10 X**	-0.2147	-0.6920 to 0.2626	No
**1712**	Row 14:**10 X** vs. 14:**25 X**	0.1493	-0.3280 to 0.6266	No
**1713**	Row 14:**10 X** vs. Row 16:**0 X**	-0.9307	-1.408 to -0.4534	Yes
**1714**	Row 14:**10 X** vs. Row 16:**10 X**	-0.2987	-0.7760 to 0.1786	No
**1715**	Row 14:**10 X** vs. Row 16:**25 X**	0.5563	0.07903 to 1.034	Yes
**1716**	Row 14:**10 X** vs. Row 17:**0 X**	-0.5562	-1.033 to -0.07886	Yes
**1717**	Row 14:**10 X** vs. Row 17:**10 X**	-0.3162	-0.7935 to 0.1611	No
**1718**	Row 14:**10 X** vs. Row 17:**25 X**	0.3903	-0.08697 to 0.8676	No
**1719**	Row 14:**10 X** vs. 17:**0 X**	-0.8172	-1.294 to -0.3399	Yes
**1720**	Row 14:**10 X** vs. 17:**10 X**	-0.6107	-1.088 to -0.1334	Yes
**1721**	Row 14:**10 X** vs. 17:**25 X**	0.4800	0.05309 to 0.9069	Yes
**1722**	Row 14:**10 X** vs. Row 19:**0 X**	-0.9132	-1.390 to -0.4359	Yes
**1723**	Row 14:**10 X** vs. Row 19:**10 X**	-0.6132	-1.090 to -0.1359	Yes
**1724**	Row 14:**10 X** vs. Row 19:**25 X**	0.5503	0.1234 to 0.9772	Yes
**1725**	Row 14:**10 X** vs. Row 20:**0 X**	-0.9657	-1.443 to -0.4884	Yes
**1726**	Row 14:**10 X** vs. Row 20:**10 X**	-0.5067	-0.9840 to -0.02936	Yes
**1727**	Row 14:**10 X** vs. Row 20:**25 X**	0.6067	0.1798 to 1.034	Yes
**1728**	Row 14:**10 X** vs. Row 21:**0 X**	-0.3227	-0.8000 to 0.1546	No
**1729**	Row 14:**10 X** vs. Row 21:**10 X**	0.1078	-0.3695 to 0.5851	No
**1730**	Row 14:**10 X** vs. Row 21:**25 X**	0.5793	0.1524 to 1.006	Yes
**1731**	Row 14:**25 X** vs. 14:**0 X**	-0.8580	-1.285 to -0.4311	Yes
**1732**	Row 14:**25 X** vs. 14:**10 X**	-0.2547	-0.7320 to 0.2226	No
**1733**	Row 14:**25 X** vs. 14:**25 X**	0.1093	-0.3680 to 0.5866	No
**1734**	Row 14:**25 X** vs. Row 16:**0 X**	-0.9707	-1.448 to -0.4934	Yes
**1735**	Row 14:**25 X** vs. Row 16:**10 X**	-0.3387	-0.8160 to 0.1386	No
**1736**	Row 14:**25 X** vs. Row 16:**25 X**	0.5163	0.03903 to 0.9936	Yes
**1737**	Row 14:**25 X** vs. Row 17:**0 X**	-0.5962	-1.073 to -0.1189	Yes
**1738**	Row 14:**25 X** vs. Row 17:**10 X**	-0.3562	-0.8335 to 0.1211	No
**1739**	Row 14:**25 X** vs. Row 17:**25 X**	0.3503	-0.1270 to 0.8276	No
**1740**	Row 14:**25 X** vs. 17:**0 X**	-0.8572	-1.334 to -0.3799	Yes
**1741**	Row 14:**25 X** vs. 17:**10 X**	-0.6507	-1.128 to -0.1734	Yes
**1742**	Row 14:**25 X** vs. 17:**25 X**	0.4400	0.01309 to 0.8669	Yes
**1743**	Row 14:**25 X** vs. Row 19:**0 X**	-0.9532	-1.430 to -0.4759	Yes
**1744**	Row 14:**25 X** vs. Row 19:**10 X**	-0.6532	-1.130 to -0.1759	Yes
**1745**	Row 14:**25 X** vs. Row 19:**25 X**	0.5103	0.08342 to 0.9372	Yes
**1746**	Row 14:**25 X** vs. Row 20:**0 X**	-1.006	-1.483 to -0.5284	Yes
**1747**	Row 14:**25 X** vs. Row 20:**10 X**	-0.5467	-1.024 to -0.06936	Yes
**1748**	Row 14:**25 X** vs. Row 20:**25 X**	0.5667	0.1398 to 0.9936	Yes
**1749**	Row 14:**25 X** vs. Row 21:**0 X**	-0.3627	-0.8400 to 0.1146	No
**1750**	Row 14:**25 X** vs. Row 21:**10 X**	0.06783	-0.4095 to 0.5451	No
**1751**	Row 14:**25 X** vs. Row 21:**25 X**	0.5393	0.1124 to 0.9662	Yes
**1752**	14:**0 X** vs. 14:**10 X**	0.6033	0.1260 to 1.081	Yes
**1753**	14:**0 X** vs. 14:**25 X**	0.9673	0.4900 to 1.445	Yes
**1754**	14:**0 X** vs. Row 16:**0 X**	-0.1127	-0.5900 to 0.3646	No
**1755**	14:**0 X** vs. Row 16:**10 X**	0.5193	0.04203 to 0.9966	Yes

	2way ANOVA<br>Multiple comparisons	A<br>Data Set-A<br>Y	B<br>Data Set-B<br>Y	C<br>Data Set-C<br>Y
**1756**	14:**0 X** vs. Row 16:**25 X**	1.374	0.8970 to 1.852	Yes
**1757**	14:**0 X** vs. Row 17:**0 X**	0.2618	-0.2155 to 0.7391	No
**1758**	14:**0 X** vs. Row 17:**10 X**	0.5018	0.02453 to 0.9791	Yes
**1759**	14:**0 X** vs. Row 17:**25 X**	1.208	0.7310 to 1.686	Yes
**1760**	14:**0 X** vs. 17:**0 X**	0.0008335	-0.4765 to 0.4781	No
**1761**	14:**0 X** vs. 17:**10 X**	0.2073	-0.2700 to 0.6846	No
**1762**	14:**0 X** vs. 17:**25 X**	1.298	0.8711 to 1.725	Yes
**1763**	14:**0 X** vs. Row 19:**0 X**	-0.09517	-0.5725 to 0.3821	No
**1764**	14:**0 X** vs. Row 19:**10 X**	0.2048	-0.2725 to 0.6821	No
**1765**	14:**0 X** vs. Row 19:**25 X**	1.368	0.9414 to 1.795	Yes
**1766**	14:**0 X** vs. Row 20:**0 X**	-0.1477	-0.6250 to 0.3296	No
**1767**	14:**0 X** vs. Row 20:**10 X**	0.3113	-0.1660 to 0.7886	No
**1768**	14:**0 X** vs. Row 20:**25 X**	1.425	0.9978 to 1.852	Yes
**1769**	14:**0 X** vs. Row 21:**0 X**	0.4953	0.01803 to 0.9726	Yes
**1770**	14:**0 X** vs. Row 21:**10 X**	0.9258	0.4485 to 1.403	Yes
**1771**	14:**0 X** vs. Row 21:**25 X**	1.397	0.9704 to 1.824	Yes
**1772**	14:**10 X** vs. 14:**25 X**	0.3640	-0.1589 to 0.8869	No
**1773**	14:**10 X** vs. Row 16:**0 X**	-0.7160	-1.239 to -0.1931	Yes
**1774**	14:**10 X** vs. Row 16:**10 X**	-0.08400	-0.6069 to 0.4389	No
**1775**	14:**10 X** vs. Row 16:**25 X**	0.7710	0.2481 to 1.294	Yes
**1776**	14:**10 X** vs. Row 17:**0 X**	-0.3415	-0.8644 to 0.1814	No
**1777**	14:**10 X** vs. Row 17:**10 X**	-0.1015	-0.6244 to 0.4214	No
**1778**	14:**10 X** vs. Row 17:**25 X**	0.6050	0.08214 to 1.128	Yes
**1779**	14:**10 X** vs. 17:**0 X**	-0.6025	-1.125 to -0.07964	Yes
**1780**	14:**10 X** vs. 17:**10 X**	-0.3960	-0.9189 to 0.1269	No
**1781**	14:**10 X** vs. 17:**25 X**	0.6947	0.2174 to 1.172	Yes
**1782**	14:**10 X** vs. Row 19:**0 X**	-0.6985	-1.221 to -0.1756	Yes
**1783**	14:**10 X** vs. Row 19:**10 X**	-0.3985	-0.9214 to 0.1244	No
**1784**	14:**10 X** vs. Row 19:**25 X**	0.7650	0.2877 to 1.242	Yes
**1785**	14:**10 X** vs. Row 20:**0 X**	-0.7510	-1.274 to -0.2281	Yes
**1786**	14:**10 X** vs. Row 20:**10 X**	-0.2920	-0.8149 to 0.2309	No
**1787**	14:**10 X** vs. Row 20:**25 X**	0.8213	0.3440 to 1.299	Yes
**1788**	14:**10 X** vs. Row 21:**0 X**	-0.1080	-0.6309 to 0.4149	No
**1789**	14:**10 X** vs. Row 21:**10 X**	0.3225	-0.2004 to 0.8454	No
**1790**	14:**10 X** vs. Row 21:**25 X**	0.7940	0.3167 to 1.271	Yes
**1791**	14:**25 X** vs. Row 16:**0 X**	-1.080	-1.603 to -0.5571	Yes
**1792**	14:**25 X** vs. Row 16:**10 X**	-0.4480	-0.9709 to 0.07486	No
**1793**	14:**25 X** vs. Row 16:**25 X**	0.4070	-0.1159 to 0.9299	No
**1794**	14:**25 X** vs. Row 17:**0 X**	-0.7055	-1.228 to -0.1826	Yes
**1795**	14:**25 X** vs. Row 17:**10 X**	-0.4655	-0.9884 to 0.05736	No
**1796**	14:**25 X** vs. Row 17:**25 X**	0.2410	-0.2819 to 0.7639	No
**1797**	14:**25 X** vs. 17:**0 X**	-0.9665	-1.489 to -0.4436	Yes
**1798**	14:**25 X** vs. 17:**10 X**	-0.7600	-1.283 to -0.2371	Yes
**1799**	14:**25 X** vs. 17:**25 X**	0.3307	-0.1466 to 0.8080	No
**1800**	14:**25 X** vs. Row 19:**0 X**	-1.063	-1.585 to -0.5396	Yes

	2way ANOVA<br>Multiple comparisons	A<br>Data Set-A<br>Y	B<br>Data Set-B<br>Y	C<br>Data Set-C<br>Y
1801	14:**25 X** vs. Row 19:**10 X**	-0.7625	-1.285 to -0.2396	Yes
1802	14:**25 X** vs. Row 19:**25 X**	0.4010	-0.07630 to 0.8783	No
1803	14:**25 X** vs. Row 20:**0 X**	-1.115	-1.638 to -0.5921	Yes
1804	14:**25 X** vs. Row 20:**10 X**	-0.6560	-1.179 to -0.1331	Yes
1805	14:**25 X** vs. Row 20:**25 X**	0.4573	-0.01997 to 0.9346	No
1806	14:**25 X** vs. Row 21:**0 X**	-0.4720	-0.9949 to 0.05086	No
1807	14:**25 X** vs. Row 21:**10 X**	-0.04150	-0.5644 to 0.4814	No
1808	14:**25 X** vs. Row 21:**25 X**	0.4300	-0.04730 to 0.9073	No
1809	Row 16:**0 X** vs. Row 16:**10 X**	0.6320	0.1091 to 1.155	Yes
1810	Row 16:**0 X** vs. Row 16:**25 X**	1.487	0.9641 to 2.010	Yes
1811	Row 16:**0 X** vs. Row 17:**0 X**	0.3745	-0.1484 to 0.8974	No
1812	Row 16:**0 X** vs. Row 17:**10 X**	0.6145	0.09164 to 1.137	Yes
1813	Row 16:**0 X** vs. Row 17:**25 X**	1.321	0.7981 to 1.844	Yes
1814	Row 16:**0 X** vs. 17:**0 X**	0.1135	-0.4094 to 0.6364	No
1815	Row 16:**0 X** vs. 17:**10 X**	0.3200	-0.2029 to 0.8429	No
1816	Row 16:**0 X** vs. 17:**25 X**	1.411	0.9334 to 1.888	Yes
1817	Row 16:**0 X** vs. Row 19:**0 X**	0.01750	-0.5054 to 0.5404	No
1818	Row 16:**0 X** vs. Row 19:**10 X**	0.3175	-0.2054 to 0.8404	No
1819	Row 16:**0 X** vs. Row 19:**25 X**	1.481	1.004 to 1.958	Yes
1820	Row 16:**0 X** vs. Row 20:**0 X**	-0.03500	-0.5579 to 0.4879	No
1821	Row 16:**0 X** vs. Row 20:**10 X**	0.4240	-0.09886 to 0.9469	No
1822	Row 16:**0 X** vs. Row 20:**25 X**	1.537	1.060 to 2.015	Yes
1823	Row 16:**0 X** vs. Row 21:**0 X**	0.6080	0.08514 to 1.131	Yes
1824	Row 16:**0 X** vs. Row 21:**10 X**	1.039	0.5156 to 1.561	Yes
1825	Row 16:**0 X** vs. Row 21:**25 X**	1.510	1.033 to 1.987	Yes
1826	Row 16:**10 X** vs. Row 16:**25 X**	0.8550	0.3321 to 1.378	Yes
1827	Row 16:**10 X** vs. Row 17:**0 X**	-0.2575	-0.7804 to 0.2654	No
1828	Row 16:**10 X** vs. Row 17:**10 X**	-0.01750	-0.5404 to 0.5054	No
1829	Row 16:**10 X** vs. Row 17:**25 X**	0.6890	0.1661 to 1.212	Yes
1830	Row 16:**10 X** vs. 17:**0 X**	-0.5185	-1.041 to 0.004359	No
1831	Row 16:**10 X** vs. 17:**10 X**	-0.3120	-0.8349 to 0.2109	No
1832	Row 16:**10 X** vs. 17:**25 X**	0.7787	0.3014 to 1.256	Yes
1833	Row 16:**10 X** vs. Row 19:**0 X**	-0.6145	-1.137 to -0.09164	Yes
1834	Row 16:**10 X** vs. Row 19:**10 X**	-0.3145	-0.8374 to 0.2084	No
1835	Row 16:**10 X** vs. Row 19:**25 X**	0.8490	0.3717 to 1.326	Yes
1836	Row 16:**10 X** vs. Row 20:**0 X**	-0.6670	-1.190 to -0.1441	Yes
1837	Row 16:**10 X** vs. Row 20:**10 X**	-0.2080	-0.7309 to 0.3149	No
1838	Row 16:**10 X** vs. Row 20:**25 X**	0.9053	0.4280 to 1.383	Yes
1839	Row 16:**10 X** vs. Row 21:**0 X**	-0.02400	-0.5469 to 0.4989	No
1840	Row 16:**10 X** vs. Row 21:**10 X**	0.4065	-0.1164 to 0.9294	No
1841	Row 16:**10 X** vs. Row 21:**25 X**	0.8780	0.4007 to 1.355	Yes
1842	Row 16:**25 X** vs. Row 17:**0 X**	-1.113	-1.635 to -0.5896	Yes
1843	Row 16:**25 X** vs. Row 17:**10 X**	-0.8725	-1.395 to -0.3496	Yes
1844	Row 16:**25 X** vs. Row 17:**25 X**	-0.1660	-0.6889 to 0.3569	No
1845	Row 16:**25 X** vs. 17:**0 X**	-1.374	-1.896 to -0.8506	Yes

	**2way ANOVA** Multiple comparisons	**A** Data Set-A **Y**	**B** Data Set-B **Y**	**C** Data Set-C **Y**
**1846**	Row 16:**25 X** vs. 17:**10 X**	-1.167	-1.690 to -0.6441	Yes
**1847**	Row 16:**25 X** vs. 17:**25 X**	-0.07633	-0.5536 to 0.4010	No
**1848**	Row 16:**25 X** vs. Row 19:**0 X**	-1.470	-1.992 to -0.9466	Yes
**1849**	Row 16:**25 X** vs. Row 19:**10 X**	-1.170	-1.692 to -0.6466	Yes
**1850**	Row 16:**25 X** vs. Row 19:**25 X**	-0.006000	-0.4833 to 0.4713	No
**1851**	Row 16:**25 X** vs. Row 20:**0 X**	-1.522	-2.045 to -0.9991	Yes
**1852**	Row 16:**25 X** vs. Row 20:**10 X**	-1.063	-1.586 to -0.5401	Yes
**1853**	Row 16:**25 X** vs. Row 20:**25 X**	0.05033	-0.4270 to 0.5276	No
**1854**	Row 16:**25 X** vs. Row 21:**0 X**	-0.8790	-1.402 to -0.3561	Yes
**1855**	Row 16:**25 X** vs. Row 21:**10 X**	-0.4485	-0.9714 to 0.07436	No
**1856**	Row 16:**25 X** vs. Row 21:**25 X**	0.02300	-0.4543 to 0.5003	No
**1857**	Row 17:**0 X** vs. Row 17:**10 X**	0.2400	-0.2829 to 0.7629	No
**1858**	Row 17:**0 X** vs. Row 17:**25 X**	0.9465	0.4236 to 1.469	Yes
**1859**	Row 17:**0 X** vs. 17:**0 X**	-0.2610	-0.7839 to 0.2619	No
**1860**	Row 17:**0 X** vs. 17:**10 X**	-0.05450	-0.5774 to 0.4684	No
**1861**	Row 17:**0 X** vs. 17:**25 X**	1.036	0.5589 to 1.513	Yes
**1862**	Row 17:**0 X** vs. Row 19:**0 X**	-0.3570	-0.8799 to 0.1659	No
**1863**	Row 17:**0 X** vs. Row 19:**10 X**	-0.05700	-0.5799 to 0.4659	No
**1864**	Row 17:**0 X** vs. Row 19:**25 X**	1.107	0.6292 to 1.584	Yes
**1865**	Row 17:**0 X** vs. Row 20:**0 X**	-0.4095	-0.9324 to 0.1134	No
**1866**	Row 17:**0 X** vs. Row 20:**10 X**	0.04950	-0.4734 to 0.5724	No
**1867**	Row 17:**0 X** vs. Row 20:**25 X**	1.163	0.6855 to 1.640	Yes
**1868**	Row 17:**0 X** vs. Row 21:**0 X**	0.2335	-0.2894 to 0.7564	No
**1869**	Row 17:**0 X** vs. Row 21:**10 X**	0.6640	0.1411 to 1.187	Yes
**1870**	Row 17:**0 X** vs. Row 21:**25 X**	1.136	0.6582 to 1.613	Yes
**1871**	Row 17:**10 X** vs. Row 17:**25 X**	0.7065	0.1836 to 1.229	Yes
**1872**	Row 17:**10 X** vs. 17:**0 X**	-0.5010	-1.024 to 0.02186	No
**1873**	Row 17:**10 X** vs. 17:**10 X**	-0.2945	-0.8174 to 0.2284	No
**1874**	Row 17:**10 X** vs. 17:**25 X**	0.7962	0.3189 to 1.273	Yes
**1875**	Row 17:**10 X** vs. Row 19:**0 X**	-0.5970	-1.120 to -0.07414	Yes
**1876**	Row 17:**10 X** vs. Row 19:**10 X**	-0.2970	-0.8199 to 0.2259	No
**1877**	Row 17:**10 X** vs. Row 19:**25 X**	0.8665	0.3892 to 1.344	Yes
**1878**	Row 17:**10 X** vs. Row 20:**0 X**	-0.6495	-1.172 to -0.1266	Yes
**1879**	Row 17:**10 X** vs. Row 20:**10 X**	-0.1905	-0.7134 to 0.3324	No
**1880**	Row 17:**10 X** vs. Row 20:**25 X**	0.9228	0.4455 to 1.400	Yes
**1881**	Row 17:**10 X** vs. Row 21:**0 X**	-0.006500	-0.5294 to 0.5164	No
**1882**	Row 17:**10 X** vs. Row 21:**10 X**	0.4240	-0.09886 to 0.9469	No
**1883**	Row 17:**10 X** vs. Row 21:**25 X**	0.8955	0.4182 to 1.373	Yes
**1884**	Row 17:**25 X** vs. 17:**0 X**	-1.208	-1.730 to -0.6846	Yes
**1885**	Row 17:**25 X** vs. 17:**10 X**	-1.001	-1.524 to -0.4781	Yes
**1886**	Row 17:**25 X** vs. 17:**25 X**	0.08967	-0.3876 to 0.5670	No
**1887**	Row 17:**25 X** vs. Row 19:**0 X**	-1.304	-1.826 to -0.7806	Yes
**1888**	Row 17:**25 X** vs. Row 19:**10 X**	-1.004	-1.526 to -0.4806	Yes
**1889**	Row 17:**25 X** vs. Row 19:**25 X**	0.1600	-0.3173 to 0.6373	No
**1890**	Row 17:**25 X** vs. Row 20:**0 X**	-1.356	-1.879 to -0.8331	Yes

	2way ANOVA<br>Multiple comparisons	A<br>Data Set-A<br>Y	B<br>Data Set-B<br>Y	C<br>Data Set-C<br>Y
1891	Row 17:**25 X** vs. Row 20:**10 X**	-0.8970	-1.420 to -0.3741	Yes
1892	Row 17:**25 X** vs. Row 20:**25 X**	0.2163	-0.2610 to 0.6936	No
1893	Row 17:**25 X** vs. Row 21:**0 X**	-0.7130	-1.236 to -0.1901	Yes
1894	Row 17:**25 X** vs. Row 21:**10 X**	-0.2825	-0.8054 to 0.2404	No
1895	Row 17:**25 X** vs. Row 21:**25 X**	0.1890	-0.2883 to 0.6663	No
1896	17:**0 X** vs. 17:**10 X**	0.2065	-0.3164 to 0.7294	No
1897	17:**0 X** vs. 17:**25 X**	1.297	0.8199 to 1.774	Yes
1898	17:**0 X** vs. Row 19:**0 X**	-0.09600	-0.6189 to 0.4269	No
1899	17:**0 X** vs. Row 19:**10 X**	0.2040	-0.3189 to 0.7269	No
1900	17:**0 X** vs. Row 19:**25 X**	1.368	0.8902 to 1.845	Yes
1901	17:**0 X** vs. Row 20:**0 X**	-0.1485	-0.6714 to 0.3744	No
1902	17:**0 X** vs. Row 20:**10 X**	0.3105	-0.2124 to 0.8334	No
1903	17:**0 X** vs. Row 20:**25 X**	1.424	0.9465 to 1.901	Yes
1904	17:**0 X** vs. Row 21:**0 X**	0.4945	-0.02836 to 1.017	No
1905	17:**0 X** vs. Row 21:**10 X**	0.9250	0.4021 to 1.448	Yes
1906	17:**0 X** vs. Row 21:**25 X**	1.397	0.9192 to 1.874	Yes
1907	17:**10 X** vs. 17:**25 X**	1.091	0.6134 to 1.568	Yes
1908	17:**10 X** vs. Row 19:**0 X**	-0.3025	-0.8254 to 0.2204	No
1909	17:**10 X** vs. Row 19:**10 X**	-0.002500	-0.5254 to 0.5204	No
1910	17:**10 X** vs. Row 19:**25 X**	1.161	0.6837 to 1.638	Yes
1911	17:**10 X** vs. Row 20:**0 X**	-0.3550	-0.8779 to 0.1679	No
1912	17:**10 X** vs. Row 20:**10 X**	0.1040	-0.4189 to 0.6269	No
1913	17:**10 X** vs. Row 20:**25 X**	1.217	0.7400 to 1.695	Yes
1914	17:**10 X** vs. Row 21:**0 X**	0.2880	-0.2349 to 0.8109	No
1915	17:**10 X** vs. Row 21:**10 X**	0.7185	0.1956 to 1.241	Yes
1916	17:**10 X** vs. Row 21:**25 X**	1.190	0.7127 to 1.667	Yes
1917	17:**25 X** vs. Row 19:**0 X**	-1.393	-1.870 to -0.9159	Yes
1918	17:**25 X** vs. Row 19:**10 X**	-1.093	-1.570 to -0.6159	Yes
1919	17:**25 X** vs. Row 19:**25 X**	0.07033	-0.3566 to 0.4972	No
1920	17:**25 X** vs. Row 20:**0 X**	-1.446	-1.923 to -0.9684	Yes
1921	17:**25 X** vs. Row 20:**10 X**	-0.9867	-1.464 to -0.5094	Yes
1922	17:**25 X** vs. Row 20:**25 X**	0.1267	-0.3002 to 0.5536	No
1923	17:**25 X** vs. Row 21:**0 X**	-0.8027	-1.280 to -0.3254	Yes
1924	17:**25 X** vs. Row 21:**10 X**	-0.3722	-0.8495 to 0.1051	No
1925	17:**25 X** vs. Row 21:**25 X**	0.09933	-0.3276 to 0.5262	No
1926	Row 19:**0 X** vs. Row 19:**10 X**	0.3000	-0.2229 to 0.8229	No
1927	Row 19:**0 X** vs. Row 19:**25 X**	1.464	0.9862 to 1.941	Yes
1928	Row 19:**0 X** vs. Row 20:**0 X**	-0.05250	-0.5754 to 0.4704	No
1929	Row 19:**0 X** vs. Row 20:**10 X**	0.4065	-0.1164 to 0.9294	No
1930	Row 19:**0 X** vs. Row 20:**25 X**	1.520	1.043 to 1.997	Yes
1931	Row 19:**0 X** vs. Row 21:**0 X**	0.5905	0.06764 to 1.113	Yes
1932	Row 19:**0 X** vs. Row 21:**10 X**	1.021	0.4981 to 1.544	Yes
1933	Row 19:**0 X** vs. Row 21:**25 X**	1.493	1.015 to 1.970	Yes
1934	Row 19:**10 X** vs. Row 19:**25 X**	1.164	0.6862 to 1.641	Yes
1935	Row 19:**10 X** vs. Row 20:**0 X**	-0.3525	-0.8754 to 0.1704	No

	**2way ANOVA** Multiple comparisons	**A** Data Set-A **Y**	**B** Data Set-B **Y**	**C** Data Set-C **Y**
**1936**	Row 19:**10 X** vs. Row 20:**10 X**	0.1065	-0.4164 to 0.6294	No
**1937**	Row 19:**10 X** vs. Row 20:**25 X**	1.220	0.7425 to 1.697	Yes
**1938**	Row 19:**10 X** vs. Row 21:**0 X**	0.2905	-0.2324 to 0.8134	No
**1939**	Row 19:**10 X** vs. Row 21:**10 X**	0.7210	0.1981 to 1.244	Yes
**1940**	Row 19:**10 X** vs. Row 21:**25 X**	1.193	0.7152 to 1.670	Yes
**1941**	Row 19:**25 X** vs. Row 20:**0 X**	-1.516	-1.993 to -1.039	Yes
**1942**	Row 19:**25 X** vs. Row 20:**10 X**	-1.057	-1.534 to -0.5797	Yes
**1943**	Row 19:**25 X** vs. Row 20:**25 X**	0.05633	-0.3706 to 0.4832	No
**1944**	Row 19:**25 X** vs. Row 21:**0 X**	-0.8730	-1.350 to -0.3957	Yes
**1945**	Row 19:**25 X** vs. Row 21:**10 X**	-0.4425	-0.9198 to 0.03480	No
**1946**	Row 19:**25 X** vs. Row 21:**25 X**	0.02900	-0.3979 to 0.4559	No
**1947**	Row 20:**0 X** vs. Row 20:**10 X**	0.4590	-0.06386 to 0.9819	No
**1948**	Row 20:**0 X** vs. Row 20:**25 X**	1.572	1.095 to 2.050	Yes
**1949**	Row 20:**0 X** vs. Row 21:**0 X**	0.6430	0.1201 to 1.166	Yes
**1950**	Row 20:**0 X** vs. Row 21:**10 X**	1.074	0.5506 to 1.596	Yes
**1951**	Row 20:**0 X** vs. Row 21:**25 X**	1.545	1.068 to 2.022	Yes
**1952**	Row 20:**10 X** vs. Row 20:**25 X**	1.113	0.6360 to 1.591	Yes
**1953**	Row 20:**10 X** vs. Row 21:**0 X**	0.1840	-0.3389 to 0.7069	No
**1954**	Row 20:**10 X** vs. Row 21:**10 X**	0.6145	0.09164 to 1.137	Yes
**1955**	Row 20:**10 X** vs. Row 21:**25 X**	1.086	0.6087 to 1.563	Yes
**1956**	Row 20:**25 X** vs. Row 21:**0 X**	-0.9293	-1.407 to -0.4520	Yes
**1957**	Row 20:**25 X** vs. Row 21:**10 X**	-0.4988	-0.9761 to -0.02153	Yes
**1958**	Row 20:**25 X** vs. Row 21:**25 X**	-0.02733	-0.4542 to 0.3996	No
**1959**	Row 21:**0 X** vs. Row 21:**10 X**	0.4305	-0.09236 to 0.9534	No
**1960**	Row 21:**0 X** vs. Row 21:**25 X**	0.9020	0.4247 to 1.379	Yes
**1961**	Row 21:**10 X** vs. Row 21:**25 X**	0.4715	-0.005803 to 0.9488	No
**1962**				
**1963**				
**1964**	Test details	Mean 1	Mean 2	Mean Diff.
**1965**				
**1966**	Row 1:**0 X** vs. Row 1:**10 X**	0.0500	0.0500	0.0
**1967**	Row 1:**0 X** vs. Row 1:**25 X**	0.0500	0.0500	0.0
**1968**	Row 1:**0 X** vs. Row 2:**0 X**	0.0500	0.0130	0.0370
**1969**	Row 1:**0 X** vs. Row 2:**10 X**	0.0500	0.01667	0.03333
**1970**	Row 1:**0 X** vs. Row 2:**25 X**	0.0500	0.01467	0.03533
**1971**	Row 1:**0 X** vs. Row 3:**0 X**	0.0500	0.02867	0.02133
**1972**	Row 1:**0 X** vs. Row 3:**10 X**	0.0500	0.02867	0.02133
**1973**	Row 1:**0 X** vs. Row 3:**25 X**	0.0500	0.02633	0.02367
**1974**	Row 1:**0 X** vs. Row 4:**0 X**	0.0500	0.0640	-0.0140
**1975**	Row 1:**0 X** vs. Row 4:**10 X**	0.0500	0.05767	-0.007667
**1976**	Row 1:**0 X** vs. Row 4:**25 X**	0.0500	0.07033	-0.02033
**1977**	Row 1:**0 X** vs. Row 5:**0 X**	0.0500	0.3287	-0.2787
**1978**	Row 1:**0 X** vs. Row 5:**10 X**	0.0500	0.2960	-0.2460
**1979**	Row 1:**0 X** vs. Row 5:**25 X**	0.0500	0.2740	-0.2240
**1980**	Row 1:**0 X** vs. Row 6:**0 X**	0.0500	0.8727	-0.8227

Cell Viability

(2way ANOVA) Multiple Comparisons.

**2way ANOVA**<br>Multiple comparisons	**A**<br>Data Set-A<br>**Y**	**B**<br>Data Set-B<br>**Y**	**C**<br>Data Set-C<br>**Y**	**D**<br>Data Set-D<br>**Y**
Compare cell means regardless of rows and columns				
Number of families	1			
Number of comparisons per family	36			
Alpha	0.05			
Tukey's multiple comparisons test	Mean Diff.	95% CI of diff.	Significant?	Summary
Mid log:**0 X** vs. Mid log:**10 X**	0.1581	-0.3978 to 0.7139	No	ns
Mid log:**0 X** vs. Mid log:**25 X**	0.3774	-0.1784 to 0.9332	No	ns
Mid log:**0 X** vs. Late log:**0 X**	0.09402	-0.4618 to 0.6498	No	ns
Mid log:**0 X** vs. Late log:**10 X**	0.2779	-0.2779 to 0.8337	No	ns
Mid log:**0 X** vs. Late log:**25 X**	0.4580	-0.09782 to 1.014	No	ns
Mid log:**0 X** vs. Stationary:**0 X** Mid	0.3859	-0.1699 to 0.9417	No	ns
log:**0 X** vs. Stationary:**10 X** Mid	0.6145	0.05870 to 1.170	Yes	*
log:**0 X** vs. Stationary:**25 X** Mid	1.033	0.4771 to 1.589	Yes	***
log:**10 X** vs. Mid log:**25 X**	0.2193	-0.3365 to 0.7751	No	ns
Mid log:**10 X** vs. Late log:**0 X**	-0.06405	-0.6199 to 0.4918	No	ns
Mid log:**10 X** vs. Late log:**10 X**	0.1198	-0.4360 to 0.6756	No	ns
Mid log:**10 X** vs. Late log:**25 X** Mid	0.2999	-0.2559 to 0.8558	No	ns
log:**10 X** vs. Stationary:**0 X** Mid	0.2278	-0.3280 to 0.7837	No	ns
log:**10 X** vs. Stationary:**10 X** Mid	0.4565	-0.09937 to 1.012	No	ns
log:**10 X** vs. Stationary:**25 X** Mid	0.8749	0.3191 to 1.431	Yes	***
log:**25 X** vs. Late log:**0 X**	-0.2834	-0.8392 to 0.2724	No	ns
Mid log:**25 X** vs. Late log:**10 X**	-0.09952	-0.6553 to 0.4563	No	ns
Mid log:**25 X** vs. Late log:**25 X** Mid	0.08061	-0.4752 to 0.6364	No	ns
log:**25 X** vs. Stationary:**0 X** Mid	0.008519	-0.5473 to 0.5643	No	ns
log:**25 X** vs. Stationary:**10 X** Mid	0.2371	-0.3187 to 0.7930	No	ns
log:**25 X** vs. Stationary:**25 X** Late	0.6556	0.09975 to 1.211	Yes	*
log:**0 X** vs. Late log:**10 X** Late log:**0**	0.1839	-0.3720 to 0.7397	No	ns
**X** vs. Late log:**25 X** Late log:**0 X** vs.	0.3640	-0.1918 to 0.9198	No	ns
Stationary:**0 X** Late log:**0 X** vs.	0.2919	-0.2639 to 0.8477	No	ns
Stationary:**10 X** Late log:**0 X** vs.	0.5205	-0.03531 to 1.076	No	ns
Stationary:**25 X** Late log:**10 X** vs.	0.9389	0.3831 to 1.495	Yes	***
Late log:**25 X** Late log:**10 X** vs.	0.1801	-0.3757 to 0.7360	No	ns
Stationary:**0 X** Late log:**10 X** vs.	0.1080	-0.4478 to 0.6639	No	ns
Stationary:**10 X** Late log:**10 X** vs.	0.3367	-0.2192 to 0.8925	No	ns
Stationary:**25 X** Late log:**25 X** vs.	0.7551	0.1993 to 1.311	Yes	**
Stationary:**0 X** Late log:**25 X** vs.	-0.07209	-0.6279 to 0.4837	No	ns
Stationary:**10 X** Late log:**25 X** vs.	0.1565	-0.3993 to 0.7123	No	ns
Stationary:**25 X** Stationary:**0 X** vs.	0.5750	0.01914 to 1.131	Yes	*
Stationary:**10 X** Stationary:**0 X** vs.	0.2286	-0.3272 to 0.7844	No	ns
Stationary:**25 X** Stationary:**10 X** vs.	0.6470	0.09123 to 1.203	Yes	*
Stationary:**25 X**	0.4184	-0.1374 to 0.9743	No	ns
Test details	Mean 1	Mean 2	Mean Diff.	SE of diff.
Mid log:**0 X** vs. Mid log:**10 X**	3.488	3.330	0.1581	0.1586
Mid log:**0 X** vs. Mid log:**25 X**	3.488	3.111	0.3774	0.1586

	2way ANOVA<br>Multiple comparisons	A<br>Data Set-A<br>Y	B<br>Data Set-B<br>Y	C<br>Data Set-C<br>Y	D<br>Data Set-D<br>Y
**51**	Mid log:**0 X** vs. Late log:**0 X**	3.488	3.394	0.09402	0.1586
**52**	Mid log:**0 X** vs. Late log:**10 X**	3.488	3.211	0.2779	0.1586
**53**	Mid log:**0 X** vs. Late log:**25 X**	3.488	3.030	0.4580	0.1586
**54**	Mid log:**0 X** vs. Stationary:**0 X** Mid	3.488	3.103	0.3859	0.1586
**55**	log:**0 X** vs. Stationary:**10 X** Mid	3.488	2.874	0.6145	0.1586
**56**	log:**0 X** vs. Stationary:**25 X** Mid	3.488	2.455	1.033	0.1586
**57**	log:**10 X** vs. Mid log:**25 X**	3.330	3.111	0.2193	0.1586
**58**	Mid log:**10 X** vs. Late log:**0 X**	3.330	3.394	-0.06405	0.1586
**59**	Mid log:**10 X** vs. Late log:**10 X**	3.330	3.211	0.1198	0.1586
**60**	Mid log:**10 X** vs. Late log:**25 X** Mid	3.330	3.030	0.2999	0.1586
**61**	log:**10 X** vs. Stationary:**0 X** Mid	3.330	3.103	0.2278	0.1586
**62**	log:**10 X** vs. Stationary:**10 X** Mid	3.330	2.874	0.4565	0.1586
**63**	log:**10 X** vs. Stationary:**25 X** Mid	3.330	2.455	0.8749	0.1586
**64**	log:**25 X** vs. Late log:**0 X**	3.111	3.394	-0.2834	0.1586
**65**	Mid log:**25 X** vs. Late log:**10 X**	3.111	3.211	-0.09952	0.1586
**66**	Mid log:**25 X** vs. Late log:**25 X** Mid	3.111	3.030	0.08061	0.1586
**67**	log:**25 X** vs. Stationary:**0 X** Mid	3.111	3.103	0.008519	0.1586
**68**	log:**25 X** vs. Stationary:**10 X** Mid	3.111	2.874	0.2371	0.1586
**69**	log:**25 X** vs. Stationary:**25 X** Late	3.111	2.455	0.6556	0.1586
**70**	log:**0 X** vs. Late log:**10 X** Late log:**0**	3.394	3.211	0.1839	0.1586
**71**	**X** vs. Late log:**25 X** Late log:**0 X** vs.	3.394	3.030	0.3640	0.1586
**72**	Stationary:**0 X** Late log:**0 X** vs.	3.394	3.103	0.2919	0.1586
**73**	Stationary:**10 X** Late log:**0 X** vs.	3.394	2.874	0.5205	0.1586
**74**	Stationary:**25 X** Late log:**10 X** vs.	3.394	2.455	0.9389	0.1586
**75**	Late log:**25 X** Late log:**10 X** vs.	3.211	3.030	0.1801	0.1586
**76**	Stationary:**0 X** Late log:**10 X** vs.	3.211	3.103	0.1080	0.1586
**77**	Stationary:**10 X** Late log:**10 X** vs.	3.211	2.874	0.3367	0.1586
**78**	Stationary:**25 X** Late log:**25 X** vs.	3.211	2.455	0.7551	0.1586
**79**	Stationary:**0 X** Late log:**25 X** vs.	3.030	3.103	-0.07209	0.1586
**80**	Stationary:**10 X** Late log:**25 X** vs.	3.030	2.874	0.1565	0.1586
**81**	Stationary:**25 X** Stationary:**0 X** vs.	3.030	2.455	0.5750	0.1586
**82**	Stationary:**10 X** Stationary:**0 X** vs.	3.103	2.874	0.2286	0.1586
**83**	Stationary:**25 X** Stationary:**10 X** vs.	3.103	2.455	0.6470	0.1586
**84**	Stationary:**25 X**	2.874	2.455	0.4184	0.1586

Quantification of hydrogen peroxide
(2way ANOVA) Multiple Comparisons.

2way ANOVA<br>Multiple comparisons	A<br>Data Set-A<br>Y	B<br>Data Set-B<br>Y	C<br>Data Set-C<br>Y	D<br>Data Set-D<br>Y
Compare cell means regardless of rows and columns				
Number of families	1			
Number of comparisons per family	36			
Alpha	0.05			
Tukey's multiple comparisons test	Mean Diff.	95% CI of diff.	Significant?	Summary
11 h:**Control** vs. 11 h:**10 X**	-0.3390	-6.572 to 5.894	No	ns
11 h:**Control** vs. 11 h:**25 X**	1.003	-4.572 to 6.577	No	ns
11 h:**Control** vs. 14 h:**Control**	11.99	6.411 to 17.56	Yes	****
11 h:**Control** vs. 14 h:**10 X**	7.801	2.226 to 13.38	Yes	**
11 h:**Control** vs. 14 h:**25 X**	4.890	-0.6846 to 10.46	No	ns
11 h:**Control** vs. 17 h:**Control**	19.13	13.55 to 24.70	Yes	****
11 h:**Control** vs. 17 h:**10 X**	17.61	12.04 to 23.19	Yes	****
11 h:**Control** vs. 17 h:**25 X**	13.84	8.261 to 19.41	Yes	****
11 h:**10 X vs. 11** h:25 X	1.342	-4.891 to 7.574	No	ns
11 h:**10 X vs. 14** h:Control	12.33	6.092 to 18.56	Yes	****
11 h:**10 X vs. 14** h:10 X	8.140	1.907 to 14.37	Yes	**
11 h:**10 X vs. 14** h:25 X	5.229	-1.004 to 11.46	No	ns
11 h:**10 X vs. 17** h:Control	19.47	13.23 to 25.70	Yes	****
11 h:**10 X vs. 17** h:10 X	17.95	11.72 to 24.19	Yes	****
11 h:**10 X vs. 17** h:25 X	14.17	7.942 to 20.41	Yes	****
11 h:**25 X vs. 14** h:Control	10.98	5.409 to 16.56	Yes	****
11 h:**25 X vs. 14** h:10 X	6.798	1.224 to 12.37	Yes	*
11 h:**25 X vs. 14** h:25 X	3.888	-1.687 to 9.462	No	ns
11 h:**25 X vs. 17** h:Control	18.12	12.55 to 23.70	Yes	****
11 h:**25 X vs. 17** h:10 X	16.61	11.04 to 22.19	Yes	****
11 h:**25 X vs. 17** h:25 X	12.83	7.259 to 18.41	Yes	****
14 h:**Control** vs. 14 h:**10 X**	-4.185	-9.760 to 1.390	No	ns
14 h:**Control** vs. 14 h:**25 X**	-7.096	-12.67 to -1.521	Yes	**
14 h:**Control** vs. 17 h:**Control**	7.141	1.566 to 12.72	Yes	**
14 h:**Control** vs. 17 h:**10 X**	5.628	0.05284 to 11.20	Yes	*
14 h:**Control** vs. 17 h:**25 X**	1.850	-3.725 to 7.425	No	ns
14 h:**10 X vs. 14** h:25 X	-2.911	-8.486 to 2.664	No	ns
14 h:**10 X vs. 17** h:Control	11.33	5.752 to 16.90	Yes	****
14 h:**10 X vs. 17** h:10 X	9.813	4.238 to 15.39	Yes	***
14 h:**10 X vs. 17** h:25 X	6.035	0.4604 to 11.61	Yes	*
14 h:**25 X vs. 17** h:Control	14.24	8.662 to 19.81	Yes	****
14 h:**25 X vs. 17** h:10 X	12.72	7.149 to 18.30	Yes	****
14 h:**25 X vs. 17** h:25 X	8.946	3.371 to 14.52	Yes	***
17 h:**Control** vs. 17 h:**10 X**	-1.514	-7.088 to 4.061	No	ns
17 h:**Control** vs. 17 h:**25 X**	-5.291	-10.87 to 0.2835	No	ns
17 h:**10 X vs. 17** h:25 X	-3.778	-9.352 to 1.797	No	ns
Test details	Mean 1	Mean 2	Mean Diff.	SE of diff.
11 h:**Control** vs. 11 h:**10 X**	22.49	22.83	-0.3390	1.766
11 h:**Control** vs. 11 h:**25 X**	22.49	21.49	1.003	1.580

	2way ANOVA<br>Multiple comparisons	A<br>Data Set-A<br>Y	B<br>Data Set-B<br>Y	C<br>Data Set-C<br>Y	D<br>Data Set-D<br>Y
**51**	11 h:**Control** vs. 14 h:**Control**	22.49	10.50	11.99	1.580
**52**	11 h:**Control** vs. 14 h:**10 X**	22.49	14.69	7.801	1.580
**53**	11 h:**Control** vs. 14 h:**25 X**	22.49	17.60	4.890	1.580
**54**	11 h:**Control** vs. 17 h:**Control**	22.49	3.364	19.13	1.580
**55**	11 h:**Control** vs. 17 h:**10 X**	22.49	4.877	17.61	1.580
**56**	11 h:**Control** vs. 17 h:**25 X**	22.49	8.655	13.84	1.580
**57**	11 h:**10 X vs. 11** h:25 X	22.83	21.49	1.342	1.766
**58**	11 h:**10 X vs. 14** h:Control	22.83	10.50	12.33	1.766
**59**	11 h:**10 X vs. 14** h:10 X	22.83	14.69	8.140	1.766
**60**	11 h:**10 X vs. 14** h:25 X	22.83	17.60	5.229	1.766
**61**	11 h:**10 X vs. 17** h:Control	22.83	3.364	19.47	1.766
**62**	11 h:**10 X vs. 17** h:10 X	22.83	4.877	17.95	1.766
**63**	11 h:**10 X vs. 17** h:25 X	22.83	8.655	14.17	1.766
**64**	11 h:**25 X vs. 14** h:Control	21.49	10.50	10.98	1.580
**65**	11 h:**25 X vs. 14** h:10 X	21.49	14.69	6.798	1.580
**66**	11 h:**25 X vs. 14** h:25 X	21.49	17.60	3.888	1.580
**67**	11 h:**25 X vs. 17** h:Control	21.49	3.364	18.12	1.580
**68**	11 h:**25 X vs. 17** h:10 X	21.49	4.877	16.61	1.580
**69**	11 h:**25 X vs. 17** h:25 X	21.49	8.655	12.83	1.580
**70**	14 h:**Control** vs. 14 h:**10 X**	10.50	14.69	-4.185	1.580
**71**	14 h:**Control** vs. 14 h:**25 X**	10.50	17.60	-7.096	1.580
**72**	14 h:**Control** vs. 17 h:**Control**	10.50	3.364	7.141	1.580
**73**	14 h:**Control** vs. 17 h:**10 X**	10.50	4.877	5.628	1.580
**74**	14 h:**Control** vs. 17 h:**25 X**	10.50	8.655	1.850	1.580
**75**	14 h:**10 X vs. 14** h:25 X	14.69	17.60	-2.911	1.580
**76**	14 h:**10 X vs. 17** h:Control	14.69	3.364	11.33	1.580
**77**	14 h:**10 X vs. 17** h:10 X	14.69	4.877	9.813	1.580
**78**	14 h:**10 X vs. 17** h:25 X	14.69	8.655	6.035	1.580
**79**	14 h:**25 X vs. 17** h:Control	17.60	3.364	14.24	1.580
**80**	14 h:**25 X vs. 17** h:10 X	17.60	4.877	12.72	1.580
**81**	14 h:**25 X vs. 17** h:25 X	17.60	8.655	8.946	1.580
**82**	17 h:**Control** vs. 17 h:**10 X**	3.364	4.877	-1.514	1.580
**83**	17 h:**Control** vs. 17 h:**25 X**	3.364	8.655	-5.291	1.580
**84**	17 h:**10 X vs. 17** h:25 X	4.877	8.655	-3.778	1.580

Quantification of Malondialdehyde (MDA)
(2way ANOVA) Multiple Comparisons.

	**2way ANOVA** Multiple comparisons	**A** Data Set-A **Y**	**B** Data Set-B **Y**	**C** Data Set-C **Y**	**D** Data Set-D **Y**
**1**	Compare cell means regardless of rows and columns				
**2**					
**3**	Number of families	1			
**4**	Number of comparisons per family	36			
**5**	Alpha	0.05			
**6**					
**7**	Tukey's multiple comparisons test	Mean Diff.	95% CI of diff.	Significant?	Summary
**8**					
**9**	11 h:**Control** vs. 11 h:**10 X**	-0.5339	-1.136 to 0.06793	No	ns
**10**	11 h:**Control** vs. 11 h:**25 X**	-0.3272	-0.9290 to 0.2746	No	ns
**11**	11 h:**Control** vs. 14 h:**Control**	0.1119	-0.4899 to 0.7138	No	ns
**12**	11 h:**Control** vs. 14 h:**10 X**	-0.4047	-1.007 to 0.1971	No	ns
**13**	11 h:**Control** vs. 14 h:**25 X**	-0.1739	-0.7757 to 0.4279	No	ns
**14**	11 h:**Control** vs. 17 h:**Control**	0.3186	-0.2832 to 0.9204	No	ns
**15**	11 h:**Control** vs. 17 h:**10 X**	0.6028	0.0009570 to 1.205	Yes	*
**16**	11 h:**Control** vs. 17 h:**25 X**	-0.07222	-0.6740 to 0.5296	No	ns
**17**	11 h:**10 X** vs. 11 h:**25 X**	0.2067	-0.4526 to 0.8659	No	ns
**18**	11 h:**10 X** vs. 14 h:**Control**	0.6458	-0.01343 to 1.305	No	ns
**19**	11 h:**10 X** vs. 14 h:**10 X**	0.1292	-0.5301 to 0.7884	No	ns
**20**	11 h:**10 X** vs. 14 h:**25 X**	0.3600	-0.2993 to 1.019	No	ns
**21**	11 h:**10 X** vs. 17 h:**Control**	0.8525	0.1932 to 1.512	Yes	**
**22**	11 h:**10 X** vs. 17 h:**10 X**	1.137	0.4774 to 1.796	Yes	**
**23**	11 h:**10 X** vs. 17 h:**25 X**	0.4617	-0.1976 to 1.121	No	ns
**24**	11 h:**25 X** vs. 14 h:**Control**	0.4392	-0.2201 to 1.098	No	ns
**25**	11 h:**25 X** vs. 14 h:**10 X**	-0.07750	-0.7368 to 0.5818	No	ns
**26**	11 h:**25 X** vs. 14 h:**25 X**	0.1533	-0.5059 to 0.8126	No	ns
**27**	11 h:**25 X** vs. 17 h:**Control**	0.6458	-0.01343 to 1.305	No	ns
**28**	11 h:**25 X** vs. 17 h:**10 X**	0.9300	0.2707 to 1.589	Yes	**
**29**	11 h:**25 X** vs. 17 h:**25 X**	0.2550	-0.4043 to 0.9143	No	ns
**30**	14 h:**Control** vs. 14 h:**10 X**	-0.5167	-1.176 to 0.1426	No	ns
**31**	14 h:**Control** vs. 14 h:**25 X**	-0.2858	-0.9451 to 0.3734	No	ns
**32**	14 h:**Control** vs. 17 h:**Control**	0.2067	-0.4526 to 0.8659	No	ns
**33**	14 h:**Control** vs. 17 h:**10 X**	0.4908	-0.1684 to 1.150	No	ns
**34**	14 h:**Control** vs. 17 h:**25 X**	-0.1842	-0.8434 to 0.4751	No	ns
**35**	14 h:**10 X** vs. 14 h:**25 X**	0.2308	-0.4284 to 0.8901	No	ns
**36**	14 h:**10 X** vs. 17 h:**Control**	0.7233	0.06407 to 1.383	Yes	*
**37**	14 h:**10 X** vs. 17 h:**10 X**	1.008	0.3482 to 1.667	Yes	**
**38**	14 h:**10 X** vs. 17 h:**25 X**	0.3325	-0.3268 to 0.9918	No	ns
**39**	14 h:**25 X** vs. 17 h:**Control**	0.4925	-0.1668 to 1.152	No	ns
**40**	14 h:**25 X** vs. 17 h:**10 X**	0.7767	0.1174 to 1.436	Yes	*
**41**	14 h:**25 X** vs. 17 h:**25 X**	0.1017	-0.5576 to 0.7609	No	ns
**42**	17 h:**Control** vs. 17 h:**10 X**	0.2842	-0.3751 to 0.9434	No	ns
**43**	17 h:**Control** vs. 17 h:**25 X**	-0.3908	-1.050 to 0.2684	No	ns
**44**	17 h:**10 X** vs. 17 h:**25 X**	-0.6750	-1.334 to -0.01574	Yes	*
**45**					
**46**					
**47**	Test details	Mean 1	Mean 2	Mean Diff.	SE of diff.
**48**					
**49**	11 h:**Control** vs. 11 h:**10 X**	1.223	1.757	-0.5339	0.1559
**50**	11 h:**Control** vs. 11 h:**25 X**	1.223	1.550	-0.3272	0.1559

	2way ANOVA<br>Multiple comparisons	A<br>Data Set-A<br>Y	B<br>Data Set-B<br>Y	C<br>Data Set-C<br>Y	D<br>Data Set-D<br>Y
**51**	11 h:**Control** vs. 14 h:**Control**	1.223	1.111	0.1119	0.1559
**52**	11 h:**Control** vs. 14 h:**10 X**	1.223	1.628	-0.4047	0.1559
**53**	11 h:**Control** vs. 14 h:**25 X**	1.223	1.397	-0.1739	0.1559
**54**	11 h:**Control** vs. 17 h:**Control**	1.223	0.9042	0.3186	0.1559
**55**	11 h:**Control** vs. 17 h:**10 X**	1.223	0.6200	0.6028	0.1559
**56**	11 h:**Control** vs. 17 h:**25 X**	1.223	1.295	-0.07222	0.1559
**57**	11 h:**10 X** vs. 11 h:**25 X**	1.757	1.550	0.2067	0.1707
**58**	11 h:**10 X** vs. 14 h:**Control**	1.757	1.111	0.6458	0.1707
**59**	11 h:**10 X** vs. 14 h:**10 X**	1.757	1.628	0.1292	0.1707
**60**	11 h:**10 X** vs. 14 h:**25 X**	1.757	1.397	0.3600	0.1707
**61**	11 h:**10 X** vs. 17 h:**Control**	1.757	0.9042	0.8525	0.1707
**62**	11 h:**10 X** vs. 17 h:**10 X**	1.757	0.6200	1.137	0.1707
**63**	11 h:**10 X** vs. 17 h:**25 X**	1.757	1.295	0.4617	0.1707
**64**	11 h:**25 X** vs. 14 h:**Control**	1.550	1.111	0.4392	0.1707
**65**	11 h:**25 X** vs. 14 h:**10 X**	1.550	1.628	-0.07750	0.1707
**66**	11 h:**25 X** vs. 14 h:**25 X**	1.550	1.397	0.1533	0.1707
**67**	11 h:**25 X** vs. 17 h:**Control**	1.550	0.9042	0.6458	0.1707
**68**	11 h:**25 X** vs. 17 h:**10 X**	1.550	0.6200	0.9300	0.1707
**69**	11 h:**25 X** vs. 17 h:**25 X**	1.550	1.295	0.2550	0.1707
**70**	14 h:**Control** vs. 14 h:**10 X**	1.111	1.628	-0.5167	0.1707
**71**	14 h:**Control** vs. 14 h:**25 X**	1.111	1.397	-0.2858	0.1707
**72**	14 h:**Control** vs. 17 h:**Control**	1.111	0.9042	0.2067	0.1707
**73**	14 h:**Control** vs. 17 h:**10 X**	1.111	0.6200	0.4908	0.1707
**74**	14 h:**Control** vs. 17 h:**25 X**	1.111	1.295	-0.1842	0.1707
**75**	14 h:**10 X** vs. 14 h:**25 X**	1.628	1.397	0.2308	0.1707
**76**	14 h:**10 X** vs. 17 h:**Control**	1.628	0.9042	0.7233	0.1707
**77**	14 h:**10 X** vs. 17 h:**10 X**	1.628	0.6200	1.008	0.1707
**78**	14 h:**10 X** vs. 17 h:**25 X**	1.628	1.295	0.3325	0.1707
**79**	14 h:**25 X** vs. 17 h:**Control**	1.397	0.9042	0.4925	0.1707
**80**	14 h:**25 X** vs. 17 h:**10 X**	1.397	0.6200	0.7767	0.1707
**81**	14 h:**25 X** vs. 17 h:**25 X**	1.397	1.295	0.1017	0.1707
**82**	17 h:**Control** vs. 17 h:**10 X**	0.9042	0.6200	0.2842	0.1707
**83**	17 h:**Control** vs. 17 h:**25 X**	0.9042	1.295	-0.3908	0.1707
**84**	17 h:**10 X** vs. 17 h:**25 X**	0.6200	1.295	-0.6750	0.1707

APX
(2way ANOVA) Multiple Comparisons.

	**2way ANOVA**<br>Multiple comparisons	**A**<br>Data Set-A<br>**Y**	**B**<br>Data Set-B<br>**Y**	**C**<br>Data Set-C<br>**Y**
**1**	Compare cell means regardless of rows and columns			
**2**				
**3**	Number of families	1		
**4**	Number of comparisons per family	36		
**5**	Alpha	0.05		
**6**				
**7**	Tukey's multiple comparisons test	Mean Diff.	95% CI of diff.	Significant?
**8**				
**9**	Early log:**0 X** vs. Early log:**10 X**	-14.77	-88.39 to 58.84	No
**10**	Early log:**0 X** vs. Early log:**25 X**	32.49	-27.62 to 92.59	No
**11**	Early log:**0 X** vs. Mid log:**0 X**	-7.727	-67.83 to 52.38	No
**12**	Early log:**0 X** vs. Mid log:**10 X**	-37.30	-97.40 to 22.81	No
**13**	Early log:**0 X** vs. Mid log:**25 X**	-61.48	-121.6 to -1.380	Yes
**14**	Early log:**0 X** vs. Stationary:**0 X**	-52.99	-113.1 to 7.116	No
**15**	Early log:**0 X** vs. Stationary:**10 X**	-85.88	-146.0 to -25.78	Yes
**16**	Early log:**0 X** vs. Stationary:**25 X**	-146.5	-220.1 to -72.86	Yes
**17**	Early log:**10 X** vs. Early log:**25 X**	47.26	-26.35 to 120.9	No
**18**	Early log:**10 X** vs. Mid log:**0 X**	7.047	-66.57 to 80.66	No
**19**	Early log:**10 X** vs. Mid log:**10 X**	-22.52	-96.14 to 51.09	No
**20**	Early log:**10 X** vs. Mid log:**25 X**	-46.71	-120.3 to 26.90	No
**21**	Early log:**10 X** vs. Stationary:**0 X**	-38.21	-111.8 to 35.40	No
**22**	Early log:**10 X** vs. Stationary:**10 X**	-71.11	-144.7 to 2.503	No
**23**	Early log:**10 X** vs. Stationary:**25 X**	-131.7	-216.7 to -46.70	Yes
**24**	Early log:**25 X** vs. Mid log:**0 X**	-40.21	-100.3 to 19.89	No
**25**	Early log:**25 X** vs. Mid log:**10 X**	-69.78	-129.9 to -9.677	Yes
**26**	Early log:**25 X** vs. Mid log:**25 X**	-93.97	-154.1 to -33.86	Yes
**27**	Early log:**25 X** vs. Stationary:**0 X**	-85.47	-145.6 to -25.37	Yes
**28**	Early log:**25 X** vs. Stationary:**10 X**	-118.4	-178.5 to -58.26	Yes
**29**	Early log:**25 X** vs. Stationary:**25 X**	-179.0	-252.6 to -105.3	Yes
**30**	Mid log:**0 X** vs. Mid log:**10 X**	-29.57	-89.67 to 30.53	No
**31**	Mid log:**0 X** vs. Mid log:**25 X**	-53.76	-113.9 to 6.347	No
**32**	Mid log:**0 X** vs. Stationary:**0 X**	-45.26	-105.4 to 14.84	No
**33**	Mid log:**0 X** vs. Stationary:**10 X**	-78.16	-138.3 to -18.05	Yes
**34**	Mid log:**0 X** vs. Stationary:**25 X**	-138.7	-212.4 to -65.14	Yes
**35**	Mid log:**10 X** vs. Mid log:**25 X**	-24.19	-84.29 to 35.92	No
**36**	Mid log:**10 X** vs. Stationary:**0 X**	-15.69	-75.80 to 44.41	No
**37**	Mid log:**10 X** vs. Stationary:**10 X**	-48.59	-108.7 to 11.52	No
**38**	Mid log:**10 X** vs. Stationary:**25 X**	-109.2	-182.8 to -35.57	Yes
**39**	Mid log:**25 X** vs. Stationary:**0 X**	8.496	-51.61 to 68.60	No
**40**	Mid log:**25 X** vs. Stationary:**10 X**	-24.40	-84.50 to 35.70	No
**41**	Mid log:**25 X** vs. Stationary:**25 X**	-84.99	-158.6 to -11.38	Yes
**42**	Stationary:**0 X** vs. Stationary:**10 X**	-32.90	-93.00 to 27.21	No
**43**	Stationary:**0 X** vs. Stationary:**25 X**	-93.49	-167.1 to -19.87	Yes
**44**	Stationary:**10 X** vs. Stationary:**25 X**	-60.59	-134.2 to 13.02	No
**45**				

	2way ANOVA<br>Multiple comparisons	A<br>Data Set-A<br>Y	B<br>Data Set-B<br>Y	C<br>Data Set-C<br>Y
46				
47	Test details	Mean 1	Mean 2	Mean Diff.
48				
49	Early log:0 X vs. Early log:10 X	75.80	90.57	-14.77
50	Early log:0 X vs. Early log:25 X	75.80	43.31	32.49
51	Early log:0 X vs. Mid log:0 X	75.80	83.53	-7.727
52	Early log:0 X vs. Mid log:10 X	75.80	113.1	-37.30
53	Early log:0 X vs. Mid log:25 X	75.80	137.3	-61.48
54	Early log:0 X vs. Stationary:0 X	75.80	128.8	-52.99
55	Early log:0 X vs. Stationary:10 X	75.80	161.7	-85.88
56	Early log:0 X vs. Stationary:25 X	75.80	222.3	-146.5
57	Early log:10 X vs. Early log:25 X	90.57	43.31	47.26
58	Early log:10 X vs. Mid log:0 X	90.57	83.53	7.047
59	Early log:10 X vs. Mid log:10 X	90.57	113.1	-22.52
60	Early log:10 X vs. Mid log:25 X	90.57	137.3	-46.71
61	Early log:10 X vs. Stationary:0 X	90.57	128.8	-38.21
62	Early log:10 X vs. Stationary:10 X	90.57	161.7	-71.11
63	Early log:10 X vs. Stationary:25 X	90.57	222.3	-131.7
64	Early log:25 X vs. Mid log:0 X	43.31	83.53	-40.21
65	Early log:25 X vs. Mid log:10 X	43.31	113.1	-69.78
66	Early log:25 X vs. Mid log:25 X	43.31	137.3	-93.97
67	Early log:25 X vs. Stationary:0 X	43.31	128.8	-85.47
68	Early log:25 X vs. Stationary:10 X	43.31	161.7	-118.4
69	Early log:25 X vs. Stationary:25 X	43.31	222.3	-179.0
70	Mid log:0 X vs. Mid log:10 X	83.53	113.1	-29.57
71	Mid log:0 X vs. Mid log:25 X	83.53	137.3	-53.76
72	Mid log:0 X vs. Stationary:0 X	83.53	128.8	-45.26
73	Mid log:0 X vs. Stationary:10 X	83.53	161.7	-78.16
74	Mid log:0 X vs. Stationary:25 X	83.53	222.3	-138.7
75	Mid log:10 X vs. Mid log:25 X	113.1	137.3	-24.19
76	Mid log:10 X vs. Stationary:0 X	113.1	128.8	-15.69
77	Mid log:10 X vs. Stationary:10 X	113.1	161.7	-48.59
78	Mid log:10 X vs. Stationary:25 X	113.1	222.3	-109.2
79	Mid log:25 X vs. Stationary:0 X	137.3	128.8	8.496
80	Mid log:25 X vs. Stationary:10 X	137.3	161.7	-24.40
81	Mid log:25 X vs. Stationary:25 X	137.3	222.3	-84.99
82	Stationary:0 X vs. Stationary:10 X	128.8	161.7	-32.90
83	Stationary:0 X vs. Stationary:25 X	128.8	222.3	-93.49
84	Stationary:10 X vs. Stationary:25 X	161.7	222.3	-60.59

GPX
(2way ANOVA) Multiple Comparisons.

	**2way ANOVA**<br>Multiple comparisons	**A**<br>Data Set-A<br>**Y**	**B**<br>Data Set-B<br>**Y**	**C**<br>Data Set-C<br>**Y**
**1**	Compare cell means regardless of rows and columns			
**2**				
**3**	Number of families	1		
**4**	Number of comparisons per family	36		
**5**	Alpha	0.05		
**6**				
**7**	Tukey's multiple comparisons test	Mean Diff.	95% CI of diff.	Significant?
**8**				
**9**	Early log:**0 X** vs. Early log:**10 X**	-40.31	-114.3 to 33.70	No
**10**	Early log:**0 X** vs. Early log:**25 X**	-25.89	-99.89 to 48.12	No
**11**	Early log:**0 X** vs. Mid Log:**0 X**	-0.5142	-74.52 to 73.49	No
**12**	Early log:**0 X** vs. Mid Log:**10 X**	-78.95	-153.0 to -4.942	Yes
**13**	Early log:**0 X** vs. Mid Log:**25 X**	-64.88	-138.9 to 9.128	No
**14**	Early log:**0 X** vs. Stationary:**0 X**	-54.76	-128.8 to 19.24	No
**15**	Early log:**0 X** vs. Stationary:**10 X**	-54.73	-128.7 to 19.28	No
**16**	Early log:**0 X** vs. Stationary:**25 X**	-174.2	-264.8 to -83.56	Yes
**17**	Early log:**10 X** vs. Early log:**25 X**	14.42	-59.59 to 88.42	No
**18**	Early log:**10 X** vs. Mid Log:**0 X**	39.79	-34.21 to 113.8	No
**19**	Early log:**10 X** vs. Mid Log:**10 X**	-38.64	-112.6 to 35.36	No
**20**	Early log:**10 X** vs. Mid Log:**25 X**	-24.57	-98.58 to 49.43	No
**21**	Early log:**10 X** vs. Stationary:**0 X**	-14.46	-88.46 to 59.55	No
**22**	Early log:**10 X** vs. Stationary:**10 X**	-14.42	-88.43 to 59.58	No
**23**	Early log:**10 X** vs. Stationary:**25 X**	-133.9	-224.5 to -43.26	Yes
**24**	Early log:**25 X** vs. Mid Log:**0 X**	25.37	-48.63 to 99.38	No
**25**	Early log:**25 X** vs. Mid Log:**10 X**	-53.06	-127.1 to 20.95	No
**26**	Early log:**25 X** vs. Mid Log:**25 X**	-38.99	-113.0 to 35.01	No
**27**	Early log:**25 X** vs. Stationary:**0 X**	-28.87	-102.9 to 45.13	No
**28**	Early log:**25 X** vs. Stationary:**10 X**	-28.84	-102.8 to 45.16	No
**29**	Early log:**25 X** vs. Stationary:**25 X**	-148.3	-238.9 to -57.67	Yes
**30**	Mid Log:**0 X** vs. Mid Log:**10 X**	-78.43	-152.4 to -4.428	Yes
**31**	Mid Log:**0 X** vs. Mid Log:**25 X**	-64.36	-138.4 to 9.642	No
**32**	Mid Log:**0 X** vs. Stationary:**0 X**	-54.25	-128.3 to 19.76	No
**33**	Mid Log:**0 X** vs. Stationary:**10 X**	-54.21	-128.2 to 19.79	No
**34**	Mid Log:**0 X** vs. Stationary:**25 X**	-173.7	-264.3 to -83.05	Yes
**35**	Mid Log:**10 X** vs. Mid Log:**25 X**	14.07	-59.93 to 88.07	No
**36**	Mid Log:**10 X** vs. Stationary:**0 X**	24.18	-49.82 to 98.19	No
**37**	Mid Log:**10 X** vs. Stationary:**10 X**	24.22	-49.79 to 98.22	No
**38**	Mid Log:**10 X** vs. Stationary:**25 X**	-95.25	-185.9 to -4.616	Yes
**39**	Mid Log:**25 X** vs. Stationary:**0 X**	10.12	-63.89 to 84.12	No
**40**	Mid Log:**25 X** vs. Stationary:**10 X**	10.15	-63.86 to 84.15	No
**41**	Mid Log:**25 X** vs. Stationary:**25 X**	-109.3	-200.0 to -18.69	Yes
**42**	Stationary:**0 X** vs. Stationary:**10 X**	0.03439	-73.97 to 74.04	No
**43**	Stationary:**0 X** vs. Stationary:**25 X**	-119.4	-210.1 to -28.80	Yes
**44**	Stationary:**10 X** vs. Stationary:**25 X**	-119.5	-210.1 to -28.83	Yes
**45**				

	2way ANOVA<br>Multiple comparisons	A<br>Data Set-A<br>Y	B<br>Data Set-B<br>Y	C<br>Data Set-C<br>Y
46				
47	Test details	Mean 1	Mean 2	Mean Diff.
48				
49	Early log:**0 X** vs. Early log:**10 X**	78.07	118.4	-40.31
50	Early log:**0 X** vs. Early log:**25 X**	78.07	104.0	-25.89
51	Early log:**0 X** vs. Mid Log:**0 X**	78.07	78.58	-0.5142
52	Early log:**0 X** vs. Mid Log:**10 X**	78.07	157.0	-78.95
53	Early log:**0 X** vs. Mid Log:**25 X**	78.07	142.9	-64.88
54	Early log:**0 X** vs. Stationary:**0 X**	78.07	132.8	-54.76
55	Early log:**0 X** vs. Stationary:**10 X**	78.07	132.8	-54.73
56	Early log:**0 X** vs. Stationary:**25 X**	78.07	252.3	-174.2
57	Early log:**10 X** vs. Early log:**25 X**	118.4	104.0	14.42
58	Early log:**10 X** vs. Mid Log:**0 X**	118.4	78.58	39.79
59	Early log:**10 X** vs. Mid Log:**10 X**	118.4	157.0	-38.64
60	Early log:**10 X** vs. Mid Log:**25 X**	118.4	142.9	-24.57
61	Early log:**10 X** vs. Stationary:**0 X**	118.4	132.8	-14.46
62	Early log:**10 X** vs. Stationary:**10 X**	118.4	132.8	-14.42
63	Early log:**10 X** vs. Stationary:**25 X**	118.4	252.3	-133.9
64	Early log:**25 X** vs. Mid Log:**0 X**	104.0	78.58	25.37
65	Early log:**25 X** vs. Mid Log:**10 X**	104.0	157.0	-53.06
66	Early log:**25 X** vs. Mid Log:**25 X**	104.0	142.9	-38.99
67	Early log:**25 X** vs. Stationary:**0 X**	104.0	132.8	-28.87
68	Early log:**25 X** vs. Stationary:**10 X**	104.0	132.8	-28.84
69	Early log:**25 X** vs. Stationary:**25 X**	104.0	252.3	-148.3
70	Mid Log:**0 X** vs. Mid Log:**10 X**	78.58	157.0	-78.43
71	Mid Log:**0 X** vs. Mid Log:**25 X**	78.58	142.9	-64.36
72	Mid Log:**0 X** vs. Stationary:**0 X**	78.58	132.8	-54.25
73	Mid Log:**0 X** vs. Stationary:**10 X**	78.58	132.8	-54.21
74	Mid Log:**0 X** vs. Stationary:**25 X**	78.58	252.3	-173.7
75	Mid Log:**10 X** vs. Mid Log:**25 X**	157.0	142.9	14.07
76	Mid Log:**10 X** vs. Stationary:**0 X**	157.0	132.8	24.18
77	Mid Log:**10 X** vs. Stationary:**10 X**	157.0	132.8	24.22
78	Mid Log:**10 X** vs. Stationary:**25 X**	157.0	252.3	-95.25
79	Mid Log:**25 X** vs. Stationary:**0 X**	142.9	132.8	10.12
80	Mid Log:**25 X** vs. Stationary:**10 X**	142.9	132.8	10.15
81	Mid Log:**25 X** vs. Stationary:**25 X**	142.9	252.3	-109.3
82	Stationary:**0 X** vs. Stationary:**10 X**	132.8	132.8	0.03439
83	Stationary:**0 X** vs. Stationary:**25 X**	132.8	252.3	-119.4
84	Stationary:**10 X** vs. Stationary:**25 X**	132.8	252.3	-119.5
