# Supplementary material for: Tolerance of Pseudomonas strain to the 2,4-D herbicide through a peroxidase system
Source: PLoS One. 2021 Dec 2;16(12):e0257263. doi: 10.1371/journal.pone.0257263 (PMC8638965; doi:10.1371/journal.pone.0257263)
Supplement: S1 Raw images — (DOCX) [file pone.0257263.s002.docx]

**S1 Raw images**

**Original Images for Gels**

The capture of the images of the gels was made by were scanned using an ImageScanner III LabScan 6.0 (GE Healthcare).

**Superoxide dismutase (SOD)**


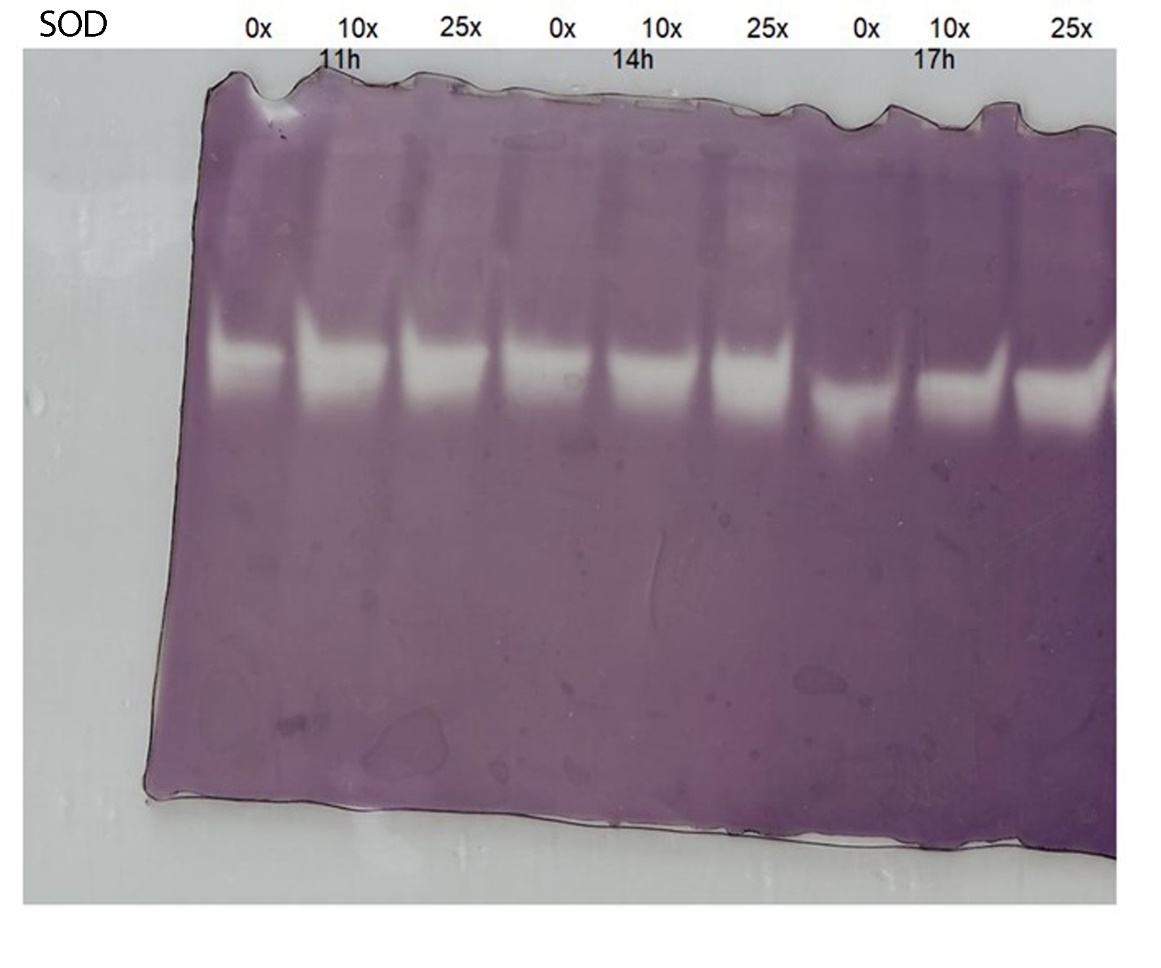


Image used for assembly of Fig. 6.

Protein extracts obtained from the bacteria *Pseudomonas* sp. CMA-7.3 were submitted to reveal bands with specific activity of the SOD enzyme.

**Superoxide dismutase (SOD) isoforms**


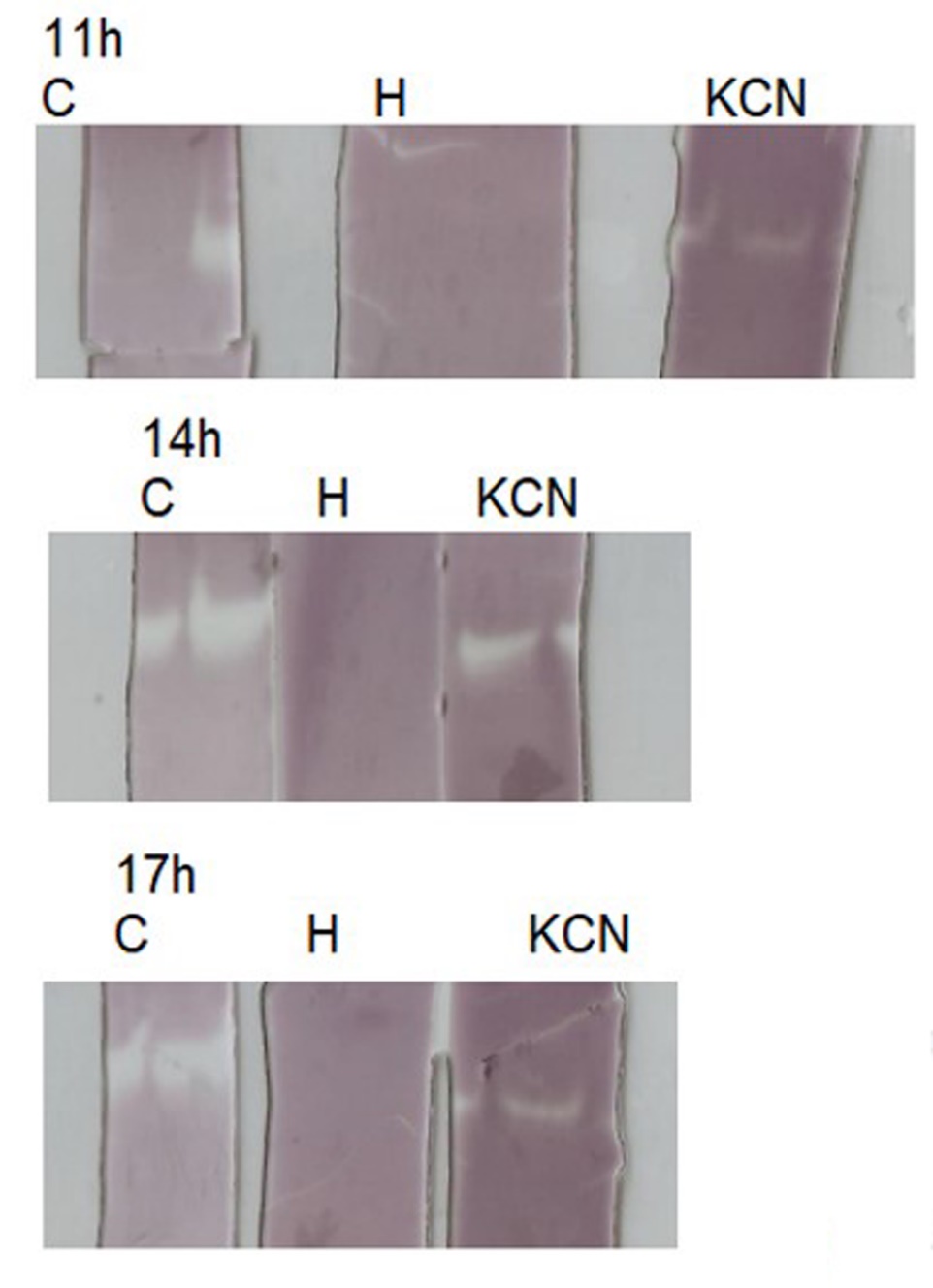


**C: Control**

**H: Hydrogen peroxide**

**KCN: Potassium cyanide**

Image used for assembly of Fig. 6. Protein extracts obtained from the bacteria *Pseudomonas* sp. CMA-7.3 were submitted to reveal bands with specific activity of the SOD enzyme.

**Catalase (CAT) isoforms**


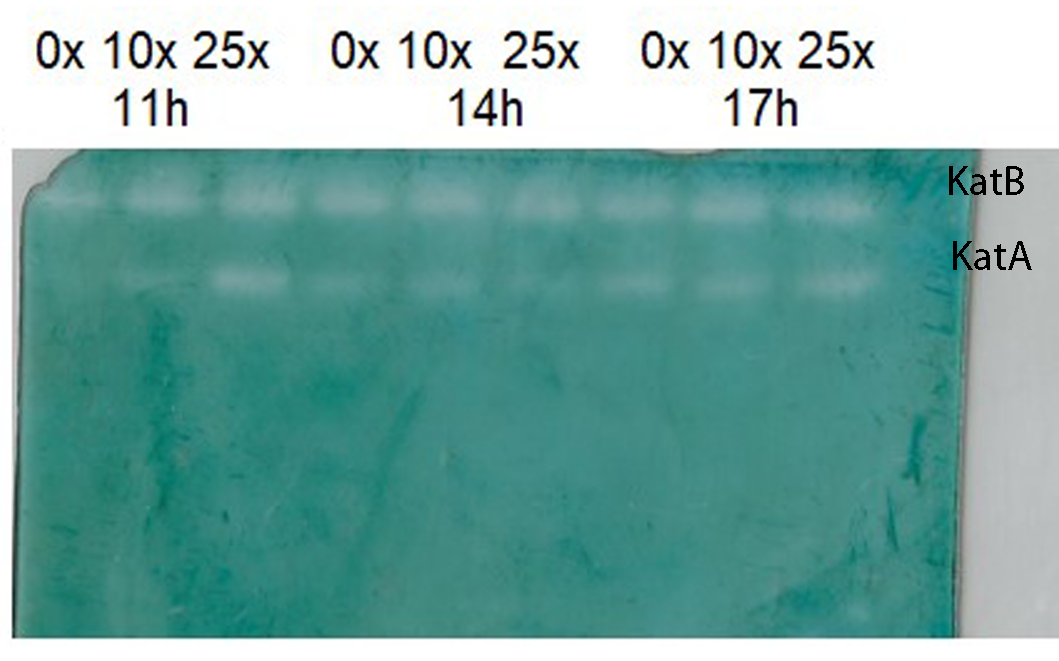


Image used for assembly of Fig. 7.

Protein extracts obtained from the bacteria *Pseudomonas* sp. CMA-7.3 were submitted to reveal bands with specific activity of the CAT enzyme.
