# Supplementary material for: Tolerance of Pseudomonas strain to the 2,4-D herbicide through a peroxidase system
Source: PLoS One. 2021 Dec 2;16(12):e0257263. doi: 10.1371/journal.pone.0257263 (PMC8638965; doi:10.1371/journal.pone.0257263)
Supplement: Attachment — Submitted filename: Tolerance of Pseudomonas Strain to the 2,4-D Herbicide plos one.doc [file pone.0257263.s003.doc]

Reviewer Response

The original manuscript titled ‘**Tolerance of *Pseudomonas Strain* to the 2,4-D Herbicide through a Peroxidase System**’, manuscript number ‘**PONE-D-21-27759**’ is an excellent research work and written in a good manner. The manuscript can be considered for publication process after meeting out the essential inclusions and improvements in the details provided. Hence, the authors are kindly requested to do the following,

1. Abstract section needs to modified and rewritten even more effectively avoiding few unnecessary explanations.
2. Provide better keywords in maximum numbers as per journal guidelines.
3. The introduction section can be presented with information of harmful effect of 2,4-D on human health, and can be improved with good research statement.
4. The results and discussion section needs to be modified. The authors can concentrate in providing more supporting discussion for the results obtained in the study. The discussion part is insufficient for understanding the results.
5. Give details on the mechanism of how *Pseudomonas Strain* managed to tolerate 2,4-D herbicide using Peroxidase System with your experiments.
6. The manuscript has detailed the results captured, but the effective explanations of the research results are missing; kindly update it under each respective header.
7. Conclusion section can be made even better and reachable with your obtained results.
8. The references are needed to be concentrated and aligned properly.
9. The entire alignment of manuscript content is inappropriate, it needs to be corrected.
10. Kindly correct the tenses of language used under each section and also correct the spellings.
