# Supplementary material for: Tolerance of Pseudomonas strain to the 2,4-D herbicide through a peroxidase system
Source: PLoS One. 2021 Dec 2;16(12):e0257263. doi: 10.1371/journal.pone.0257263 (PMC8638965; doi:10.1371/journal.pone.0257263)
Supplement: Attachment — Submitted filename: Manuscript Number PONE-D-21-27759.docx [file pone.0257263.s004.docx]

**Manuscript Number: PONE-D-21-27759**

**Tolerance of Pseudomonas Strain to the 2,4-D Herbicide through a Peroxidase System**

**Reviewer Response:**

The manuscript needs following amendments;

Line#122: DNA polymerase is always written in Units like 0.5Units.

Line#123: Kindly, mention the total reaction mixture volume.

Line#124/125: Kindly, mention thermocycler cycles with stages like Initial Denaturation at 95℃/2mins, for 40 cycles: Denaturation at 95℃/30 sec, Annealing at 57.7℃/30sec, Extension at 72℃/3min; Final-Extension at 72℃/5mins & 4℃/∞.

Line#145: Kindly, repeat the above-mentioned suggestion for this section also.

Line#212: MDA stands for?

Line#279/298/315/340/372/412/443/457: As per Journal’s Format, Figure Legends are to be mentioned at end of the text.

Line#279: Kindly, correct the figure legend font as per Journal’s Format.

Line#392: Kindly, correct “as ah response system”, as “as response system”.
